# Supplementary material for: Isolation of Scalimides A–L: β-Alanine-Bearing Scalarane Analogs from the Marine Sponge Spongia sp
Source: Mar Drugs. 2022 Nov 18;20(11):726. doi: 10.3390/md20110726 (PMC9695134; doi:10.3390/md20110726)
Supplement: Supplementary file 1 [file marinedrugs-20-00726-s001.zip › SI-MD-I.pdf]

Supporting Information I

Isolation of Scalimides A-L: *β*-Alanine-bearing Scalarane Alkaloids from the Marine Sponge *Spongia* sp.

Table of contents

Table S1. ^{13}C (150 MHz) and ^{1}H (600 MHz) NMR data for scalimide A (**1**) 3
Table S2. ^{13}C (150 MHz) and ^{1}H (600 MHz) NMR data for scalimide B (**2**).. 4
Table S3. ^{13}C (150 MHz) and ^{1}H (600 MHz) NMR data for scalimide C (**3**).. 5
Table S4. ^{13}C (150 MHz) and ^{1}H (600 MHz) NMR data for scalimide D (**4**). 6
Table S5. ^{13}C (150 MHz) and ^{1}H (600 MHz) NMR data for scalimide E (**5**).. 7
Table S6. ^{13}C (150 MHz) and ^{1}H (600 MHz) NMR data for scalimide F (**6**). 8
Table S7. ^{13}C (150 MHz) and ^{1}H (600 MHz) NMR data for scalimide G (**7**). 9
Table S8. ^{13}C (150 MHz) and ^{1}H (600 MHz) NMR data for scalimide H (**8**). 10
Table S9. ^{13}C (150 MHz) and ^{1}H (600 MHz) NMR data for scalimide I (**9**). 11
Table S10. ^{13}C (150 MHz) and ^{1}H (600 MHz) NMR data for scalimide J (**10**)...12
Table S11. ^{13}C (150 MHz) and ^{1}H (600 MHz) NMR data for scalimide K (**11**). 13
Table S12. ^{13}C (150 MHz) and ^{1}H (600 MHz) NMR data for scalimide L (**12**)...14
Figure S1. Structures of known compounds isolated from *Spongia* sp 15
Figure S2. ^{1}H-^{1}H COSY and HMBC correlations of scalimides A-L (**1-12**). 15
Figure S3. Key NOESY correlations of scalimides A-L (**1-12**). 16

Table S1. ^{13}C (150 MHz) and ^{1}H (600 MHz) NMR data for scalimide A (**1**).

Position	δ_C	δ_H (J, Hz)	^{1}H-^{1}H COSY	HMBC	NOESY
1	40.9	1.59, m<br>0.63, m	2		
2	19.5	1.65, m<br>1.41, m	1		
3	43.2	1.38, m<br>1.15, m			3α (1.15)-5/<br>3β (1.38)-20
4	34.2				
5	58.0	0.87, m	6		3α, 9
6	19.2	1.62, m<br>1.48, m	5, 7		6α (1.62)-19/<br>6β (1.48)-22
7	42.4	1.88, m<br>1.03, m	6	5, 9, 21	
8	38.6				
9	54.1	1.23, m	11		5, 14
10	38.1				
11	21.9	1.99, dd<br>(12.6, 6.9)<br>1.78, ddd<br>(15.2, 13.2, 2.4)	9, 12		11β (1.78)-21, 22
12	76.3	5.48, dd<br>(3.5, 2.7)	11	11	23
13	41.6				
14	51.3	1.66, m			9, 16
15	28.6	2.21, dd<br>(12.8, 6.9)<br>1.57, m	16	13, 14, 17	15β (1.57)-23
16	65.6	4.59, dd<br>(9.4, 7.0)	15	14, 17, 18, 24	14
17	141.9				
18	150.0				
19	33.7	0.87, s		3, 5	6α
20	21.7	0.84, s		3, 4, 5	3β
21	17.4	0.98, s		8, 9, 14	11β, 23
22	16.5	0.87, s		1, 5, 9, 10	6β, 11β
23	21.5	1.32, s		12, 13, 14, 18	12, 15β, 21
24	171.9				
25	170.4				
12-CH_3<u>C</u>O	172.1				
12-<u>C</u>H_3CO	21.1	1.94, s		12-CH_3<u>C</u>O	
1’	34.5	3.69, m	2’	24, 25, 3’	
2’	33.5	2.56, td<br>(6.9, 4.8)	1’	3’	
3’	174.4				

Table S2. ^{13}C (150 MHz) and ^{1}H (600 MHz) NMR data for scalimide B (**2**).

Position	δ_C	δ_H (*J*, Hz)	^1H-^1H COSY	HMBC	NOESY
1	40.9	1.61, m<br>0.65, m	2	5	
2	19.5	1.64, m<br>1.43, m	1	5	
3	43.2	1.38, m<br>1.17, m			
4	34.2				
5	58.0	0.92, m			9
6	19.2	1.63, m<br>1.48, m	7		
7	42.3	1.84, m<br>1.11, m	6		
8	38.3				
9	54.3	1.32, m	11		5, 14
10	38.1				
11	21.9	2.04, m<br>1.82, m	9, 12		11β (1.82)-22, 23
12	76.3	5.52, t<br>(2.9)	11	9, 14	23
13	41.7				
14	46.6	2.03, m		12, 16, 18	9
15	28.0	1.93, m<br>1.83, m	16	16, 17	
16	60.2	4.56, dd<br>(4.3, 1.5)	15	17, 18	
17	140.5				
18	151.1				
19	33.8	0.87, s		3, 5	
20	21.7	0.85, s		3, 4, 5	
21	17.6	0.96, s		7, 8, 9, 14	
22	16.4	0.88, s		1, 5, 9, 10	11β
23	20.1	1.21, s		12, 13, 14, 18	12, 21
24	171.0				
25	170.6				
12-CH_3<u>C</u>O	172.1				
12-<u>C</u>H_3CO	21.1	1.94, s		12-CH_3<u>C</u>O	
1’	34.5	3.70, t<br>(7.0)	2’	25, 3’	
2’	33.5	2.56, td<br>(6.9, 1.5)	1’	3’	
3’	174.4				

Table S3. ^{13}C (150 MHz) and ^{1}H (600 MHz) NMR data for scalimide C (**3**).

**Position**	δ_C	δ_H (***J*, Hz**)	^{1}**H-**^{1}**H COSY**	**HMBC**	**NOESY**
1	40.9	1.59, m<br>0.63, m	2	9, 22	1α (0.63)-9/<br>1β (1.59)-11α
2	19.5	1.63, m<br>1.40, m	1, 3		
3	43.2	1.38, m<br>1.15, td<br>(13.1, 3.9)	2		
4	34.2				
5	57.9	0.87, m	6		9
6	19.2	1.63, m<br>1.49, m	5, 7	5, 7	6β (1.49)-20
7	42.3	1.89, dt<br>(12.6, 3.5)<br>1.05, m	6	5, 9	7α (1.05)-9/<br>7β (1.89)-15α<br>(2.32)
8	38.7				
9	54.1	1.22, m	11		1α, 5, 7α, 14
10	38.1				
11	21.7	1.99, m<br>1.77, ddd<br>(15.2, 13.2, 2.4)	9, 12		11α (1.99)-1β/<br>11β (1.77)-23
12	76.1	5.48, dd<br>(3.2, 2.3)	11	9, 14, 23, 12-CH_3CO	23
13	41.5				
14	51.0	1.65, m		8, 15, 18	9, 16
15	25.5	2.32, dd<br>(12.8, 7.1)<br>1.59, m	16	13, 16, 17	15α (2.32)-7β
16	74.9	4.29, dd<br>(9.1, 7.0)	15	15, 17, 18, 16-OCH_3	14
17	140.6				
18	151.3				
19	33.7	0.87, s		3, 4, 5, 20	
20	21.7	0.85, s		3, 4, 5, 19	
21	17.4	0.98, s		7, 8, 9, 14	23
22	16.5	0.87, s		1, 9, 10	
23	21.4	1.31, s		12, 13, 14, 18	11β, 12, 21
24	170.2				
25	170.3				
12-$CH_3\underline{C}O$	171.8				
12-$\underline{C}H_3CO$	21.1	1.92, s		12-$CH_3\underline{C}O$	
16-$O\underline{C}H_3$	58.0	3.52, s		16	
1’	34.6	3.70, td<br>(6.9, 1.7)	2’	25, 2’, 3’	
2’	33.4	2.56, td<br>(6.9, 3.1)	1’	3’	
3’	174.4				

Table S4. ^{13}C (150 MHz) and ^{1}H (600 MHz) NMR data for scalimide D (**4**).

Position	δ_C	δ_H (*J*, Hz)	^1H-^1H COSY	HMBC	NOESY
1	40.9	1.59, m<br>0.65, td<br>(12.8, 3.7)		9, 22	1α (0.65)-9/<br>1β (1.59)-11α<br>(2.01)
2	19.5	1.63, m<br>1.41, m	3		
3	43.2	1.37, m<br>1.16, td<br>(13.4, 4.0)	2		
4	34.6				
5	58.0	0.88, m	6		
6	19.2	1.63, m<br>1.49, m	5, 7	5, 7	6β (1.49)-20
7	42.4	1.88, dt<br>(12.6, 3.0)<br>1.03, m	6	5, 9	7α (1.03)-9/<br>7β (1.88)-15α<br>(2.11)
8	38.3				
9	54.3	1.25, dd<br>(13.3, 2.3)	11		1α, 5, 7α, 14
10	38.1				
11	21.9	2.01, dt<br>(15.1, 3.0)<br>1.78, ddd<br>(15.2, 13.3, 2.5)	9, 12		11β (1.78)-23
12	76.2	5.49, dd<br>(3.5, 2.3)	11	9, 14, 23,<br>12-CH_3CO	23
13	41.8				
14	46.9	1.92, m	15	8, 15, 18	9
15	23.5	2.11, dd (14.1,<br>1.6)<br>1.63, m	14, 16	13, 16, 17	15α (2.11)-7β<br>15β (1.63)-23
16	70.1	4.18, dd<br>(4.0, 1.6)	15	15, 17, 18, 24,<br>16-OCH_3	
17	139.1				
18	151.7				
19	33.7	0.87, s		3, 4, 5, 20	
20	21.8	0.85, s		3, 4, 5, 19	6β
21	17.6	0.97, s		7, 8, 9, 14	23
22	16.4	0.88, s		1, 9, 10	
23	19.9	1.22, s		12, 13, 14, 18	11β, 15β, 12, 21
24	171.0				
25	170.5				
12-$CH_3\underline{C}O$	172.0				
12-$\underline{C}H_3CO$	21.1	1.94, s		12-$CH_3\underline{C}O$	
16-$O\underline{C}H_3$	57.9	3.46, s			
1’	34.6	3.70, td<br>(6.9, 2.1)	2’	25, 2’, 3’	
2’	33.5	2.56, td<br>(7.0, 5.0)	1’	3’	
3’	174.3				

Table S5. ^{13}C (150 MHz) and ^{1}H (600 MHz) NMR data for scalimide E (**5**).

Position	δ_C	δ_H (*J*, Hz)	^1H-^1H COSY	HMBC	NOESY
1	40.9	1.60, m<br>0.65, m	2		1α (0.65)-3α (1.17), 9/<br>1β (1.60)-11α (2.01)
2	19.5	1.65, m<br>1.42, m	1		
3	43.2	1.38, m<br>1.17, td (13.1, 3.9)		5	3α (1.17)-1α
4	34.2				
5	58.0	0.88, m	6		9
6	19.2	1.62, m<br>1.49, qd (13.2, 3.4)	5, 7		6β (1.49)-11β (1.78), 20, 21, 22
7	42.4	1.88, dt (12.7, 3.5)<br>1.03, td (12.7, 3.9)	6	5	7α (1.03)-9, 14/<br>7β (1.88)-15α (2.11)
8	38.3				
9	54.3	1.26, dd (13.4, 2.3)	11	8, 10	1α, 5, 7α, 14
10	38.1				
11	21.9	2.01, dt (14.8, 2.8)<br>1.78, ddd (15.2, 13.2, 2.5)	9, 12	9	11β (1.78)-6β, 23
12	76.1	5.49, t (2.9)	11	9, 14	23
13	41.8				
14	46.9	1.94, dd (12.9, 1.6)	15	16	7α, 9
15	23.5	2.11, m<br>1.62, m	14, 16	13, 16, 17	15α (2.11)-7β, 16-OCH_3
16	70.0	4.12, dd (4.0, 1.6)	15	14, 17, 18, 16-OCH_3	
17	139.2				
18	151.7				
19	33.8	0.87, s		3, 5, 20	
20	21.7	0.85, s		19	6β
21	17.6	0.97, s		8, 9, 14	6β, 23
22	16.4	0.88, s		5	6β,
23	19.9	1.22, s		12, 14, 18	11β, 12, 21
24	170.9				
25	170.5				
12-$CH_3\underline{C}O$	171.9				
12-$\underline{C}H_3CO$	21.1	1.96, s		12-$CH_3\underline{C}O$	
16-$O\underline{C}H_3$	57.9	3.46, s		16	15α
1’	34.6	3.72, t (6.7)	2’	25, 2’, 3’	
2’	33.8	2.56, td (6.7, 5.1)	1’	1’, 3’	
3’	173.0				
4’	52.3	3.63, s		3’	

Table S6. ^{13}C (150 MHz) and ^{1}H (600 MHz) NMR data for scalimide F (**6**).

Position	δ_C	δ_H (*J*, Hz)	^{1}H-^{1}H COSY	HMBC	NOESY
1	40.9	1.61, m<br>0.68, td<br>(12.7, 3.8)		9, 22	1α (0.68)-9/<br>1β (1.61)-22
2	19.5	1.66, m<br>1.42, m	3		
3	43.2	1.40, m<br>1.17, td<br>(14.0, 13.5, 4.2)	2		
4	34.3				
5	58.0	0.91, m	6		
6	18.9	1.65, m<br>1.53, m	5, 7		6α (1.65)-19/<br>6β (1.53)-20, 22
7	41.9	2.03, m<br>1.03, m	6		7α (1.03)-14
8	38.5				
9	53.4	1.31, m	11		1α, 5, 14
10	38.0				
11	22.1	2.06, m<br>1.76, ddd<br>(15.2, 13.2, 2.4)	9, 12	9	11β (1.76)-21, 23
12	74.7	5.44, dd<br>(3.5, 2.3)	11	14	23
13	41.9				
14	54.9	2.75, t<br>(3.0)	15	15, 16, 18	7α, 9
15	138.3	6.46, dd<br>(9.7, 2.7)	14, 16	14, 17	
16	117.7	6.39, dd<br>(9.7, 3.2)	15	14, 15, 17, 18	
17	137.7				
18	143.5				
19	33.8	0.88, s		3, 4, 5, 20	6β
20	21.8	0.85, s		3, 4, 5, 19	6α
21	19.3	1.09, s		7, 14	11β
22	16.4	0.89, s		1, 5, 9	1β, 6β
23	17.0	1.11, s		12, 14, 18	11β, 12
24	170.1				
25	170.5				
12-$CH_3\underline{C}O$	171.9				
12-$\underline{C}H_3CO$	21.2	1.99, s		12-$CH_3\underline{C}O$	
1’	34.6	3.72, t<br>(6.9)	2’	24, 25, 2’, 3’	
2’	33.5	2.57, td<br>(6.9, 3.1)	1’	3’	
3’	174.5				

Table S7. ^{13}C (150 MHz) and ^{1}H (600 MHz) NMR data for scalimide G (**7**).

**Position**	δ_C	δ_H **(*J*, Hz)**	**^{1}H-^{1}H COSY**	**HMBC**	**NOESY**
1	40.9	1.60, m<br>0.64, m		9, 10	1β (1.60)-11α (1.98)
2	19.5	1.64, m<br>1.40, m			
3	43.2	1.38, m<br>1.16, m			3β (1.38)-20
4	34.2				
5	58.1	0.87, m	6		9
6	19.2	1.60, m<br>1.49, qd (13.0, 3.3)	5, 7	5, 7	6β (1.49)-20
7	42.4	1.88, m<br>1.03, td (12.8, 3.9)	6		7α (1.03)-14/<br>7β (1.88)-15α (2.12)
8	38.3				
9	54.4	1.23, m	11	8, 10	5, 14
10	38.1				
11	21.9	1.98, m<br>1.75 ddd (15.2, 13.2, 2.4)	9, 12	9	11β (1.75)-21, 22, 23
12	75.9	5.56, t (2.8)	11	9, 14	23
13	40.7				11β, 12
14	47.5	1.79, m		16	7α, 9
15	22.8	2.12, d (14.4)<br>1.60, m	16		15α (2.12)-7β
16	71.3	4.02, dd (4.4, 1.3)	15	14, 17, 18, 16-OCH$_3$, 24	
17	151.4				
18	142.8				
19	33.8	0.88, s		3, 4, 5,	
20	21.8	0.85, s		5	
21	17.5	0.96, s		7, 8, 9, 14	
22	16.5	0.88, s		5, 9	
23	19.9	1.16, s		13, 14, 18	
24	81.7	5.39, d (1.9)			16α-OC$\underline{H}_3$
25	170.0				
12-CH$_3\underline{C}$O	172.2				
12-$\underline{C}$H$_3$CO	21.1	1.90, s		12-CH$_3\underline{C}$O	
16-O$\underline{C}$H$_3$	57.4	3.43, s		16	16α-OC$\underline{H}_3$-24
1’	36.2	3.68, m<br>3.53, dq (14.3, 7.2)	2’	24, 25, 3’	
2’	34.2	2.63, dt (16.4, 7.3)<br>2.53, dt (16.4, 6.4)	1’	3’	
3'	175.0				

Table S8. ^{13}C (150 MHz) and ^{1}H (600 MHz) NMR data for scalimide H (**8**).

Position	δ_C	δ_H (*J*, Hz)	^{1}H-^{1}H COSY	HMBC	NOESY
1	40.9	1.60, m<br>0.64, m			1α (1.60)-9/<br>1β (1.60)-11α (1.99)
2	19.5	1.64, m<br>1.40, m			
3	43.2	1.38, m<br>1.15, m			3β (1.38)-20
4	34.2				
5	58.0	0.88, m			9
6	19.2	1.62, m<br>1.49, qd (13.0, 3.3)	7		
7	42.5	1.88, m<br>1.01, td (12.9, 4.1)	6		7α (1.01)-14/<br>7β (1.88)-15α (2.13)
8	38.3				
9	54.4	1.23, m	11		1α, 5, 14
10	38.1				
11	21.8	1.99, dt (14.8, 3.0)<br>1.75, ddd (15.2, 13.2, 2.5)	9, 12		11α (1.99)-1β/<br>11β (1.75)-22, 23
12	75.8	5.57, t (2.8)	11		23
13	41.1				
14	47.3	1.81, dd (13.0, 7.1)	15	8, 13, 21	7α, 14
15	22.5	2.13, d (14.5)<br>1.64, m	14, 16	13, 17	15α (2.13)-7β
16	71.7	3.91, dd (4.4, 1.4)		14, 17, 18, 16-OCH_3	24-β-$OC\underline{H}_3$
17	148.8				
18	145.6				
19	33.8	0.88, s		3, 4, 5	
20	21.8	0.85, s		5	3β
21	17.5	0.97, s		7, 8, 9, 14	
22	16.5	0.88, s		5, 9	11β
23	20.1	1.18, s		13, 14, 18	11β, 12, 24-β-OCH_3
24	87.0	5.44, m		17, 18, 25	
25	170.3				
12-$CH_3\underline{C}O$	172.1				
12-$\underline{C}H_3CO$	21.1	1.90, s		12-$CH_3\underline{C}O$	
16-$O\underline{C}H_3$	57.4	3.43, s		16	
24-$O\underline{C}H_3$	50.5	3.04, s		24	16, 23
1’	36.6	3.69, m<br>3.40, m	2’	24, 25, 3’	
2’	33.5	2.64, ddd (16.4, 8.0, 6.4)<br>2.52, dt (16.4, 6.1)	1’	3’	
3’	174.9				

Table S9. ^{13}C (150 MHz) and ^{1}H (600 MHz) NMR data for scalimide I (**9**).

Position	δ_C	δ_H (J, Hz)	^1H-^1H COSY	HMBC	NOESY
1	40.9	1.61, m<br>0.65, m			1β (1.61)-11α (1.99)
2	19.5	1.66, m<br>1.42, m			
3	43.2	1.40, m<br>1.18, m			3β (1.40)-19
4	34.2				
5	58.0	0.89, m	6		7α (1.03), 9
6	19.2	1.64, m<br>1.51, m	5, 7		6α (1.64)-19/<br>6β (1.51)-21, 22
7	42.6	1.90, m<br>1.03, dt (13.1, 7.0)	6		7α (1.03)-5/<br>7β (1.90)-15α (2.14)
8	38.5				
9	54.3	1.23, dd (13.3, 2.3)	11		5, 7α, 14
10	38.1				
11	21.8	1.99, m<br>1.76, ddd (15.2, 13.2, 2.5)	9, 12		11α (1.99)-1α/<br>11β (1.76)-21, 22, 23
12	75.9	5.60, t (2.8)	11	14	23
13	40.9				
14	51.7	1.67, m			9, 16
15	28.9	2.14, dd (12.2, 6.6)<br>1.57, m	16	13, 16, 17	15α (2.14)-7β (1.90)/<br>15β (1.57)-23
16	68.6	4.44, dd (9.8, 6.6)	15	17, 18	14, 24
17	153.9				
18	140.1				
19	33.7	0.88, s		5	3β, 6α
20	21.7	0.86, s		3, 4, 5	
21	17.6	0.98, s		7, 8, 9, 14	6β, 11β, 23
22	16.4	0.88, s		5	6β, 11β
23	21.6	1.26, s		12, 13, 14, 18	11β, 12, 15β, 21
24	52.0	4.07, d (19.7)<br>3.97, d (19.8)		17, 18, 25	16
25	171.7				
12-$CH_3\underline{C}O$	172.3				
12-$\underline{C}H_3CO$	21.2	1.90, s		12-$CH_3\underline{C}O$	
1’	39.4	3.75, dt (13.4, 6.4)<br>3.58, dt (14.0, 6.7)	2’	25, 3’	
2’	34.1	2.60, dt (13.5, 6.6)	1’	3’	
3’	175.3				

Table S10. ^{13}C (150 MHz) and ^{1}H (600 MHz) NMR data for scalimide J (**10**).

Position	δ_C	δ_H (*J*, Hz)	^{1}H-^{1}H COSY	HMBC	NOESY
1	40.9	1.59, m<br>0.64, m			
2	19.5	1.65, m<br>1.41, m			
3	43.2	1.38, m<br>1.16, td<br>(13.2, 4.0)			3α (1.16)-19/<br>3β (1.38)-20
4	34.2				
5	58.0	0.88, m	6		9
6	19.2	1.63, m<br>1.49, m	5, 7		6α (1.63)-19/<br>6β (1.49)-20
7	42.5	1.89, m<br>1.04, m	6		7α (1.04)-9
8	38.6				
9	54.3	1.28, m	11	7, 12, 21	5, 7α, 14
10	38.1				
11	22.3	1.91, m<br>1.79, m	9, 12		
12	76.6	4.96, t<br>(2.8)	11	9, 14,<br>12-CH_3CO	23
13	42.3				
14	50.9	1.68, m		15	9, 16
15	28.6	2.15, dd<br>(12.8, 7.0)<br>1.53, m		17	
16	66.4	4.49, dd<br>(9.8, 6.7)		17, 18	14
17	133.4				
18	162.3				
19	33.7	0.88, s		3, 5	3α, 6α
20	21.7	0.85, s		3, 4, 5	3β, 6β
21	17.7	0.99, s		7, 8, 14	23
22	16.6	0.88, s		5, 10	
23	21.6	1.26, s		12, 13, 14, 18	12, 21, 25
24	172.1				
25	50.4	4.06, d<br>(19.6)<br>3.77, d<br>(19.6)		17, 18	23
12-CH_3CO	172.5				
12-CH_3CO	21.2	2.01, s		12-CH_3CO	
1’	39.3	3.74, m<br>3.58, dt<br>(13.7, 6.4)	2’	24, 3’	
2’	34.0	2.59, m	1’	3’	
3’	175.1				

Table S11. ^{13}C (150 MHz) and ^{1}H (600 MHz) NMR data for scalimide K (**11**).

Position	δ_C	δ_H (*J*, Hz)	^{1}H-^{1}H COSY	HMBC	NOESY
1	40.9	1.60, m<br>0.68, m	2		1α (0.68)-9/<br>1β (1.60)-11α (1.97)
2	19.6	1.66, m<br>1.42, m	1, 3		
3	43.2	1.39, dt (13.4, 3.8)<br>1.18, td (13.4, 4.1)	2		
4	34.3				
5	58.1	0.92, m	6		9
6	18.9	1.64, m<br>1.52, qd (12.8, 3.3)	5, 7		6α (1.64)-19/<br>6β (1.52)-20, 21, 22
7	42.0	2.02, td (12.6, 3.4)<br>1.03, m	6	5, 9	7α (1.03)-14
8	38.5				
9	53.5	1.35, dd (13.1, 2.4)	11	8, 10, 12	1α, 5, 14
10	38.1				
11	22.6	1.97, m<br>1.77, ddd (15.2, 13.0, 2.2)	9, 12	9	11α (1.97)-1β/<br>11β (1.77)-22, 23
12	75.1	5.05, dd (3.7, 2.1)	11	9, 13, 14, 12-CH_3CO	23
13	43.4				
14	54.1	2.68, d (3.0)	15	15, 16, 18, 23	7α, 9
15	130.3	6.03, dd (9.8, 2.6)	14, 16	14, 17	
16	118.8	6.25, dd (9.7, 3.3)	15	14, 15, 17, 18	
17	130.3				
18	159.8				
19	33.8	0.88, s		3, 4, 5, 20	6α
20	21.8	0.86, s		3, 4, 5, 19	6β
21	19.2	1.10, s		7, 8, 14	6β
22	16.4	0.89, s		1, 5, 10	6β, 11β
23	17.5	1.06, s		12, 13, 14, 18	11β, 12, 25
24	171.6				
25	50.5	4.06, m		17, 18, 25	23
12-$CH_3\underline{C}O$	172.0				
12-$\underline{C}H_3CO$	21.3	2.11, s		12-$CH_3\underline{C}O$	
1’	39.5	3.73, dt (14.1, 6.3)<br>3.65, m	2’	24, 25, 2’, 3’	
2’	34.1	2.61, td (6.8, 6.3, 5.1)	1’	3’	
3’	175.3				

Table S12. ^{13}C (150 MHz) and ^{1}H (600 MHz) NMR data for scalimide L (**12**).

Position	δ_C	δ_H (*J*, Hz)	^1H-^1H COSY	HMBC	NOESY
1	40.9	1.60, m<br>0.68, m	2		1β (1.60)-11α (1.96)
2	19.5	1.66, m<br>1.42, m	1, 3		
3	43.2	1.39, m<br>1.18, td (13.2, 3.9)	2		
4	34.3				
5	58.1	0.91, m	6		9
6	18.9	1.64, m<br>1.53, m	5, 7		6α (1.64)-19
7	41.9	2.02, m<br>1.03, m	6		7α (1.03)-14
8	38.5				
9	53.5	1.34, m	11		
10	38.1				
11	22.6	1.96, dd (14.8, 3.3)<br>1.77, m	9, 12		11α (1.96)-1β/ 11β (1.77)-21, 22, 23
12	75.0	5.06, s	11		23
13	43.4				
14	54.1	2.68, m	15		7α, 9
15	130.3	6.03, dd (9.7, 2.6)	14, 16		
16	118.8	6.24, dd (9.7, 3.3)	15	17	
17	130.3				
18	159.8				
19	33.8	0.88, s		3, 4, 5, 20	6α
20	21.8	0.86, s		3, 4, 5	
21	19.2	1.10, s		7, 8, 9, 14	11β
22	16.4	0.89, s		1, 5, 9, 10	11β
23	17.5	1.06, s		12, 13, 14, 18	11β, 12, 25
24	173.9				
25	50.4	4.09, m<br>4.01, m		17, 18	23
12-CH_3<u>C</u>O	172.4				
12-<u>C</u>H_3CO	21.3	2.13, s			
1’	39.4	3.74, m<br>3.66, m	2’	24	
2’	33.9	2.64, m	1’	1’, 3’	
3’	174.4				
4'	52.3	3.65, s		3’	

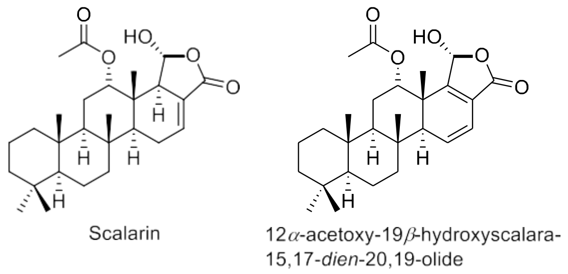

Figure S1. Structures of known compounds isolated from *Spongia* sp.

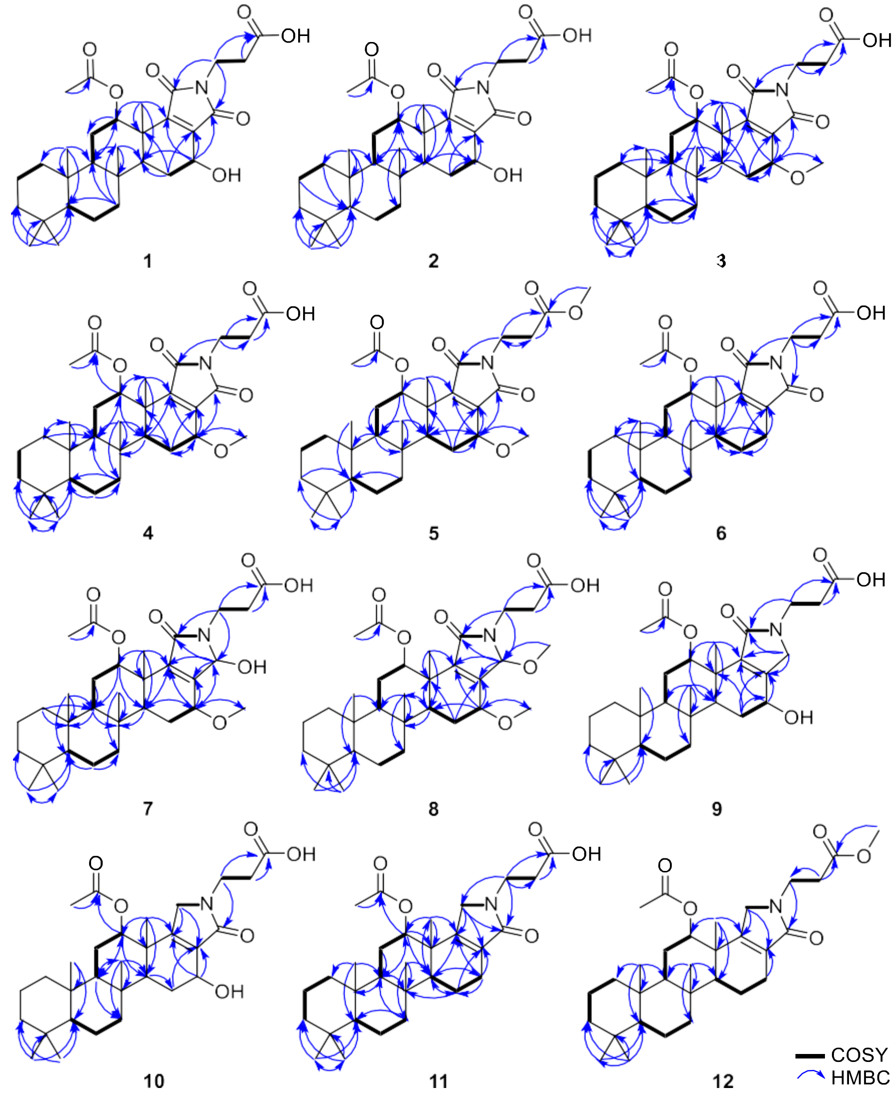

Figure S2. ^{1}H-^{1}H COSY and HMBC correlations of scalimides A-L (**1-12**).

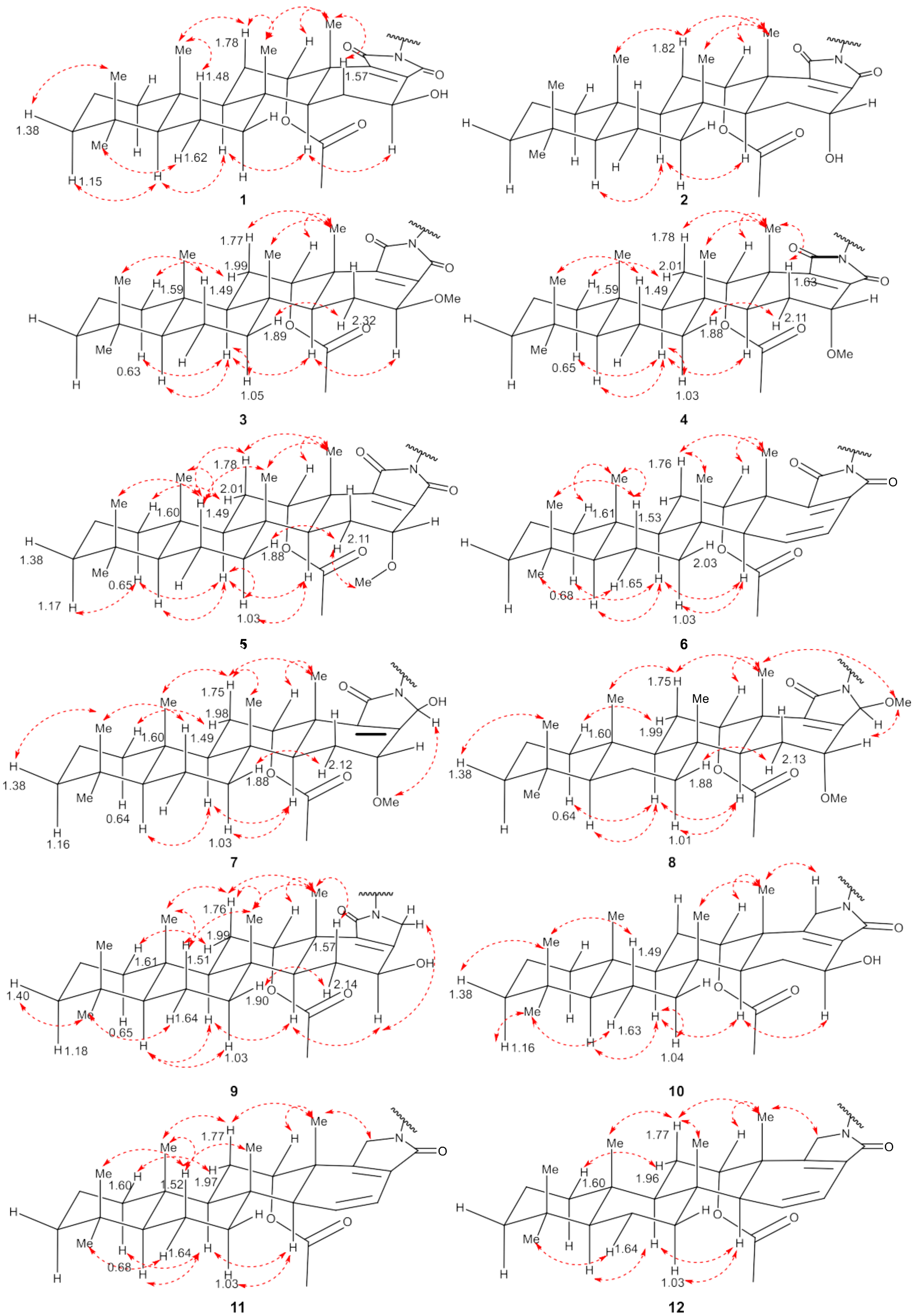

Figure S3. Key NOESY correlations of scalimides A-L (**1-12**).
