# Supplementary material for: Isolation of Scalimides A–L: β-Alanine-Bearing Scalarane Analogs from the Marine Sponge Spongia sp
Source: Mar Drugs. 2022 Nov 18;20(11):726. doi: 10.3390/md20110726 (PMC9695134; doi:10.3390/md20110726)
Supplement: Supplementary file 1 [file marinedrugs-20-00726-s001.zip › SI-MD-II.pdf]

Supporting Information II

NMR Spectra

Isolation of Scalimides A-L: *β*-Alanine-bearing Scalarane Alkaloids from the Marine Sponge *Spongia* sp.

Table of contents

Figure S4. ^{1}H NMR spectrum of scalimide A (**1**) in CD_3OD (600 MHz) 5
Figure S5. ^{13}C NMR spectrum of scalimide A (**1**) in CD_3OD (150 MHz) 5
Figure S6. HSQC spectrum of scalimide A (**1**) in CD_3OD (600 MHz) 6
Figure S7. ^{1}H-^{1}H COSY spectrum of scalimide A (**1**) in CD_3OD (600 MHz) 6
Figure S8. HMBC spectrum of scalimide A (**1**) in CD_3OD (600 MHz) 7
Figure S9. NOESY spectrum of scalimide A (**1**) in CD_3OD (600 MHz) 7
Figure S10. HRMS spectrum of scalimide A (**1**) 7
Figure S11. ^{1}H NMR spectrum of scalimide B (**2**) in CD_3OD (600 MHz) 8
Figure S12. ^{13}C NMR spectrum of scalimide B (**2**) in CD_3OD (150 MHz) 8
Figure S13. HSQC spectrum of scalimide B (**2**) in CD_3OD (600 MHz) 9
Figure S14. ^{1}H-^{1}H COSY spectrum of scalimide B (**2**) in CD_3OD (600 MHz) 9
Figure S15. HMBC spectrum of scalimide B (**2**) in CD_3OD (600 MHz) 10
Figure S16. NOESY spectrum of scalimide B (**2**) in CD_3OD (600 MHz) 10
Figure S17. HRMS spectrum of scalimide B (**2**). 10
Figure S18. ^{1}H NMR spectrum of scalimide C (**3**) in CD_3OD (600 MHz) 11
Figure S19. ^{13}C NMR spectrum of scalimide C (**3**) in CD_3OD (150 MHz) 11
Figure S20. HSQC spectrum of scalimide C (**3**) in CD_3OD (600 MHz) 12
Figure S21. ^{1}H-^{1}H COSY spectrum of scalimide C (**3**) in CD_3OD (600 MHz) 12
Figure S22. HMBC spectrum of scalimide C (**3**) in CD_3OD (600 MHz) 13
Figure S23. NOESY spectrum of scalimide C (**3**) in CD_3OD (600 MHz) 13
Figure S24. HRMS spectrum of scalimide C (**3**). 13
Figure S25. ^{1}H NMR spectrum of scalimide D (**4**) in CD_3OD (600 MHz) 14
Figure S26. ^{13}C NMR spectrum of scalimide D (**4**) in CD_3OD (150 MHz) 14
Figure S27. HSQC spectrum of scalimide D (**4**) in CD_3OD (600 MHz) 15
Figure S28. ^{1}H-^{1}H COSY spectrum of scalimide D (**4**) in CD_3OD (600 MHz) 15
Figure S29. HMBC spectrum of scalimide D (**4**) in CD_3OD (600 MHz) 16
Figure S30. NOESY spectrum of scalimide D (**4**) in CD_3OD (600 MHz) 16
Figure S31. HRMS spectrum of scalimide D (**4**) 16
Figure S32. ^{1}H NMR spectrum of scalimide E (**5**) in CD_3OD (600 MHz) 17
Figure S33. ^{13}C NMR spectrum of scalimide E (**5**) in CD_3OD (150 MHz) 17
Figure S34. HSQC spectrum of scalimide E (**5**) in CD_3OD (600 MHz) 18
Figure S35. ^{1}H-^{1}H COSY spectrum of scalimide E (**5**) in CD_3OD (600 MHz) 18
Figure S36. HMBC spectrum of scalimide E (**5**) in CD_3OD (600 MHz) 19
Figure S37. NOESY spectrum of scalimide E (**5**) in CD_3OD (600 MHz) 19
Figure S38. HRMS spectrum of scalimide E (**5**). 19
Figure S39. ^{1}H NMR spectrum of scalimide F (**6**) in CD_3OD (600 MHz) 20
Figure S40. ^{13}C NMR spectrum of scalimide F (**6**) in CD_3OD (150 MHz) 20
Figure S41. HSQC spectrum of scalimide F (**6**) in CD_3OD (600 MHz) 21

Figure S42. ^{1}H-^{1}H COSY spectrum of scalimide F (**6**) in CD_3OD (600 MHz) 21
Figure S43. HMBC spectrum of scalimide F (**6**) in CD_3OD (600 MHz)..............22
Figure S44. NOESY spectrum of scalimide F (**6**) in CD_3OD (600 MHz) 22
Figure S45. HRMS spectrum of scalimide F (**6**). 22
Figure S46. ^{1}H NMR spectrum of scalimide G (**7**) in CD_3OD (600 MHz) 23
Figure S47. ^{13}C NMR spectrum of scalimide G (**7**) in CD_3OD (150 MHz) 23
Figure S48. HSQC spectrum of scalimide G (**7**) in CD_3OD (600 MHz)..............24
Figure S49. ^{1}H-^{1}H COSY spectrum of scalimide G (**7**) in CD_3OD (600 MHz) 24
Figure S50. HMBC spectrum of scalimide G (**7**) in CD_3OD (600 MHz)..............25
Figure S51. NOESY spectrum of scalimide G (**7**) in CD_3OD (600 MHz) 25
Figure S52. HRMS spectrum of scalimide G (**7**)..............25
Figure S53. ^{1}H NMR spectrum of scalimide H (**8**) in CD_3OD (600 MHz) 26
Figure S54. ^{13}C NMR spectrum of scalimide H (**8**) in CD_3OD (150 MHz) 26
Figure S55. HSQC spectrum of scalimide H (**8**) in CD_3OD (600 MHz)..............27
Figure S56. ^{1}H-^{1}H COSY spectrum of scalimide H (**8**) in CD_3OD (600 MHz) 27
Figure S57. HMBC spectrum of scalimide H (**8**) in CD_3OD (600 MHz)..............28
Figure S58. NOESY spectrum of scalimide H (**8**) in CD_3OD (600 MHz) 28
Figure S59. 1D NOE spectrum of scalimide H (**8**)...............29
Figure S60. HRMS spectrum of scalimide H (**8**)..............29
Figure S61. ^{1}H NMR spectrum of scalimide I (**9**) in CD_3OD (600 MHz)..............30
Figure S62. ^{13}C NMR spectrum of scalimide I (**9**) in CD_3OD (150 MHz)..............30
Figure S63. HSQC spectrum of scalimide I (**9**) in CD_3OD (600 MHz)..............31
Figure S64. ^{1}H-^{1}H COSY spectrum of scalimide I (**9**) in CD_3OD (600 MHz)..............31
Figure S65. HMBC spectrum of scalimide I (**9**) in CD_3OD (600 MHz)..............32
Figure S66. NOESY spectrum of scalimide I (**9**) in CD_3OD (600 MHz)..............32
Figure S67. HRMS spectrum of scalimide I (**9**). 32
Figure S68. ^{1}H NMR spectrum of scalimide J (**10**) in CD_3OD (600 MHz)..............33
Figure S69. ^{13}C NMR spectrum of scalimide J (**10**) in CD_3OD (150 MHz)..............33
Figure S70. HSQC spectrum of scalimide J (**10**) in CD_3OD (600 MHz) 34
Figure S71. ^{1}H-^{1}H COSY spectrum of scalimide J (**10**) in CD_3OD (600 MHz)...............34
Figure S72. HMBC spectrum of scalimide J (**10**) in CD_3OD (600 MHz) 35
Figure S73. NOESY spectrum of scalimide J (**10**) in CD_3OD (600 MHz)..............35
Figure S74. HRMS spectrum of scalimide J (**10**)...............35
Figure S75. ^{1}H NMR spectrum of scalimide K (**11**) in CD_3OD (600 MHz). 36
Figure S76. ^{13}C NMR spectrum of scalimide K (**11**) in CD_3OD (150 MHz) 36
Figure S77. HSQC spectrum of scalimide K (**11**) in CD_3OD (600 MHz)..............37
Figure S78. ^{1}H-^{1}H COSY spectrum of scalimide K (**11**) in CD_3OD (600 MHz) 37
Figure S79. HMBC spectrum of scalimide K (**11**) in CD_3OD (600 MHz)..............38
Figure S80. NOESY spectrum of scalimide K (**11**) in CD_3OD (600 MHz) 38
Figure S81. HRMS spectrum of scalimide K (**11**)..............38

Figure S82. ^{1}H NMR spectrum of scalimide L (**12**) in CD_3OD (600 MHz) 39
Figure S83. ^{13}C NMR spectrum of scalimide L (**12**) in CD_3OD (150 MHz) 39
Figure S84. HSQC spectrum of scalimide L (**12**) in CD_3OD (600 MHz) 40
Figure S85. ^{1}H-^{1}H COSY spectrum of scalimide L (**12**) in CD_3OD (600 MHz) 40
Figure S86. HMBC spectrum of scalimide L (**12**) in CD_3OD (600 MHz) 41
Figure S87. NOESY spectrum of scalimide L (**12**) in CD_3OD (600 MHz) 41
Figure S88. HRMS spectrum of scalimide L (**12**). 41

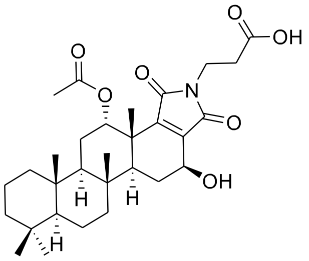

1

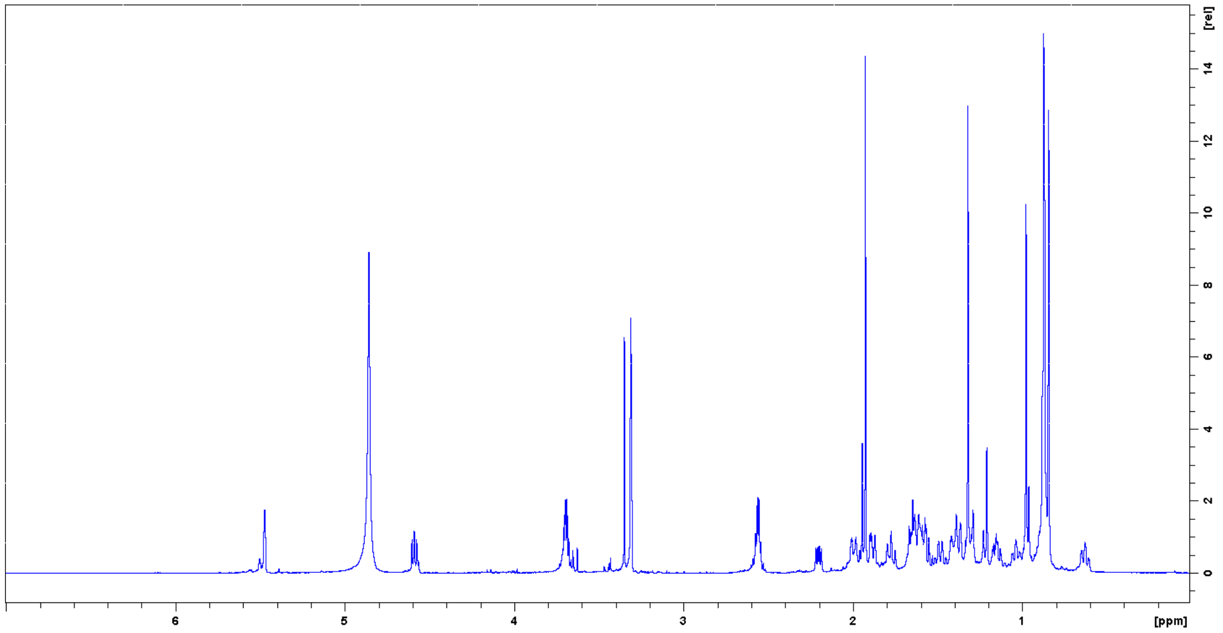

Figure S4. ^{1}H NMR spectrum of scalimide A (**1**) in CD_3OD (600 MHz).

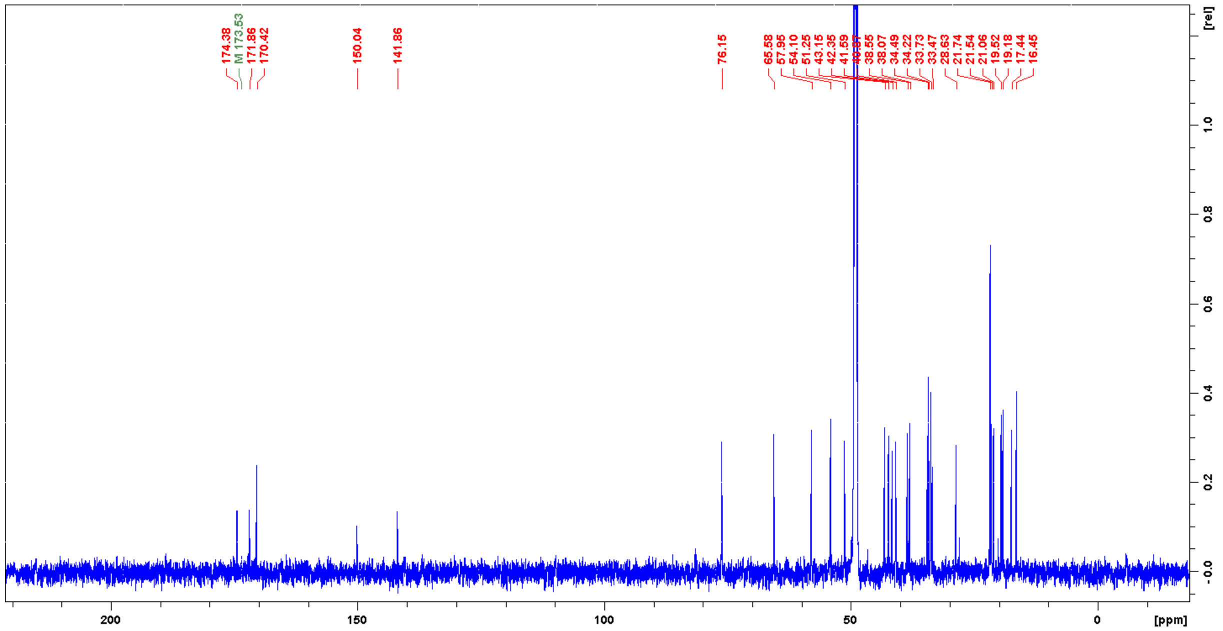

Figure S5. ^{13}C NMR spectrum of scalimide A (**1**) in CD_3OD (150 MHz).

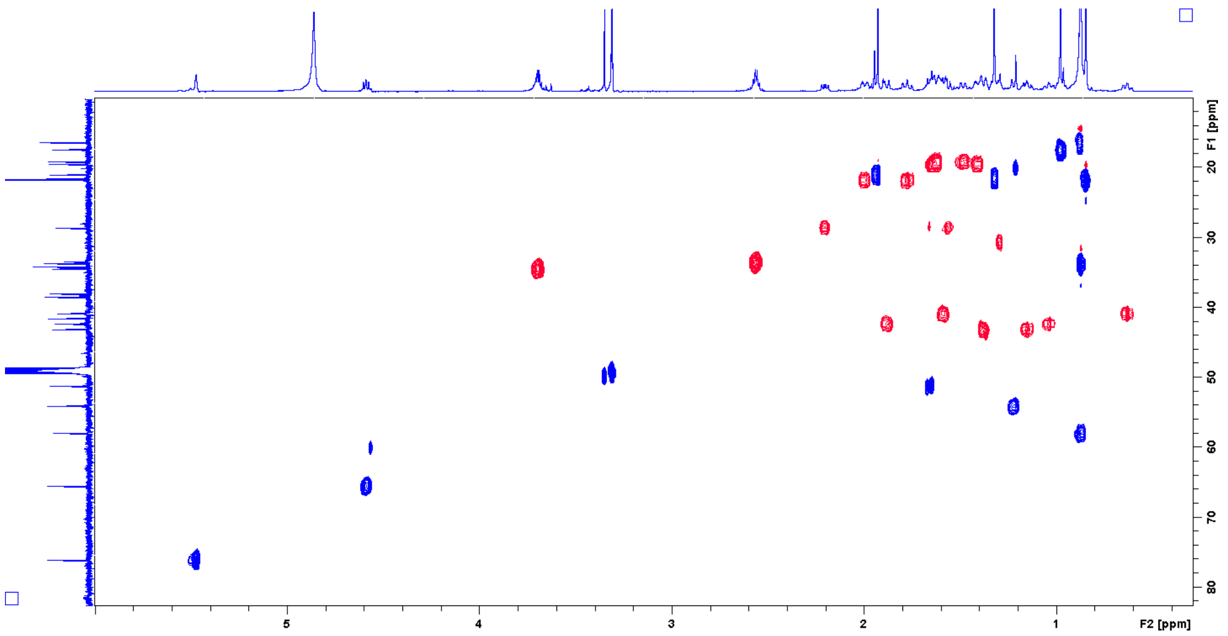

Figure S6. HSQC spectrum of scalimide A (**1**) in CD_3OD (600 MHz).

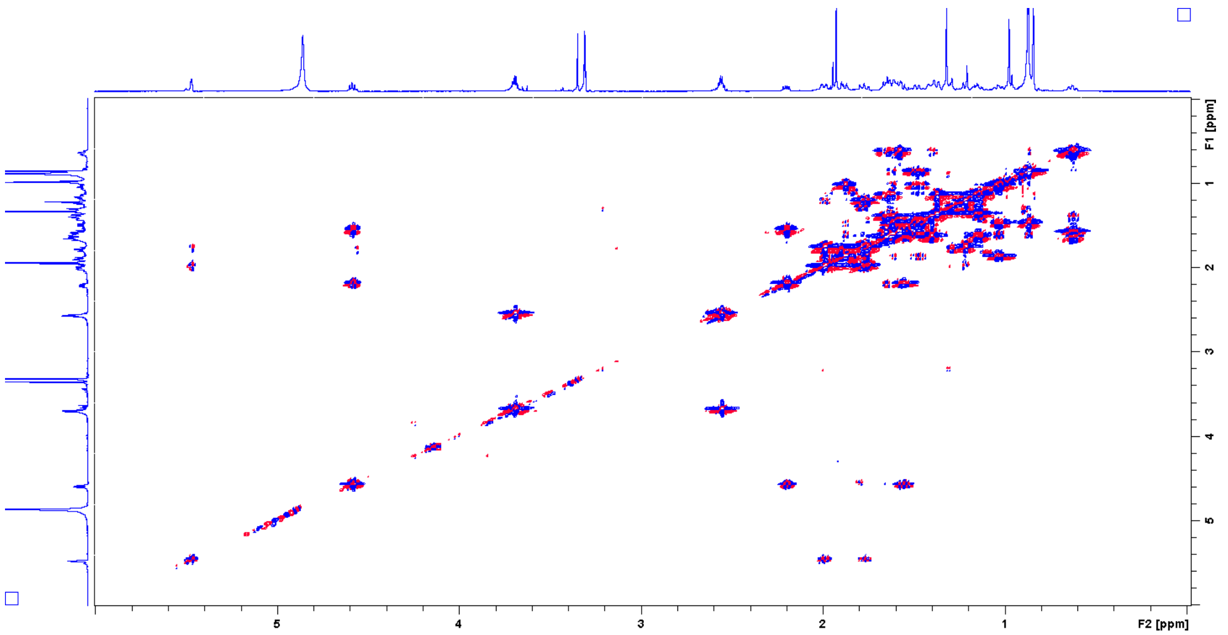

Figure S7. ^1H-^1H COSY spectrum of scalimide A (**1**) in CD_3OD (600 MHz).

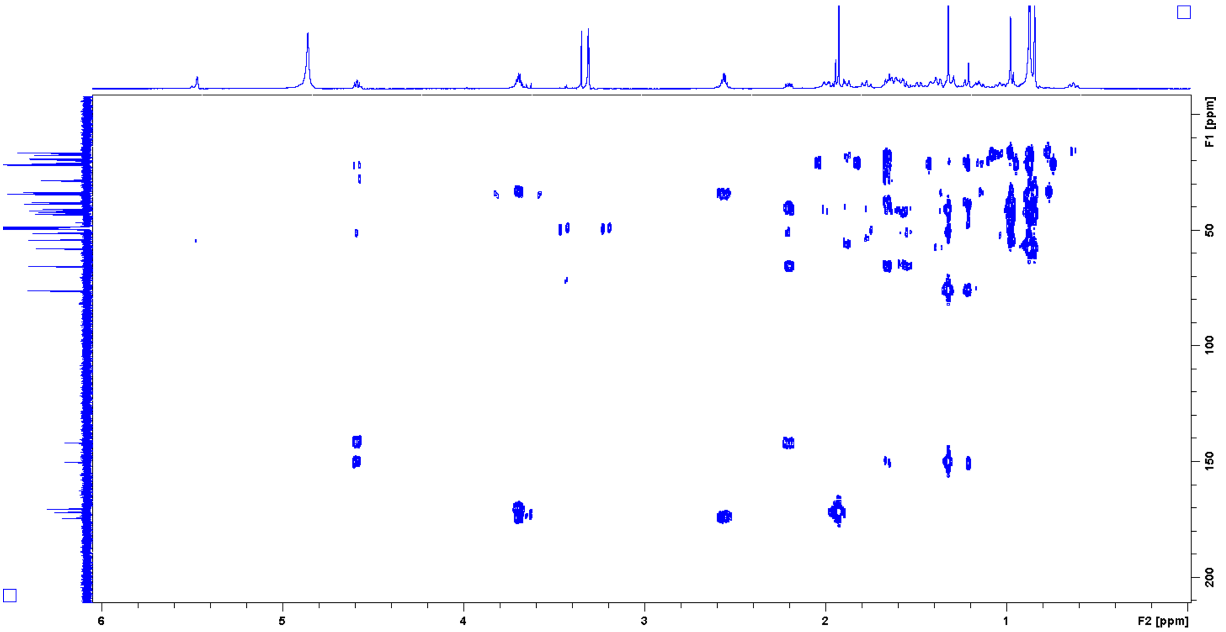

Figure S8. HMBC spectrum of scalimide A (**1**) in CD_3OD (600 MHz).

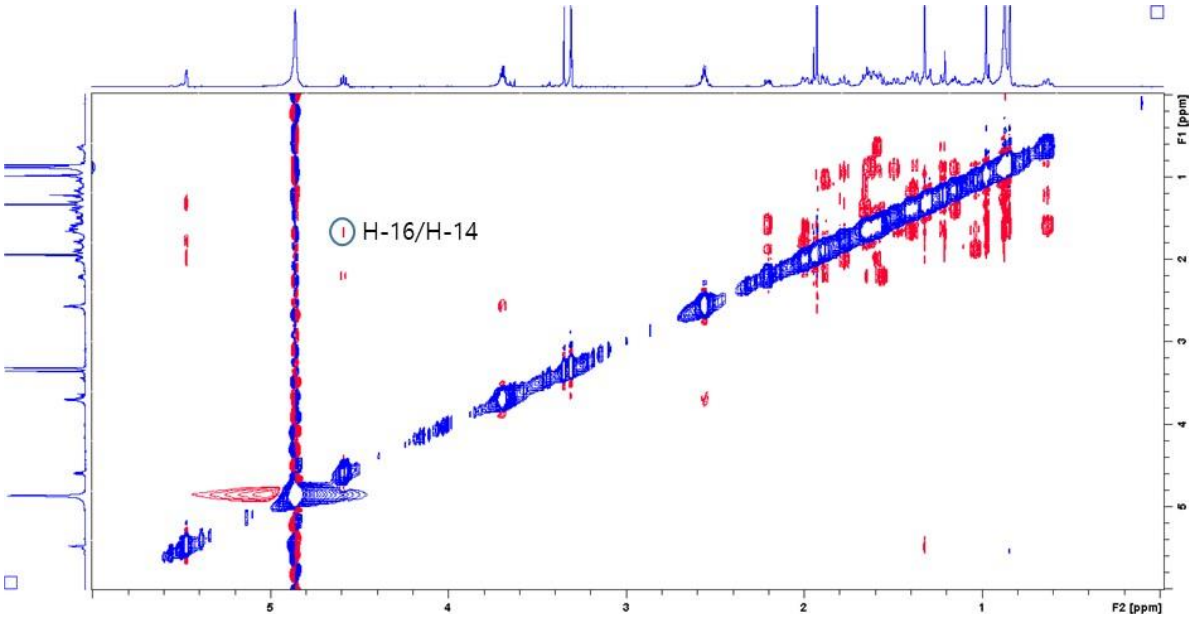

Figure S9. NOESY spectrum of scalimide A (**1**) in CD_3OD (600 MHz).

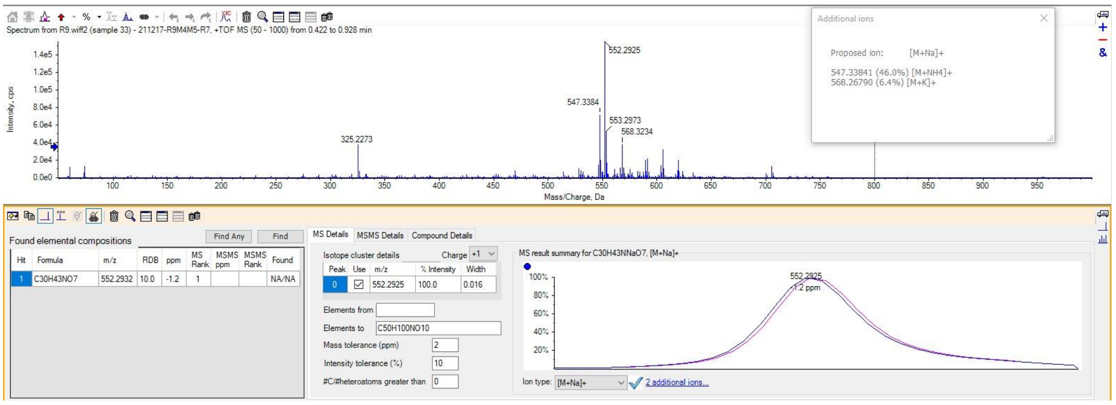

Figure S10. HRMS spectrum of scalimide A (**1**).

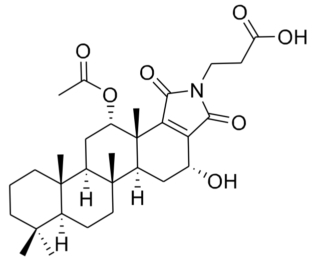

2

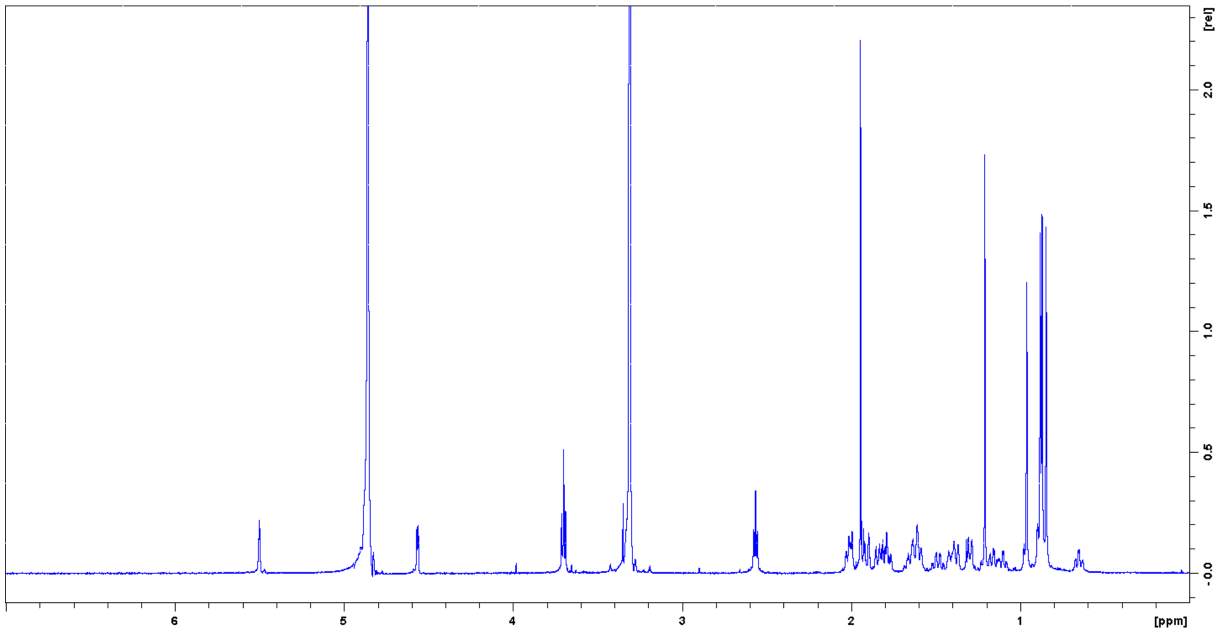

Figure S11. ^{1}H NMR spectrum of scalimide B (**2**) in CD_3OD (600 MHz).

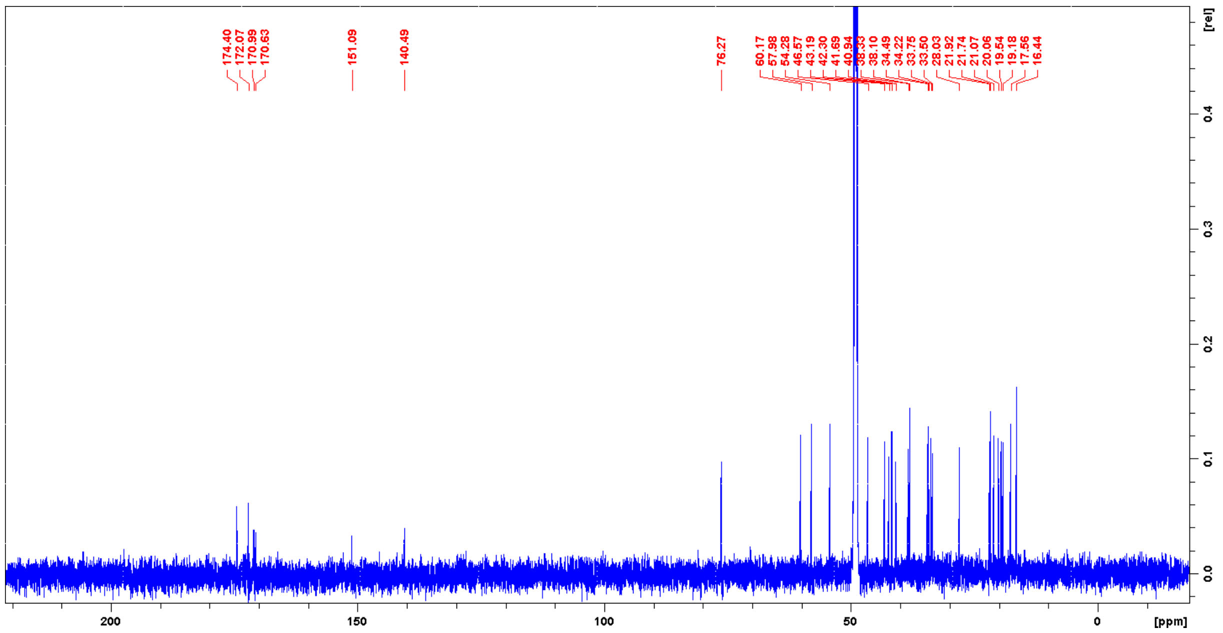

Figure S12. ^{13}C NMR spectrum of scalimide B (**2**) in CD_3OD (**1**50 MHz).

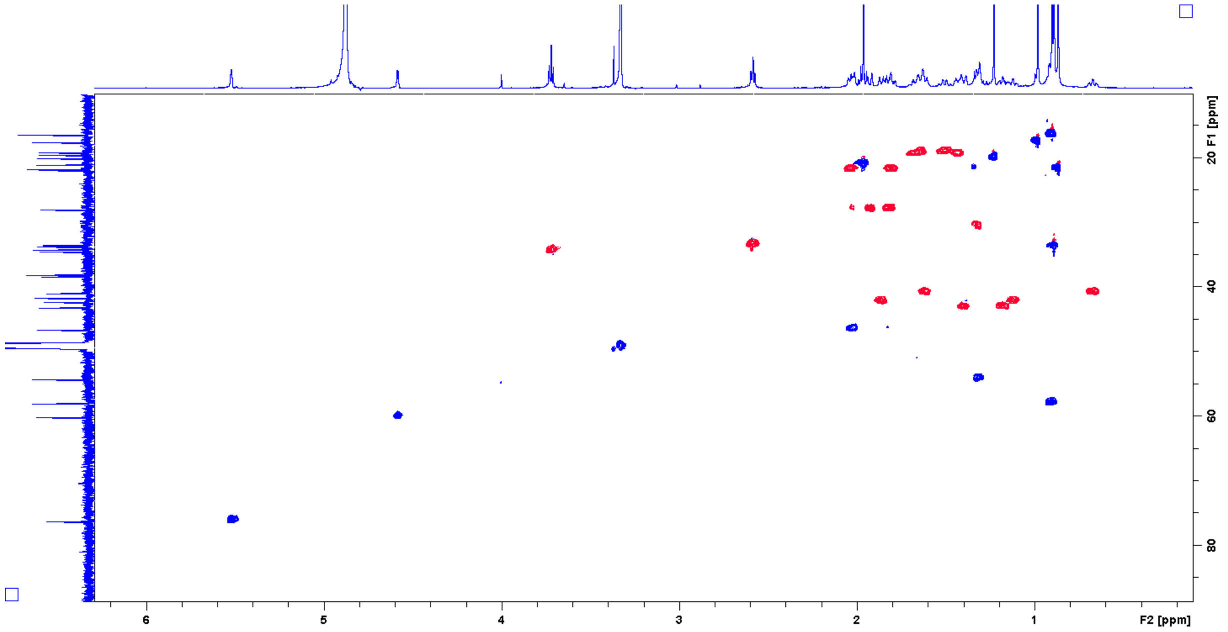

Figure S13. HSQC spectrum of scalimide B (**2**) in CD_3OD (600 MHz).

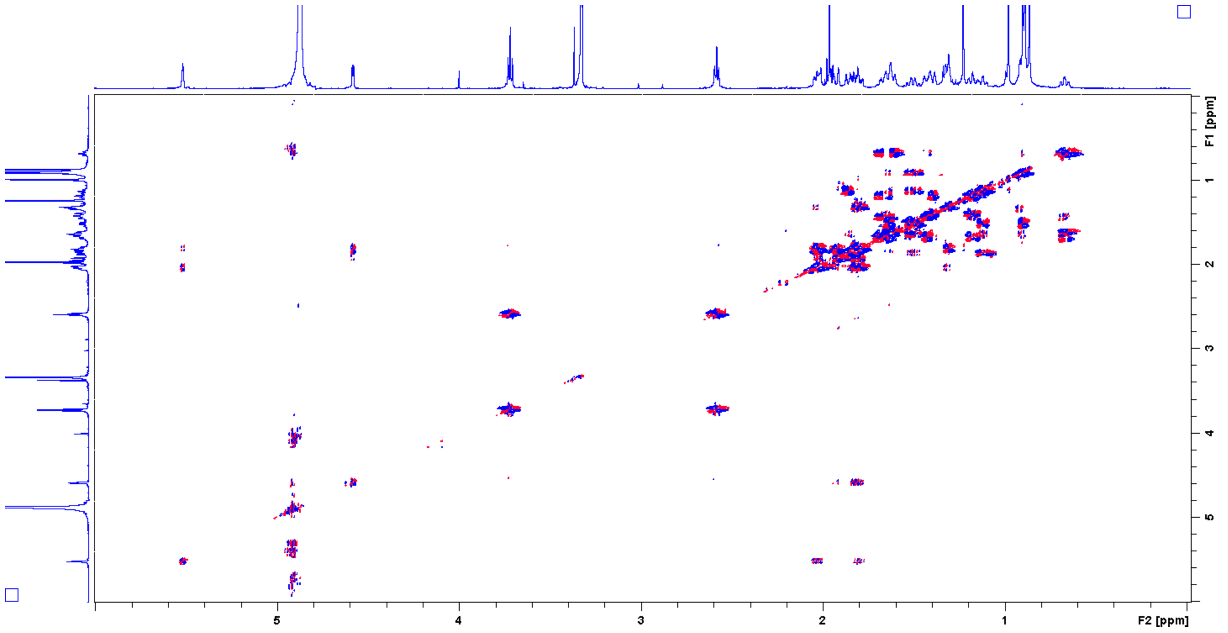

Figure S14. ^1H-^1H COSY spectrum of scalimide B (**2**) in CD_3OD (600 MHz).

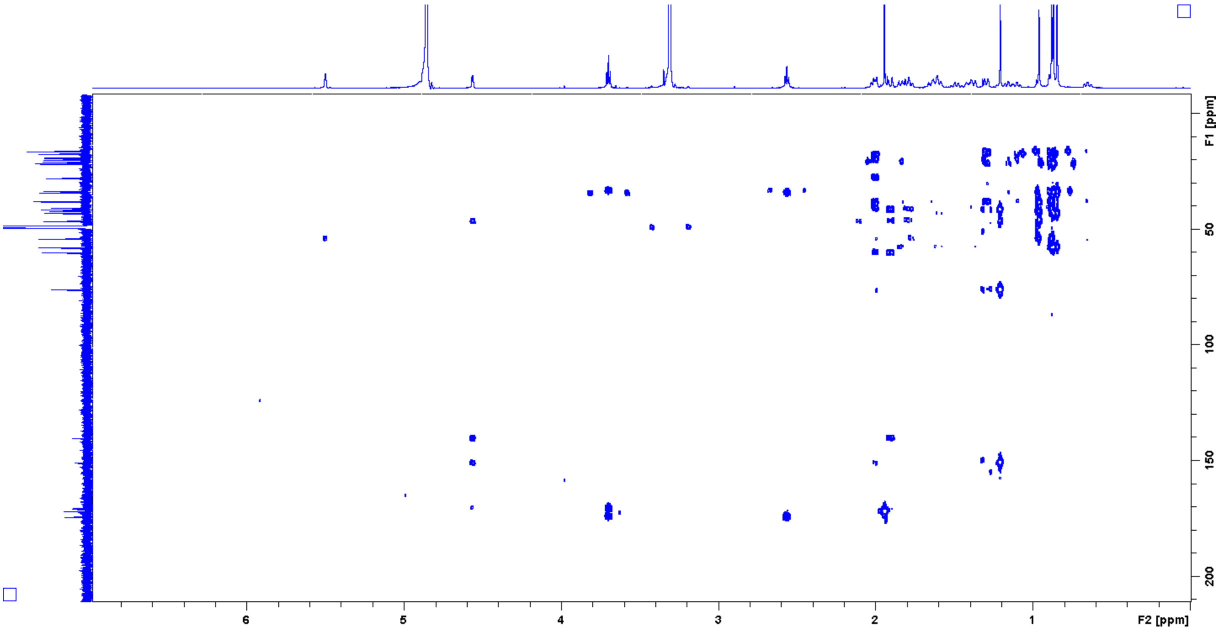

Figure S15. HMBC spectrum of scalimide B (**2**) in CD_3OD (600 MHz).

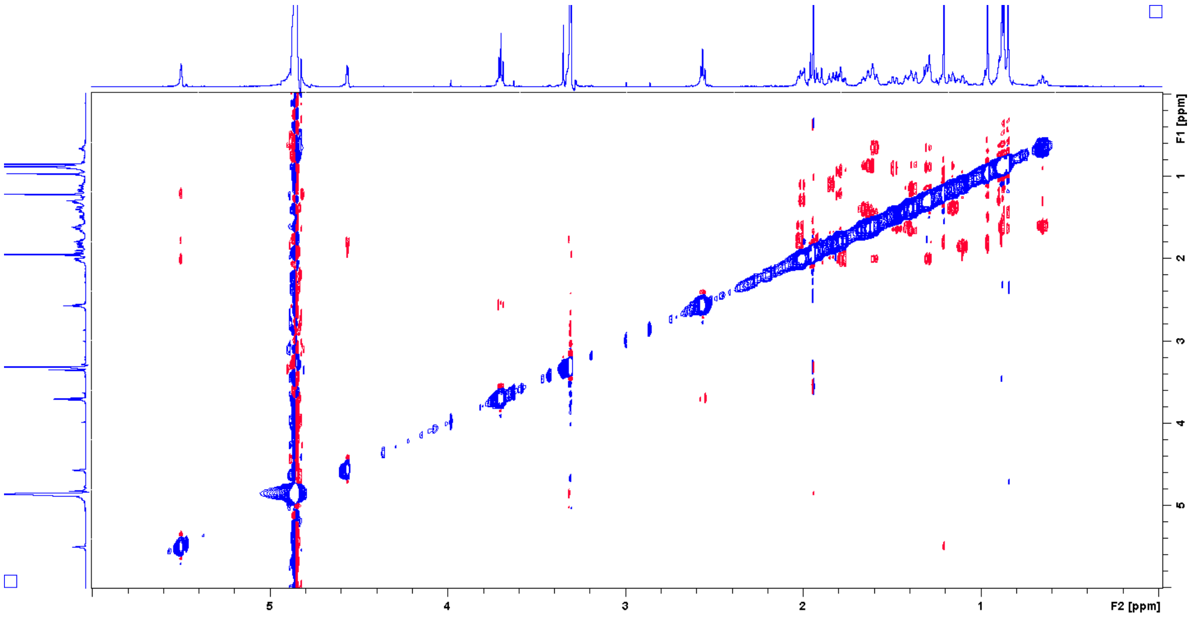

Figure S16. NOESY spectrum of scalimide B (**2**) in CD_3OD (600 MHz).

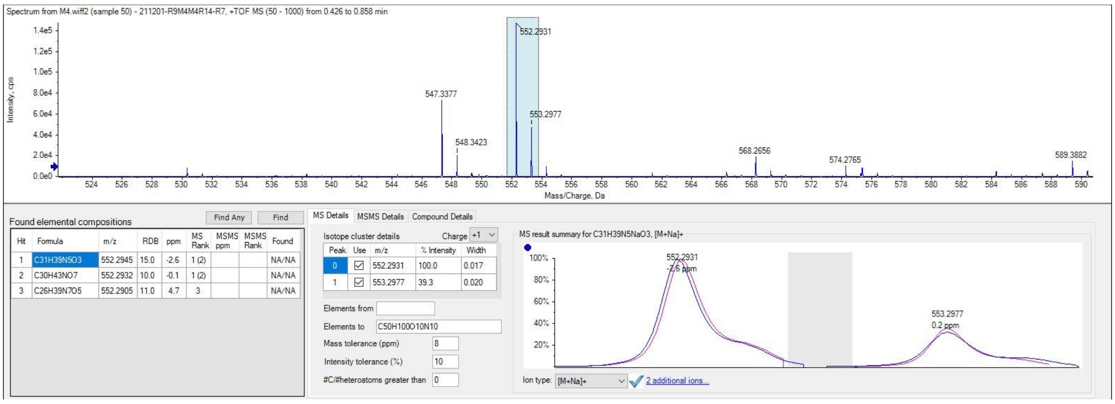

Figure S17. HRMS spectrum of scalimide B (**2**).

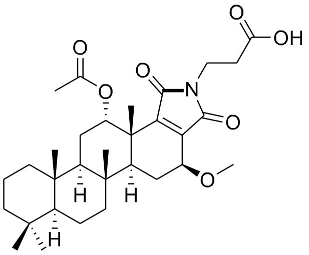

3

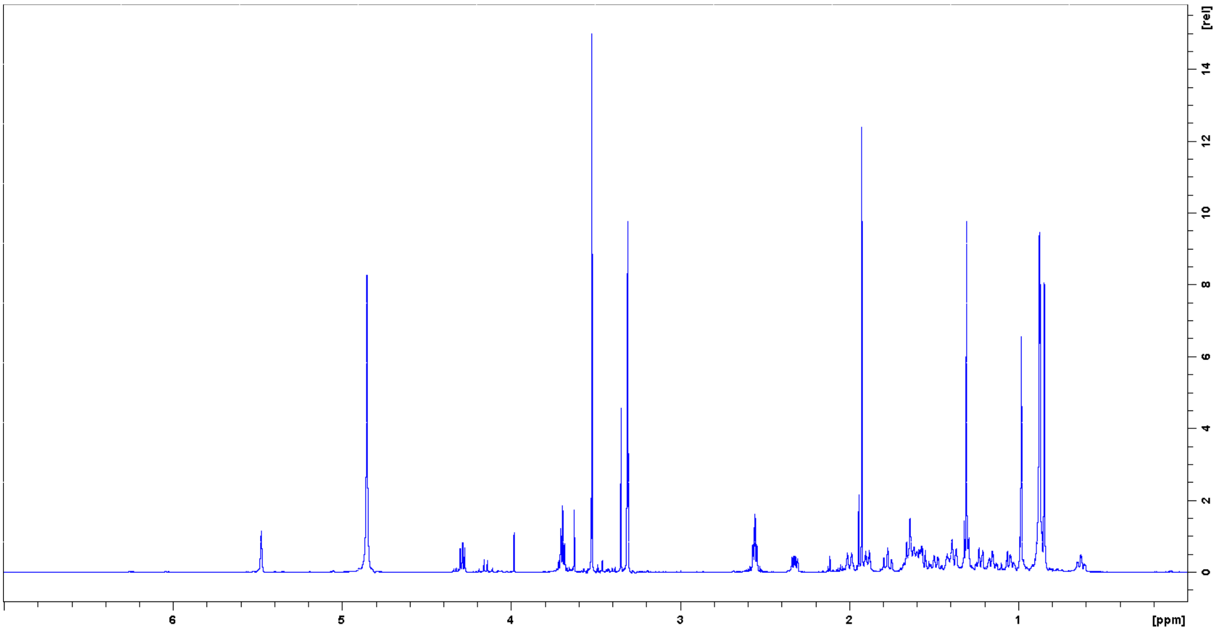

Figure S18. ^{1}H NMR spectrum of scalimide C (**3**) in CD_3OD (600 MHz).

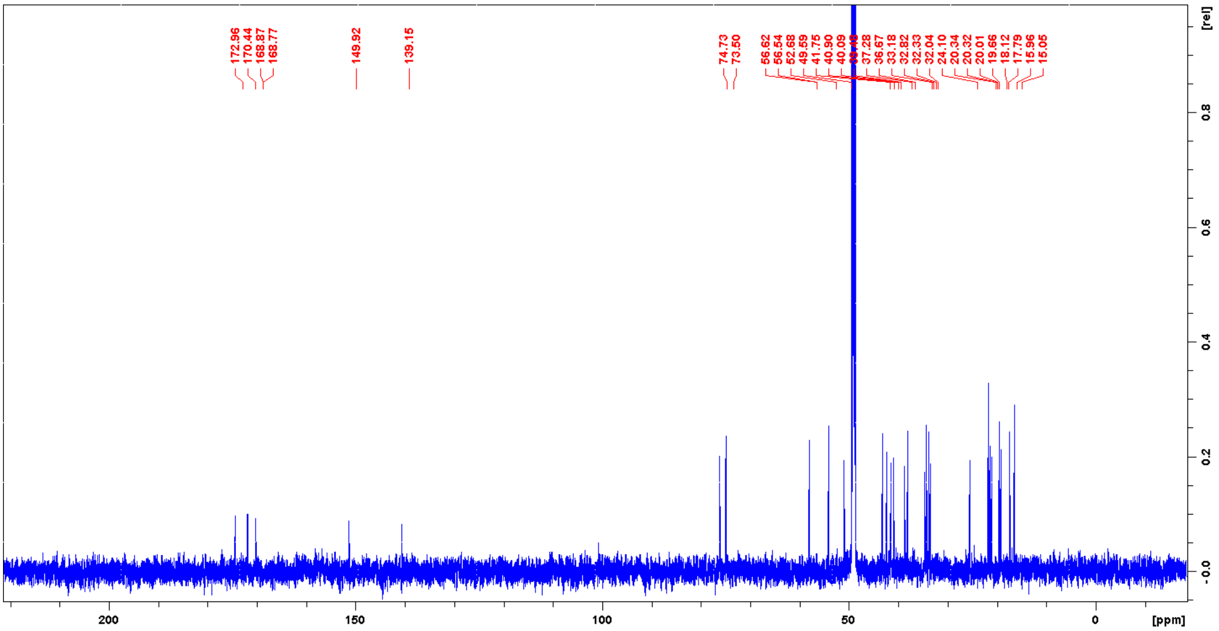

Figure S19. ^{13}C NMR spectrum of scalimide C (**3**) in CD_3OD (150 MHz).

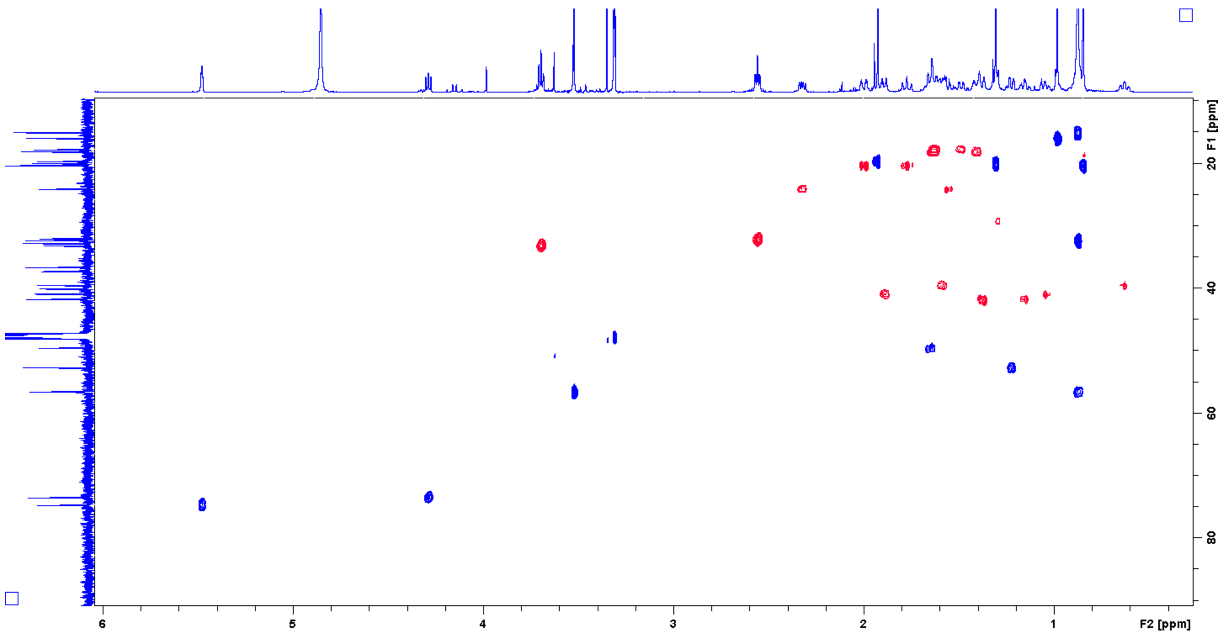

Figure S20. HSQC spectrum of scalimide C (**3**) in CD_3OD (600 MHz).

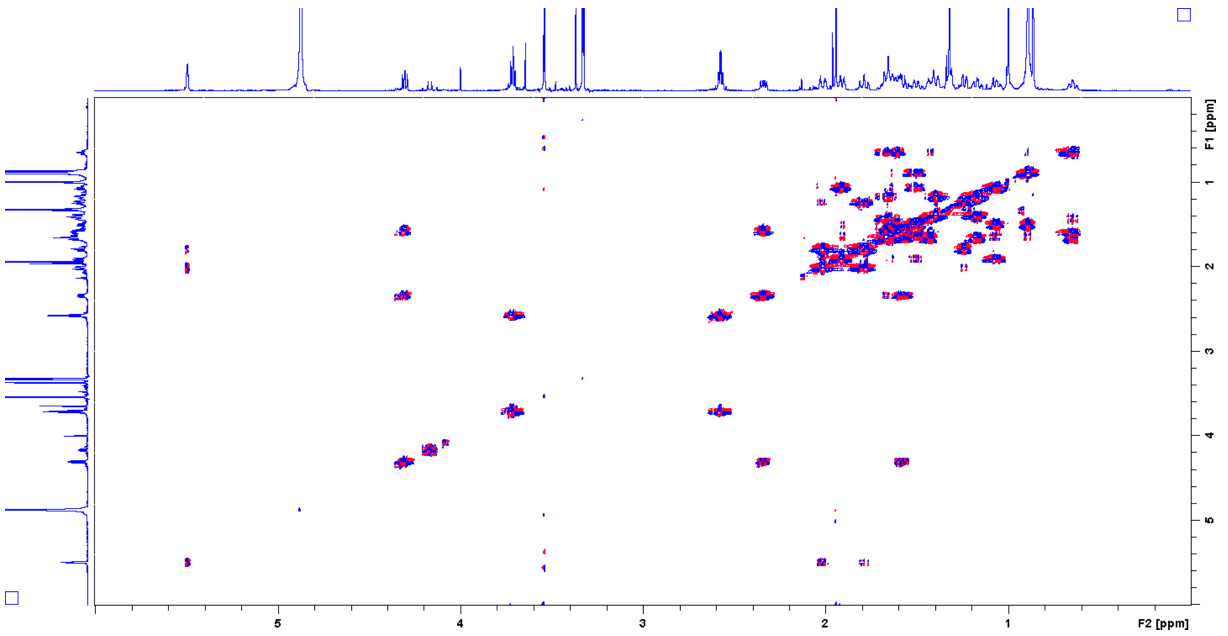

Figure S21. ^1H-^1H COSY spectrum of scalimide C (**3**) in CD_3OD (600 MHz).

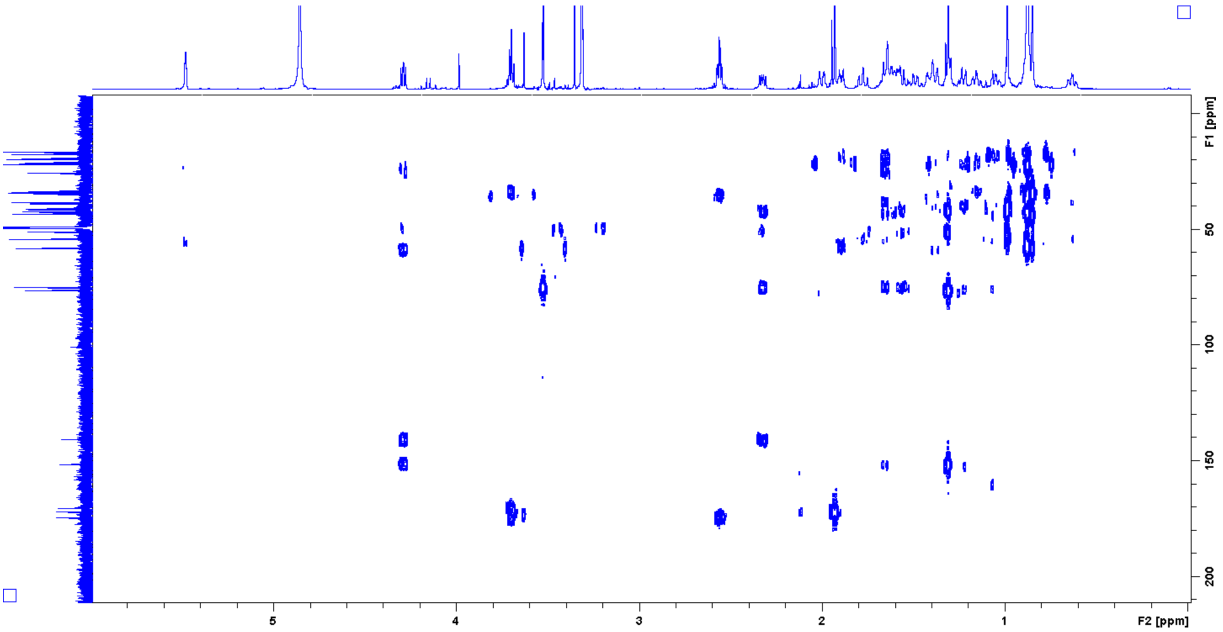

Figure S22. HMBC spectrum of scalimide C (**3**) in CD_3OD (600 MHz).

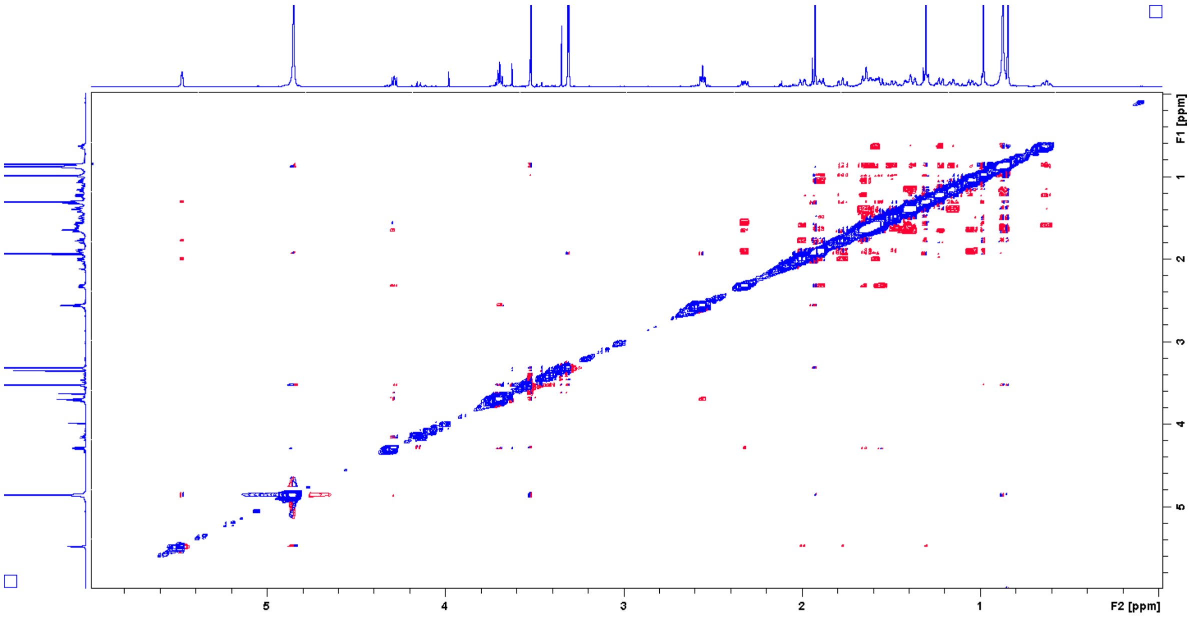

Figure S23. NOESY spectrum of scalimide C (**3**) in CD_3OD (600 MHz).

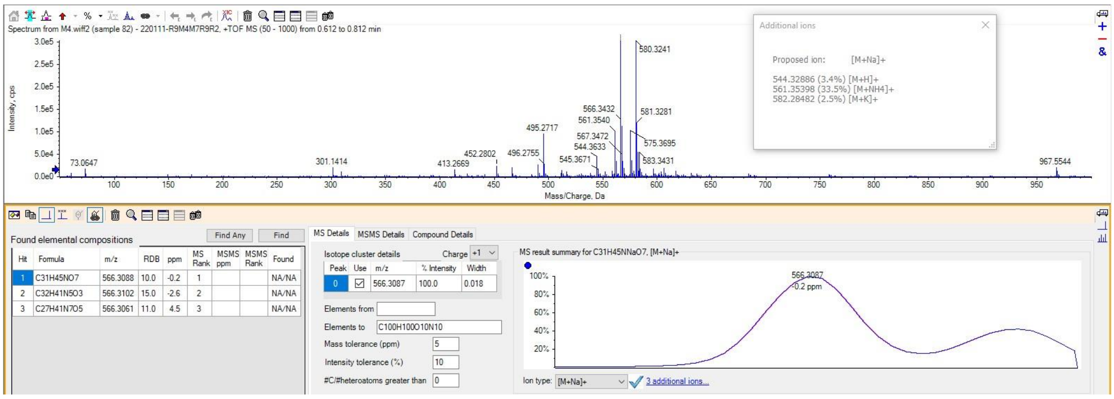

Figure S24. HRMS spectrum of scalimide C (**3**).

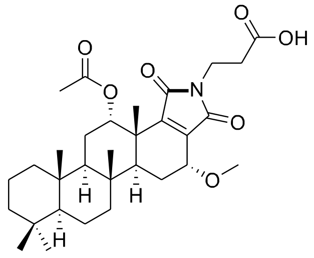

4

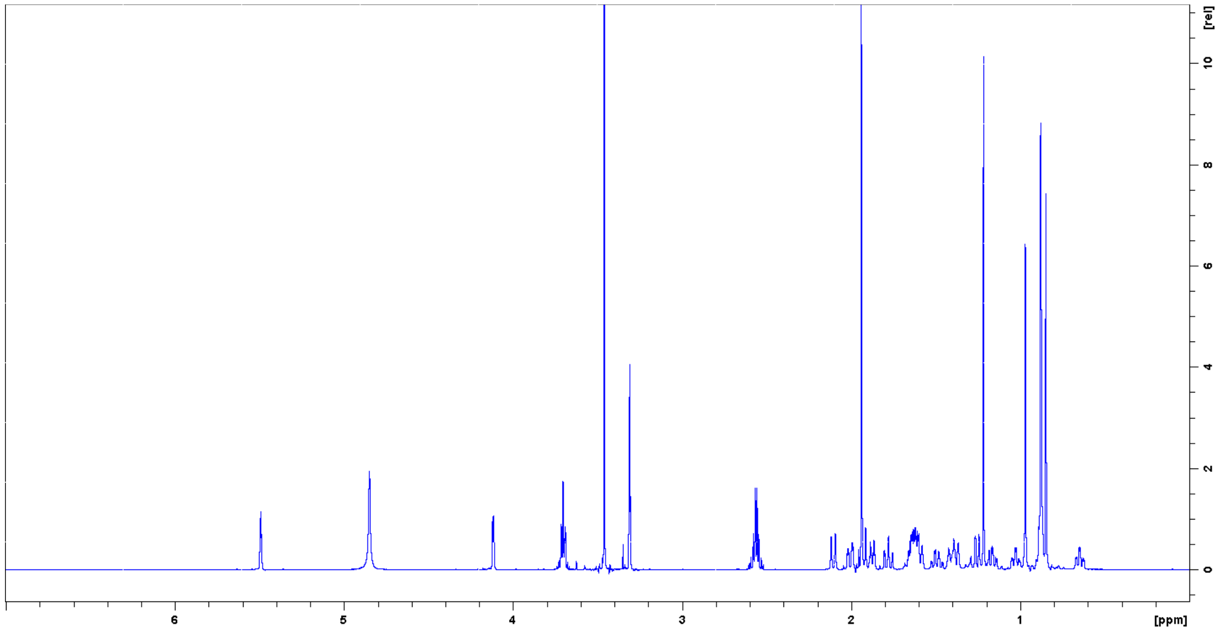

Figure S25. ^{1}H NMR spectrum of scalimide D (**4**) in CD_3OD (600 MHz).

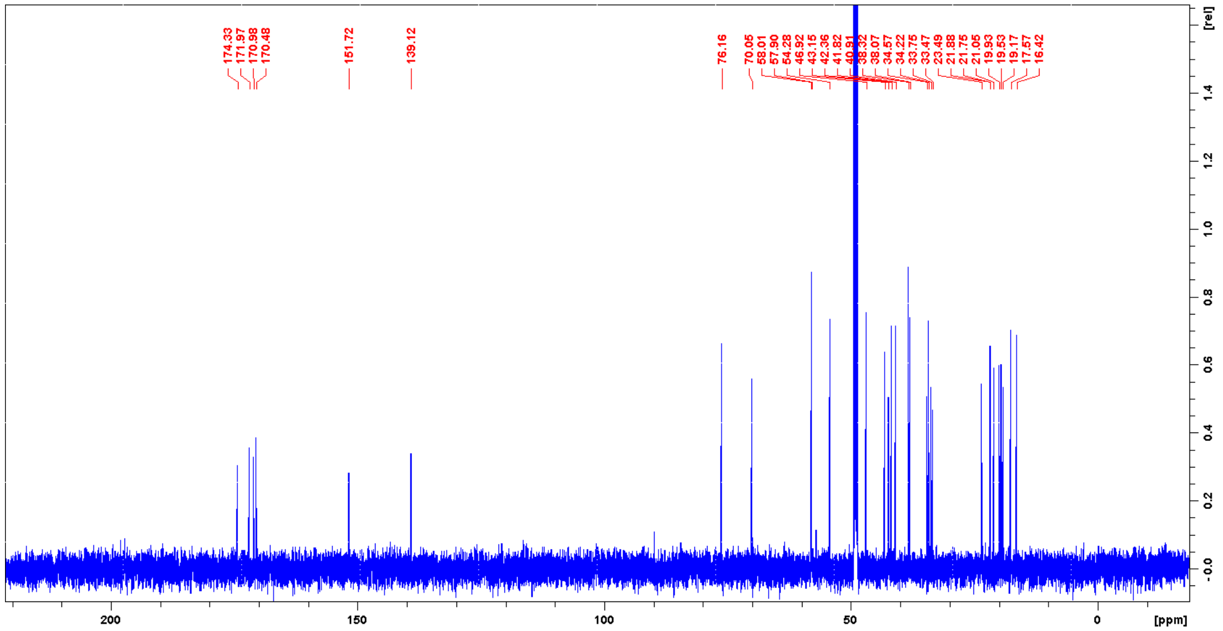

Figure S26. ^{13}C NMR spectrum of scalimide D (**4**) in CD_3OD (150 MHz).

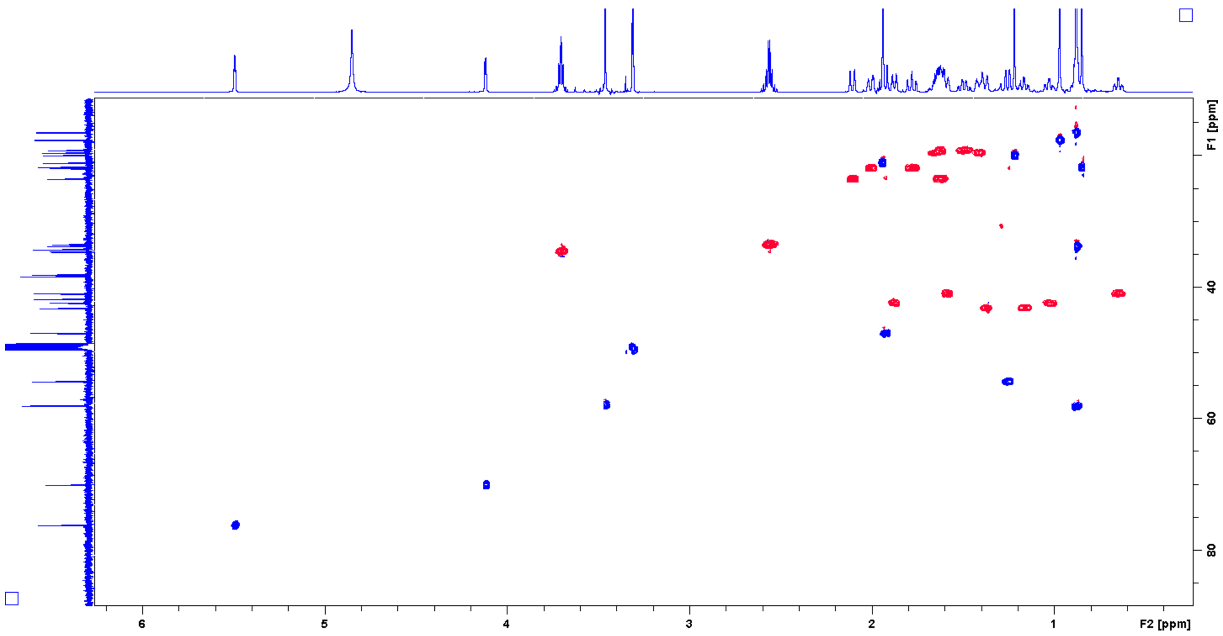

Figure S27. HSQC spectrum of scalimide D (**4**) in CD_3OD (600 MHz).

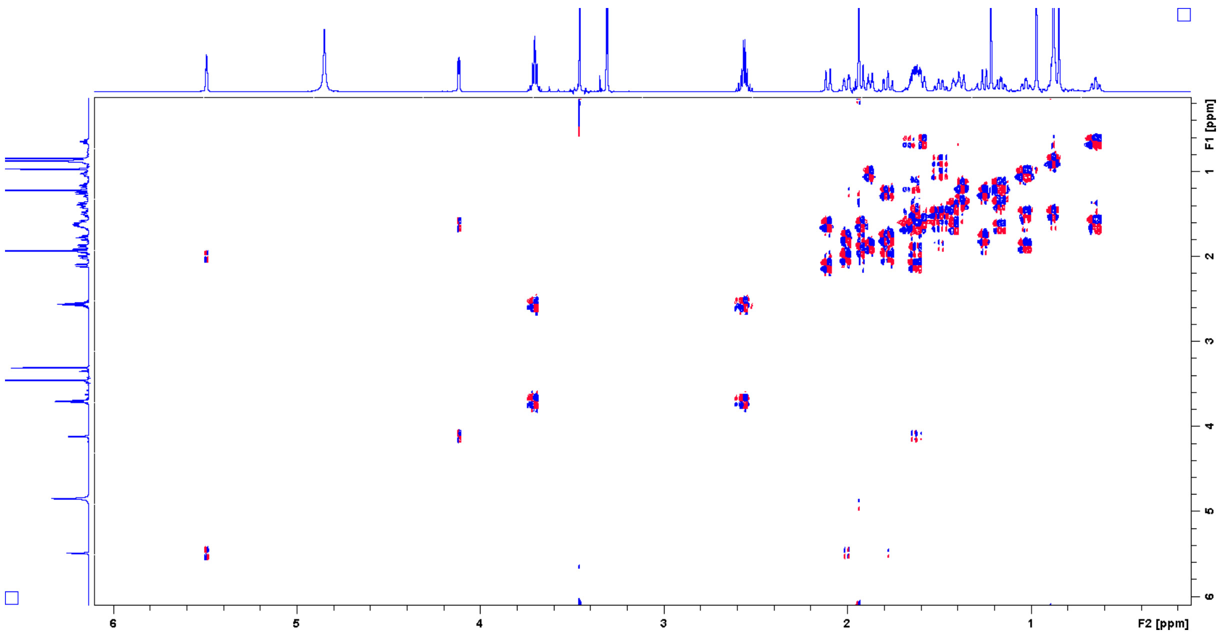

Figure S28. ^1H-^1H COSY spectrum of scalimide D (**4**) in CD_3OD (600 MHz).

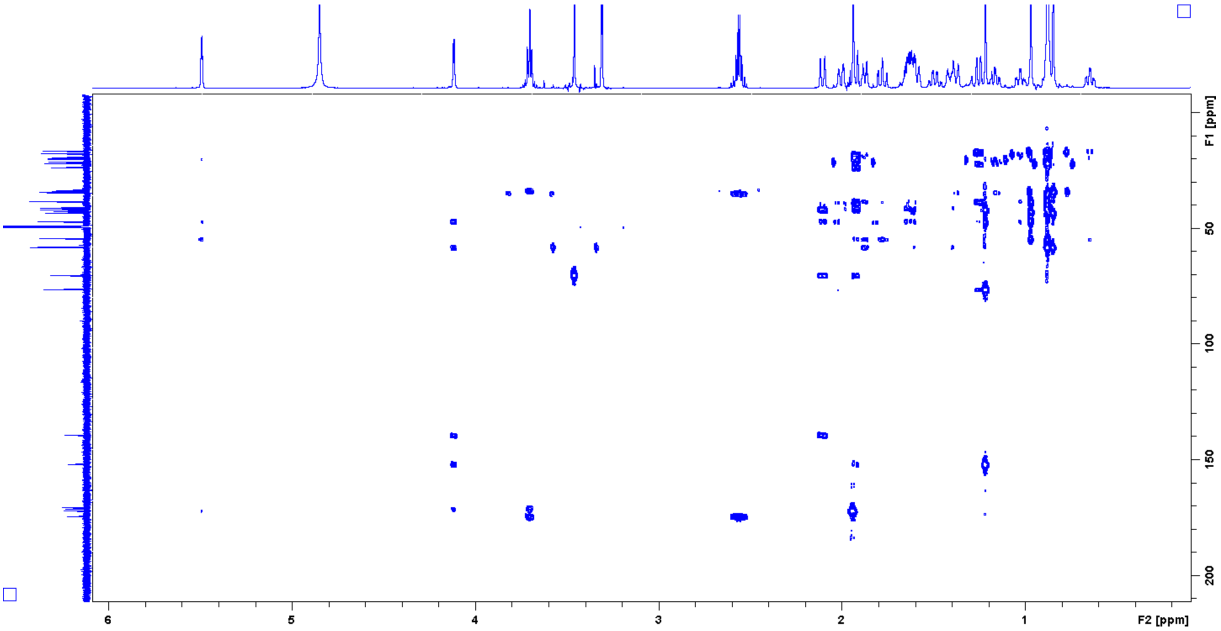

Figure S29. HMBC spectrum of scalimide D (**4**) in CD_3OD (600 MHz).

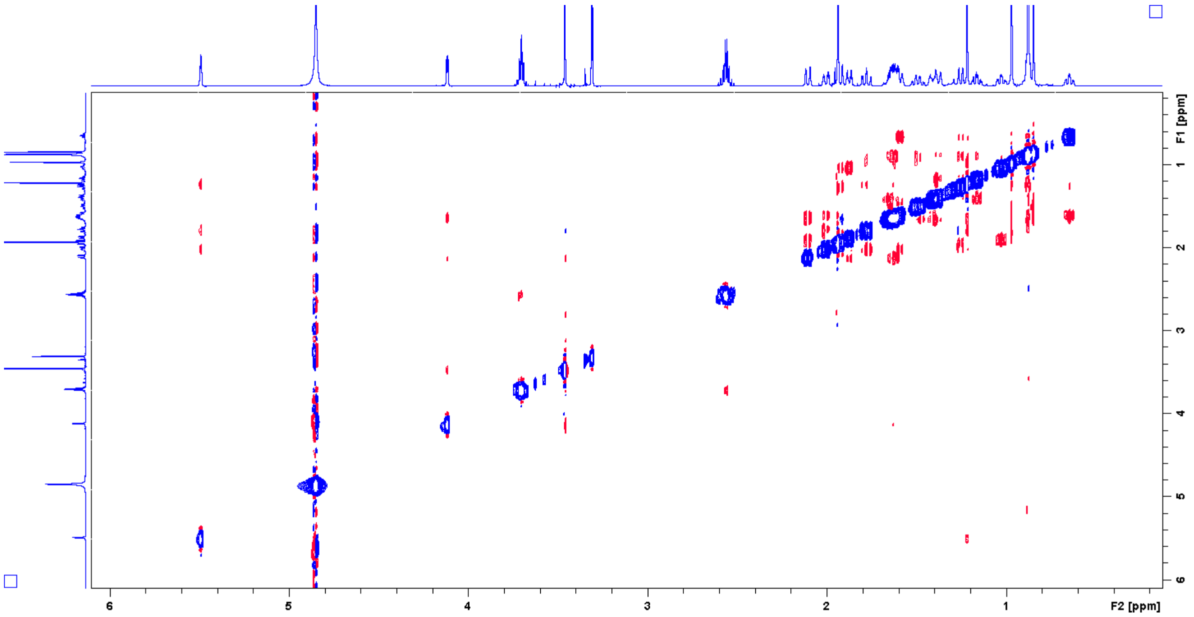

Figure S30. NOESY spectrum of scalimide D (**4**) in CD_3OD (600 MHz).

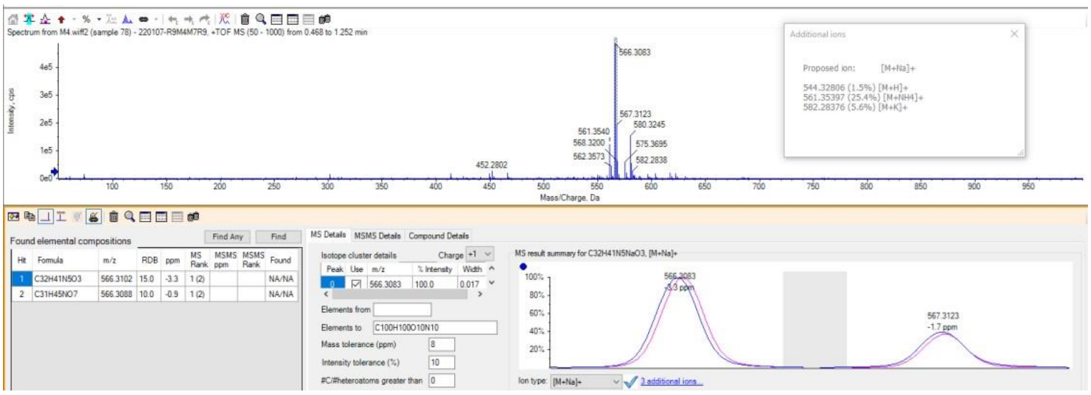

Figure S31. HRMS spectrum of scalimide D (**4**).

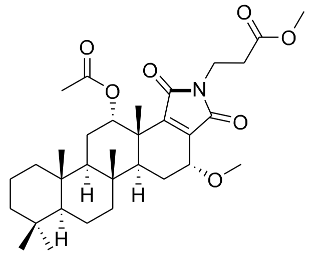

5

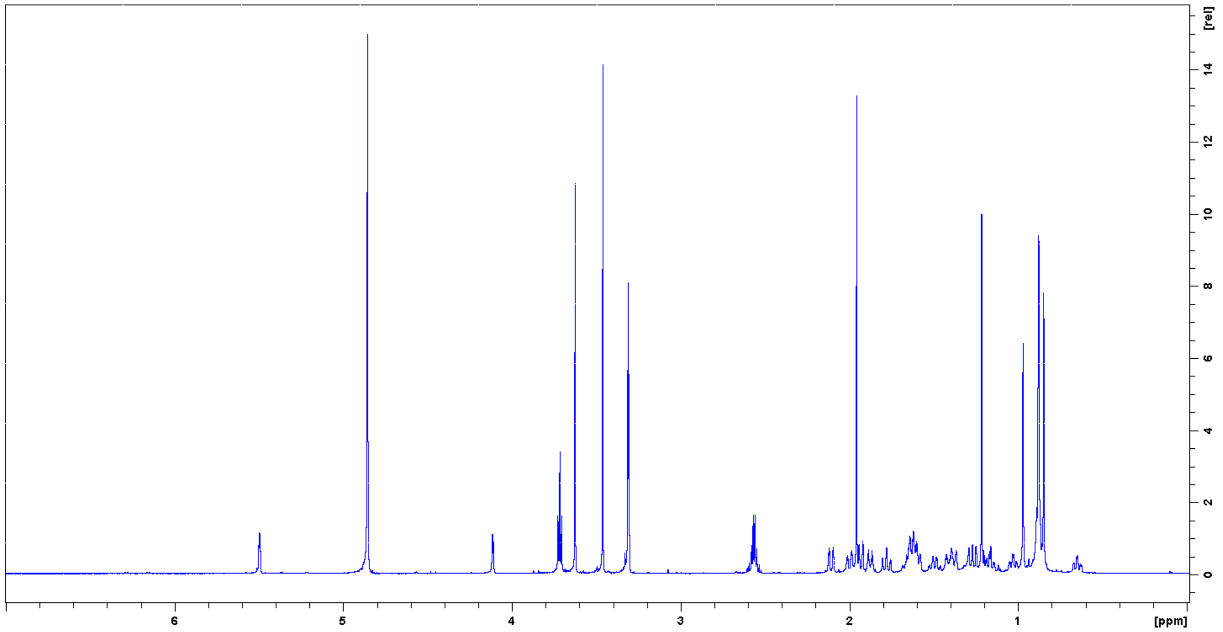

Figure S32. ^{1}H NMR spectrum of scalimide E (**5**) in CD_3OD (600 MHz).

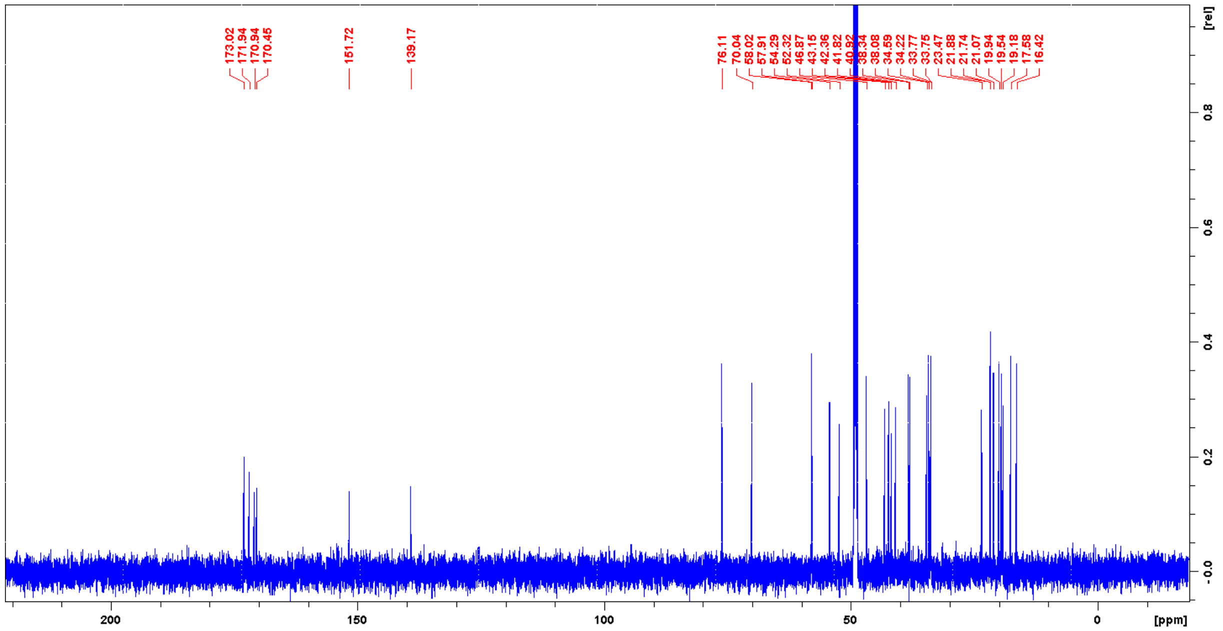

Figure S33. ^{13}C NMR spectrum of scalimide E (**5**) in CD_3OD (150 MHz).

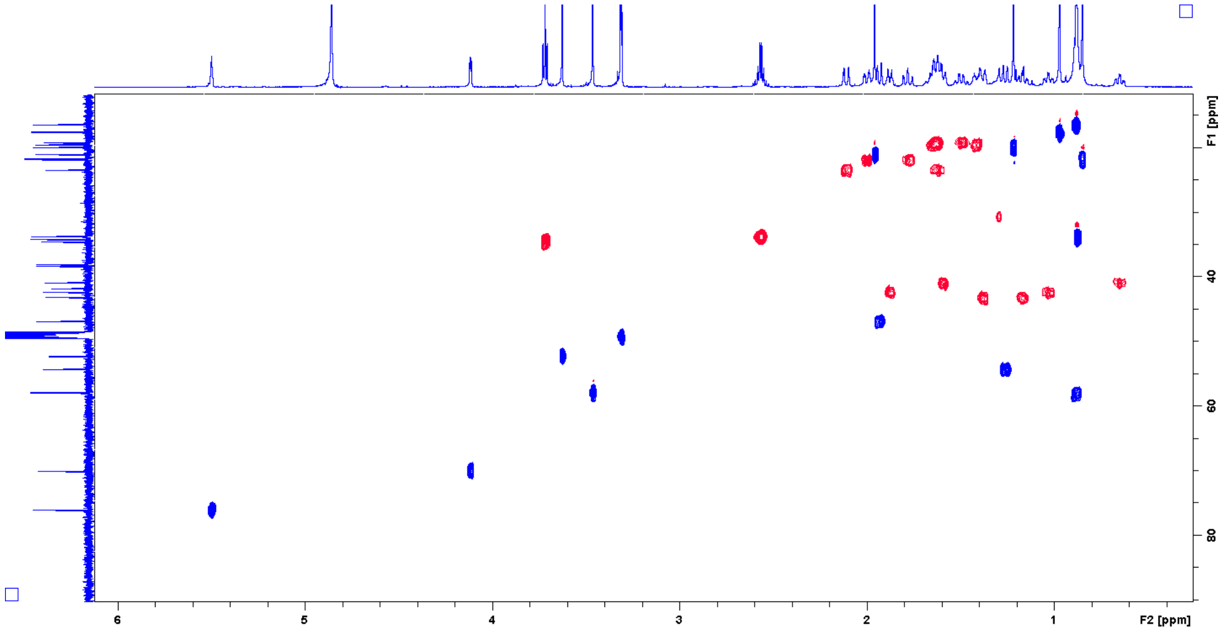

Figure S34. HSQC spectrum of scalimide E (**5**) in CD_3OD (600 MHz).

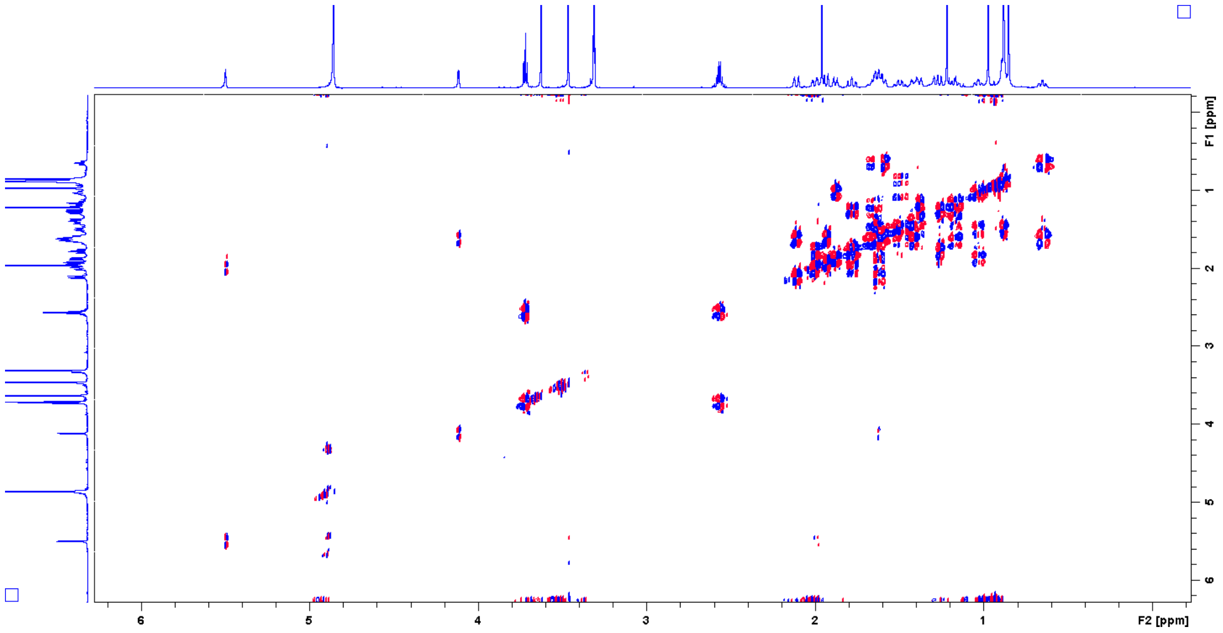

Figure S35. ^{1}H-^{1}H COSY spectrum of scalimide E (**5**) in CD_3OD (600 MHz).

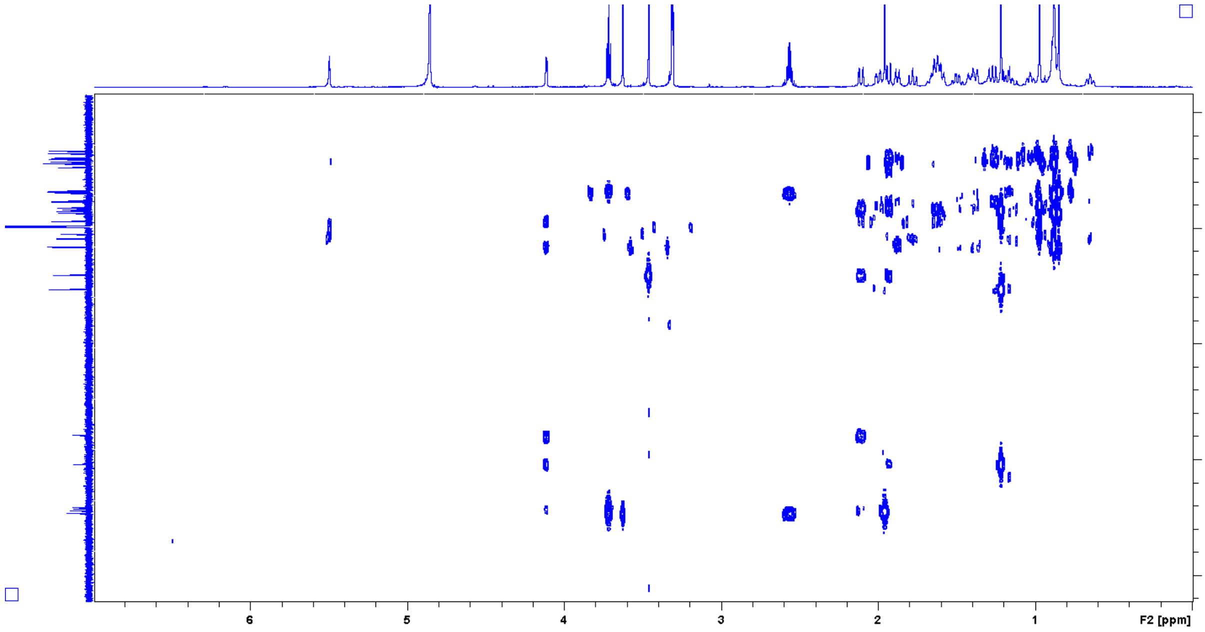

Figure S36. HMBC spectrum of scalimide E (**5**) in CD_3OD (600 MHz).

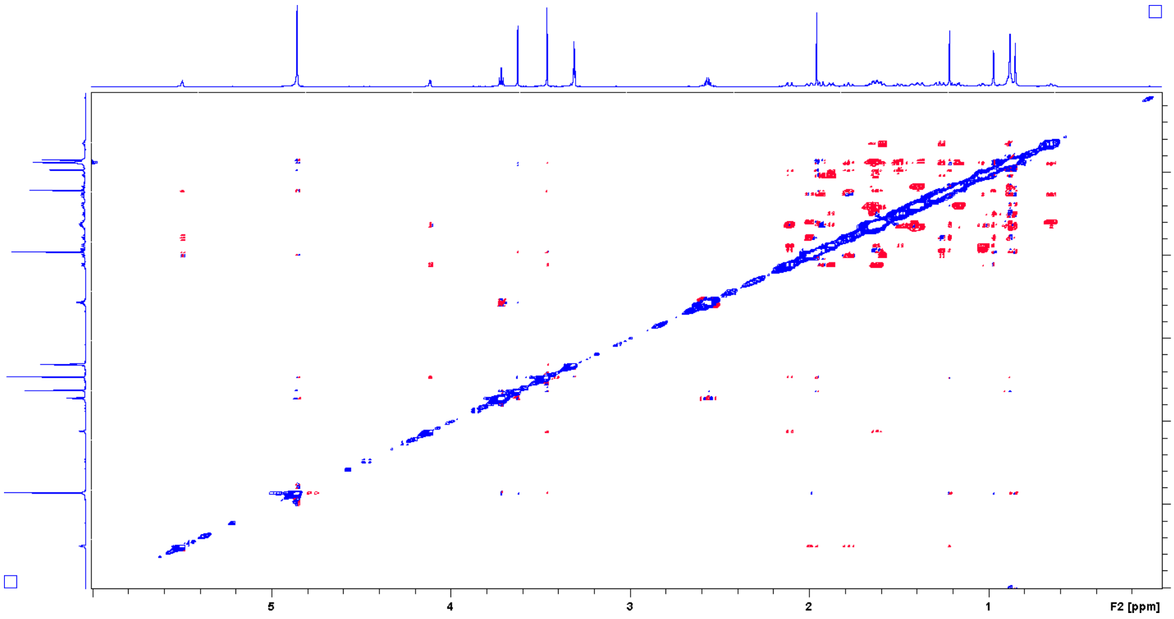

Figure S37. NOESY spectrum of scalimide E (**5**) in CD_3OD (600 MHz).

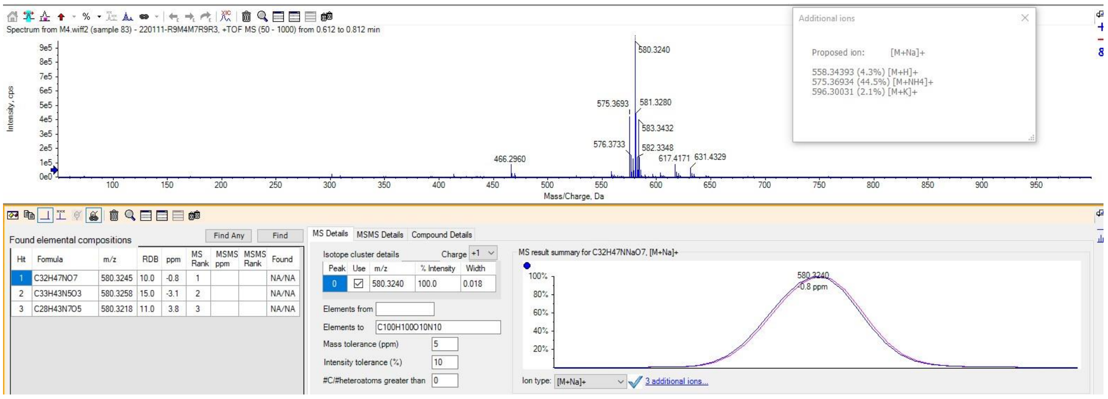

Figure S38. HRMS spectrum of scalimide E (**5**).

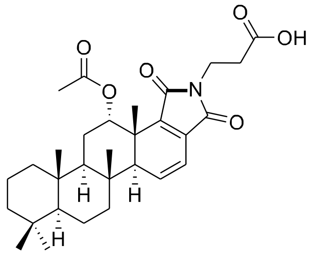

6

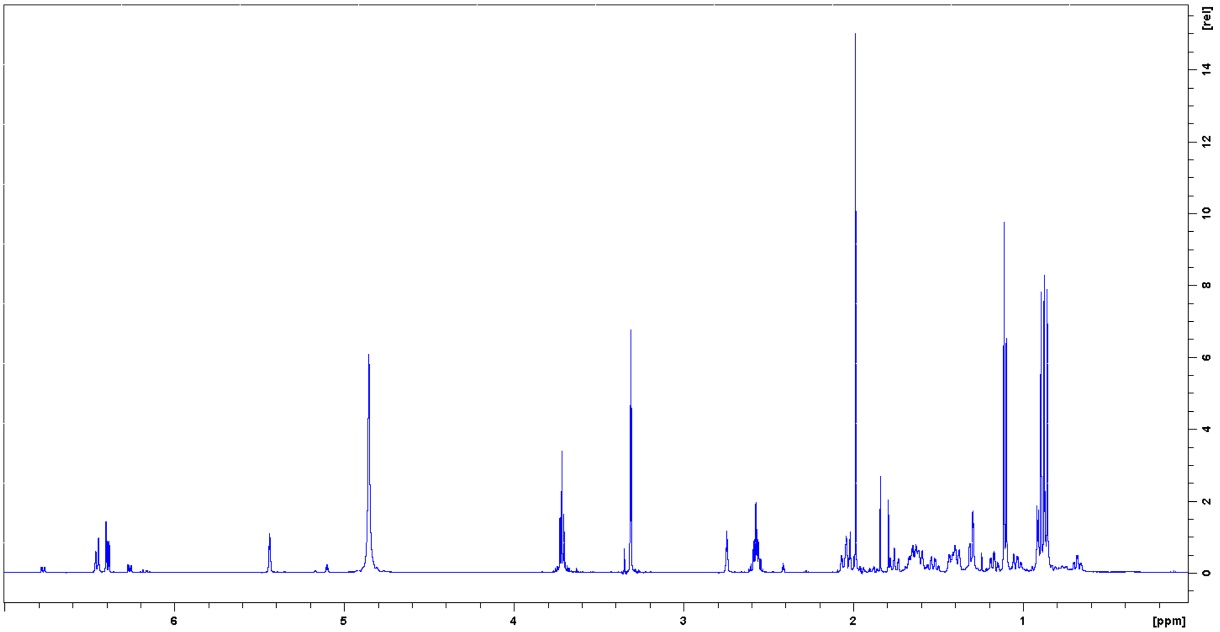

Figure S39. ^{1}H NMR spectrum of scalimide F (**6**) in CD_3OD (600 MHz).

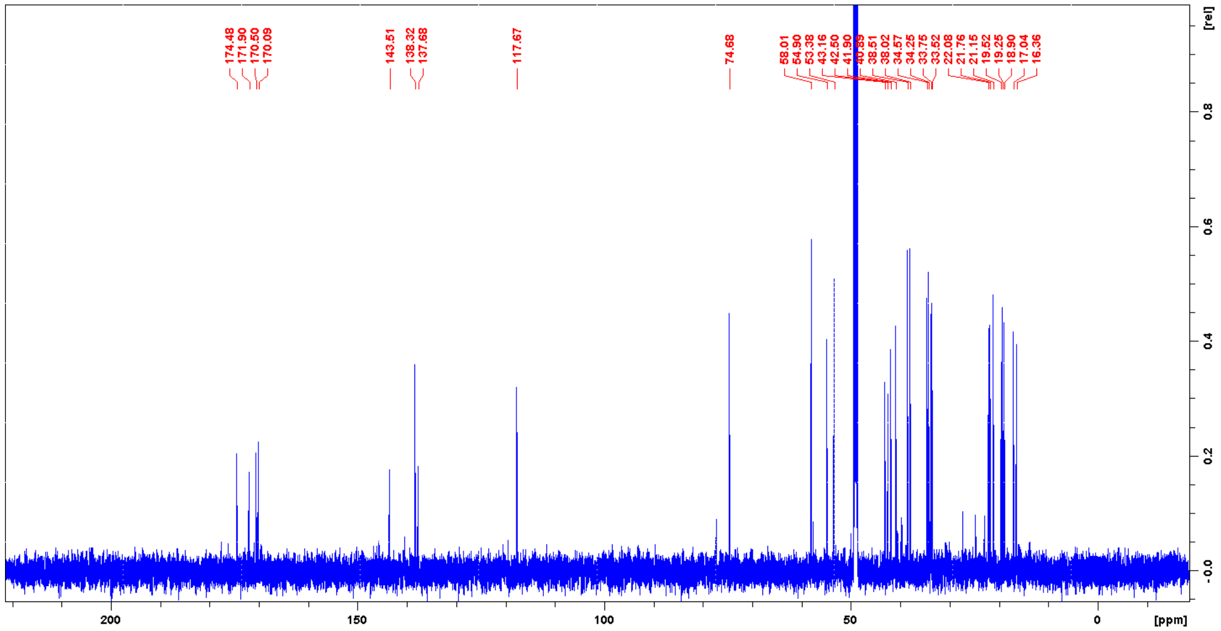

Figure S40. ^{13}C NMR spectrum of scalimide F (**6**) in CD_3OD (150 MHz).

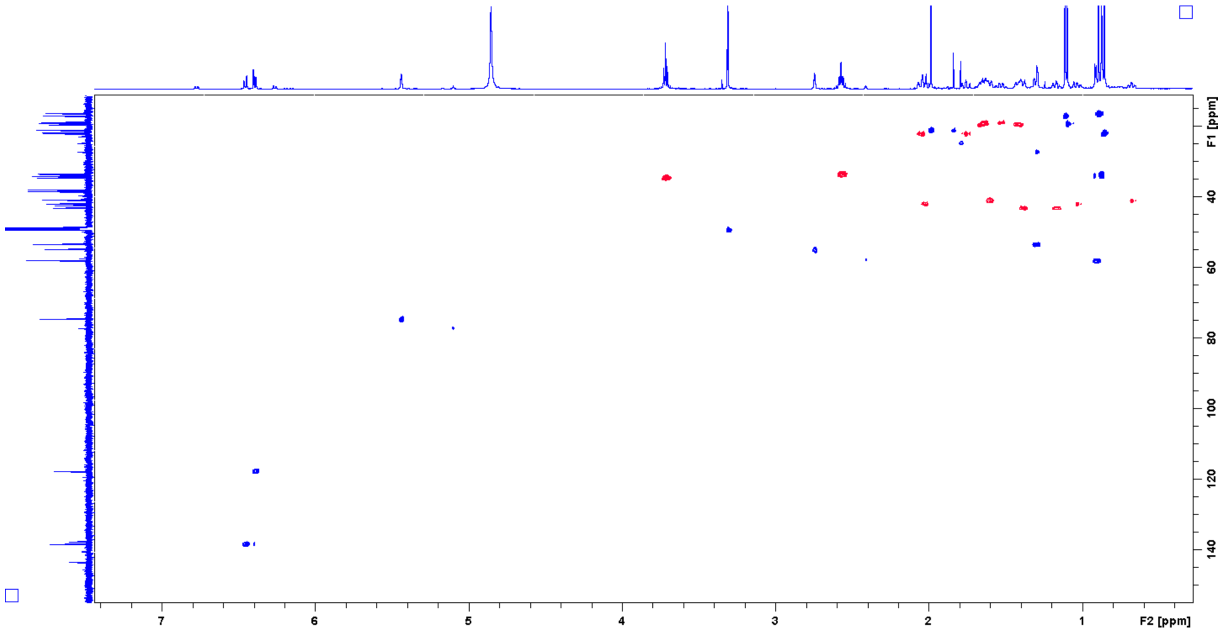

Figure S41. HSQC spectrum of scalimide F (**6**) in CD_3OD (600 MHz).

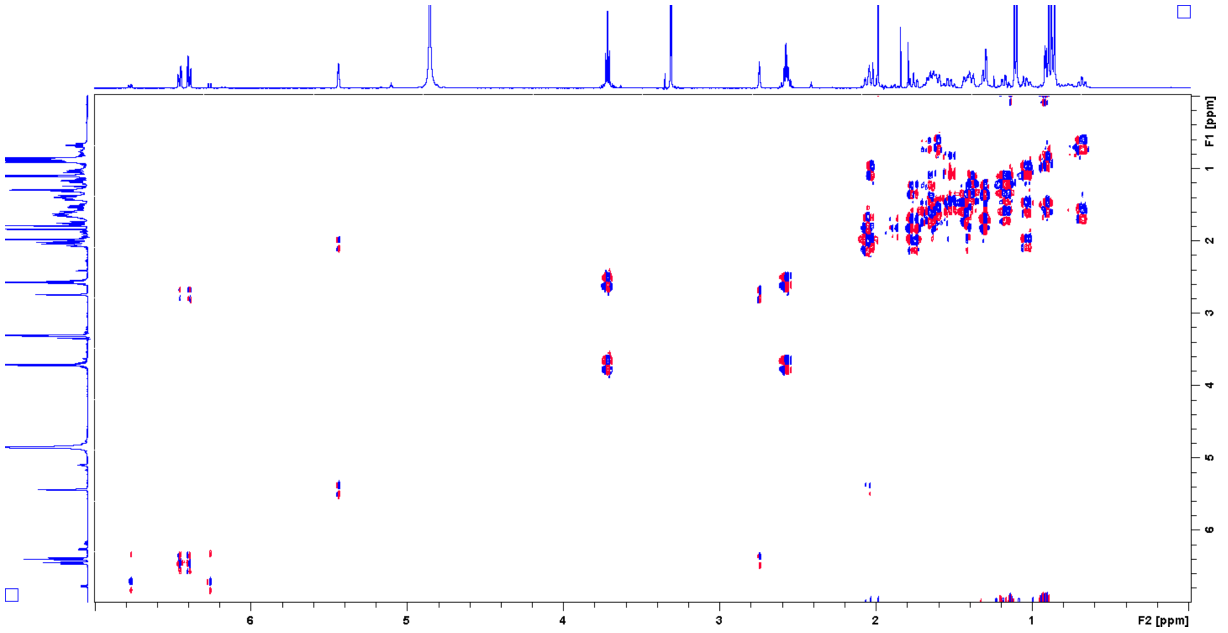

Figure S42. ^1H-^1H COSY spectrum of scalimide F (**6**) in CD_3OD (600 MHz).

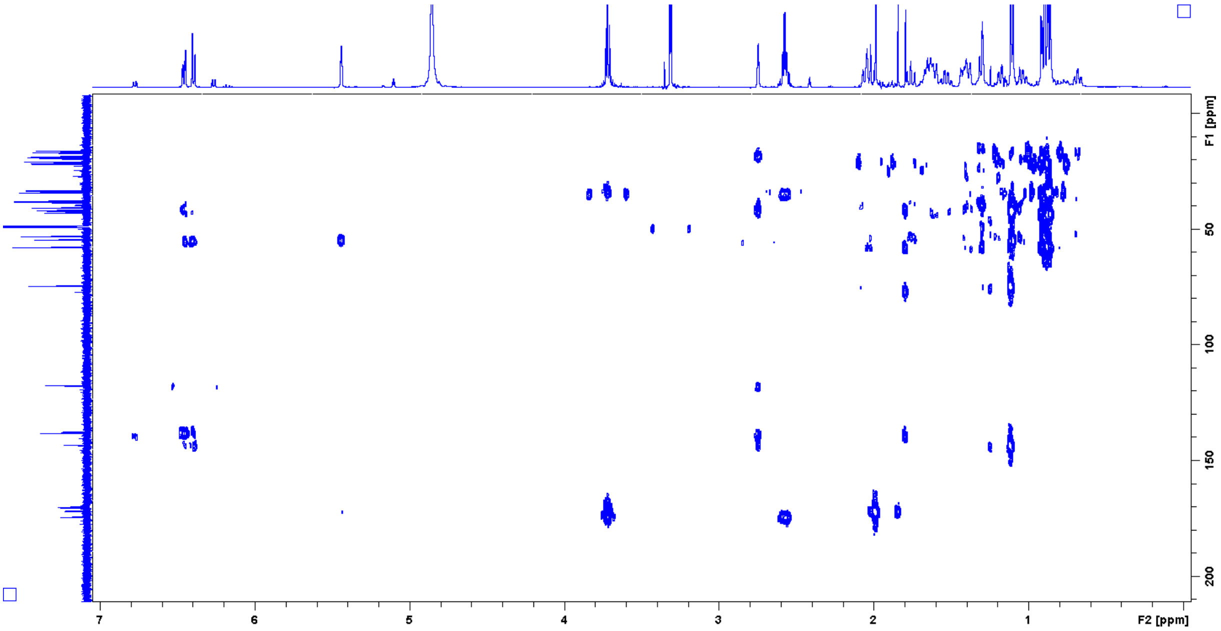

Figure S43. HMBC spectrum of scalimide F (**6**) in CD_3OD (600 MHz).

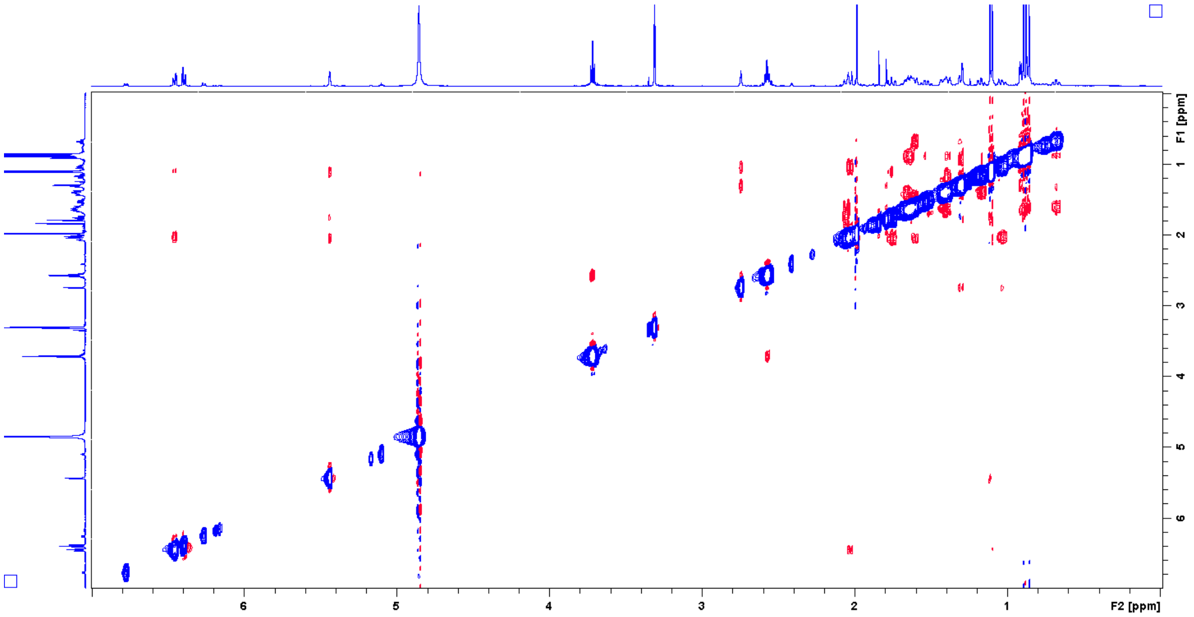

Figure S44. NOESY spectrum of scalimide F (**6**) in CD_3OD (600 MHz).

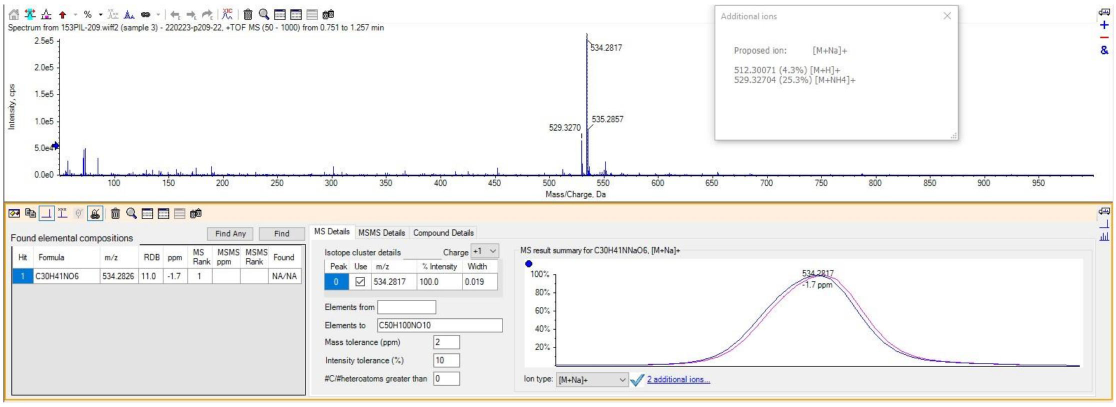

Figure S45. HRMS spectrum of scalimide F (**6**).

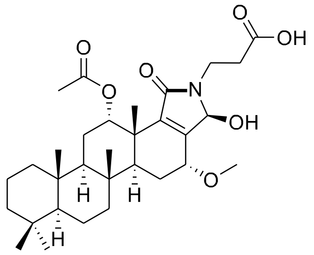

7

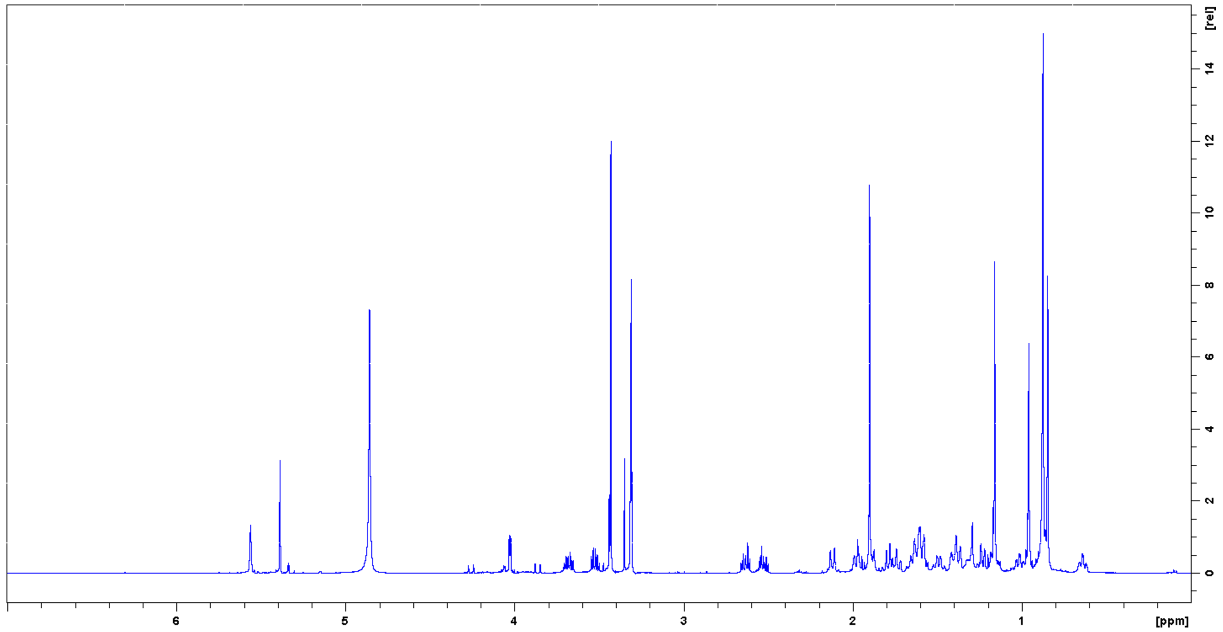

Figure S46. ^{1}H NMR spectrum of scalimide G (**7**) in CD_3OD (600 MHz).

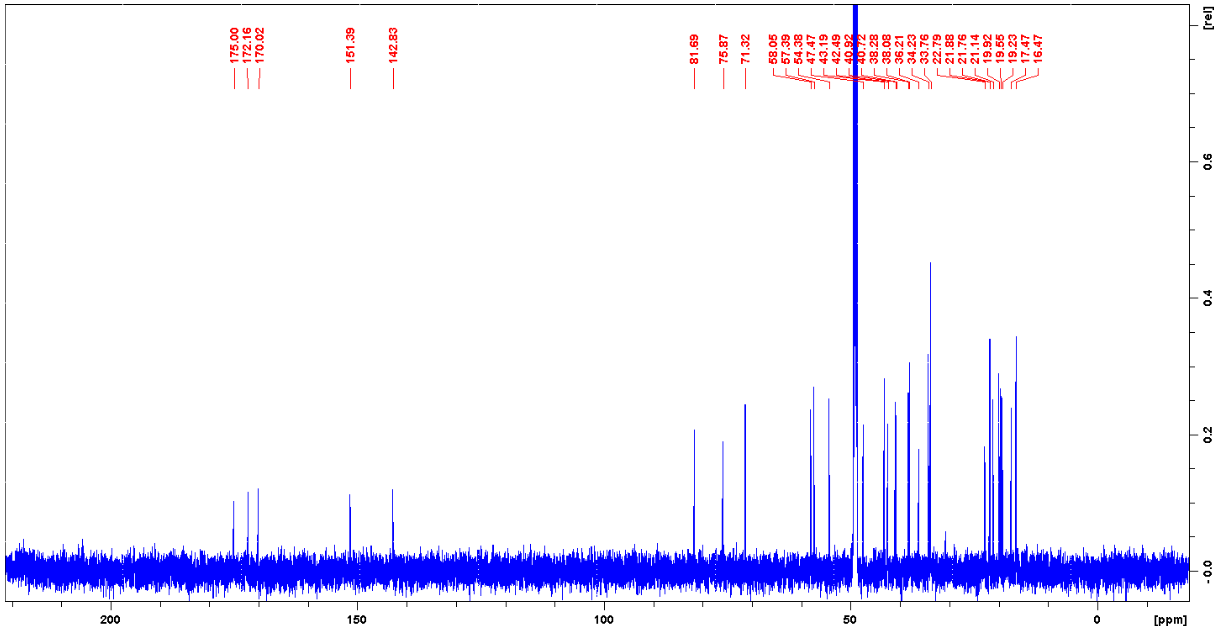

Figure S47. ^{13}C NMR spectrum of scalimide G (**7**) in CD_3OD (150 MHz).

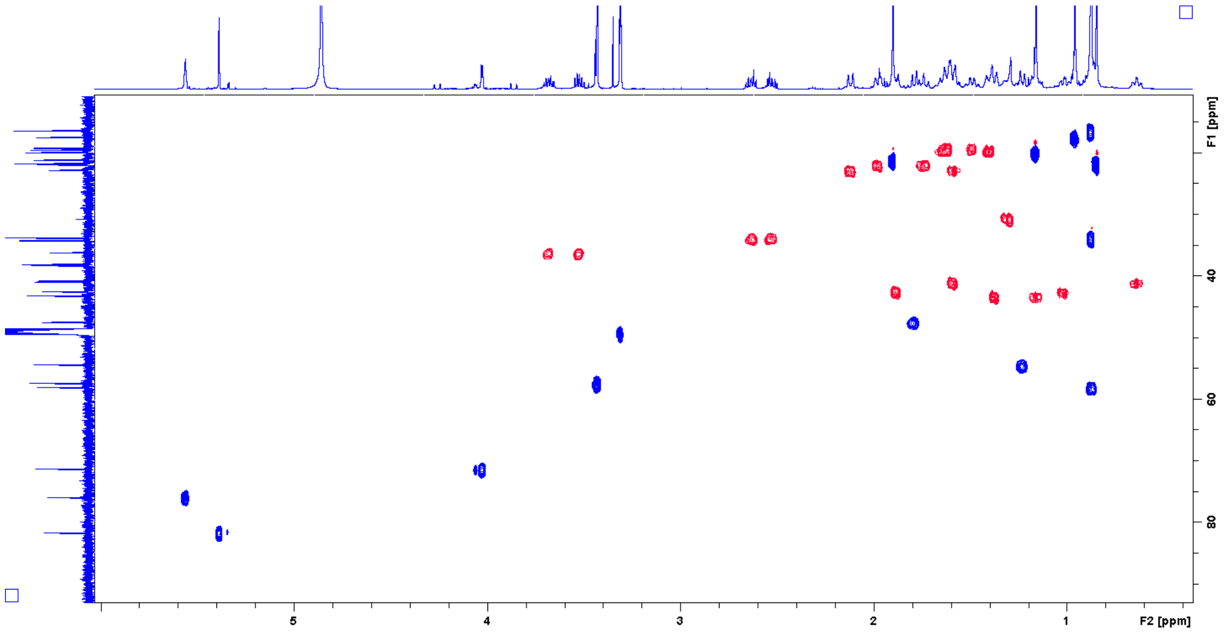

Figure S48. HSQC spectrum of scalimide G (**7**) in CD_3OD (600 MHz).

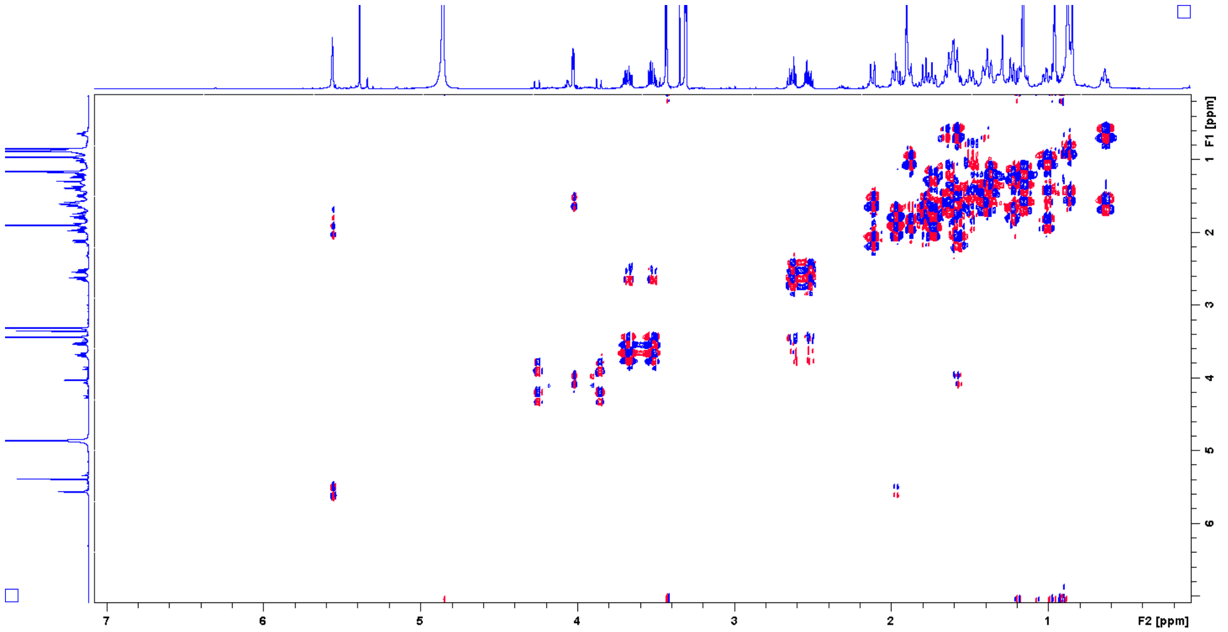

Figure S49. ^{1}H-^{1}H COSY spectrum of scalimide G (**7**) in CD_3OD (600 MHz).

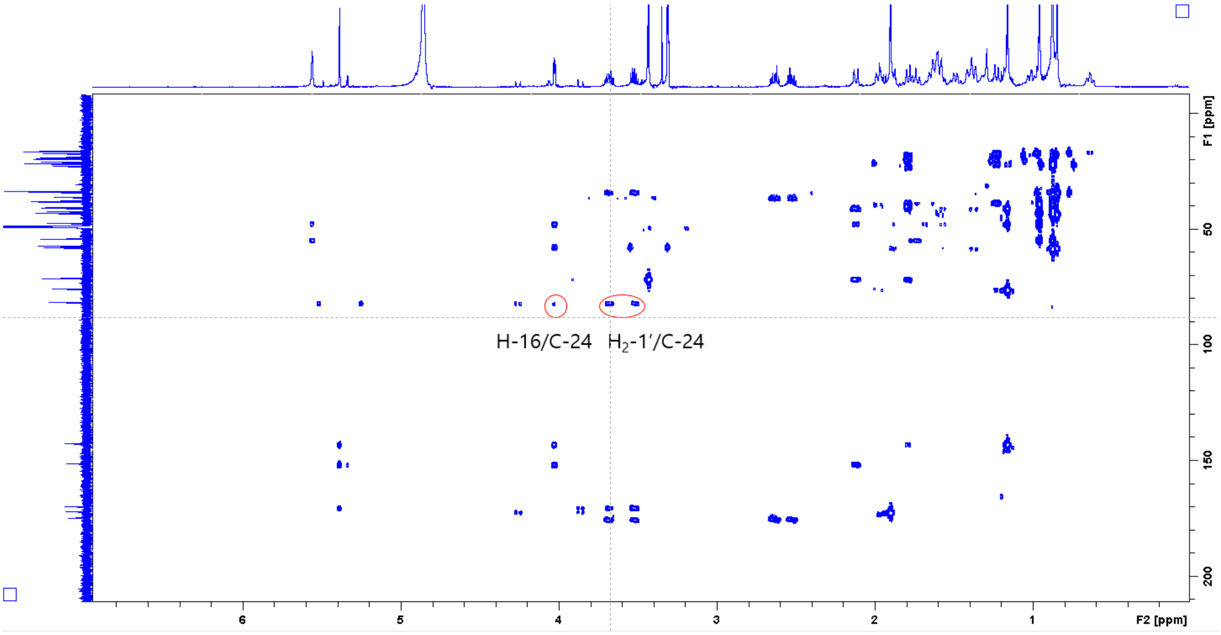

Figure S50. HMBC spectrum of scalimide G (**7**) in CD_3OD (600 MHz).

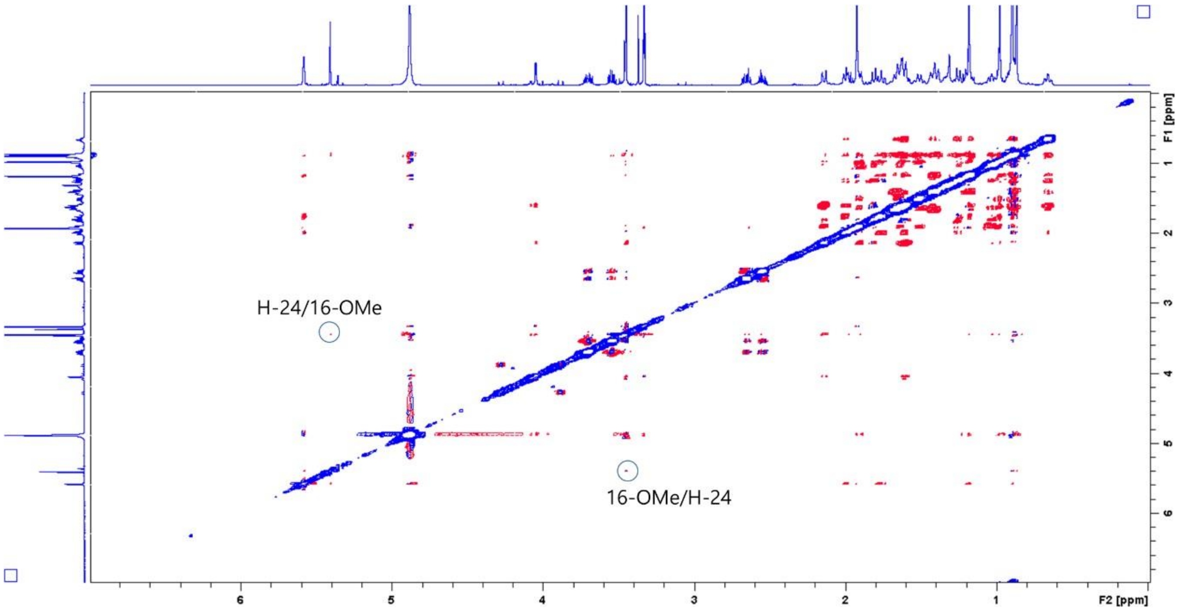

Figure S51. NOESY spectrum of scalimide G (**7**) in CD_3OD (600 MHz).

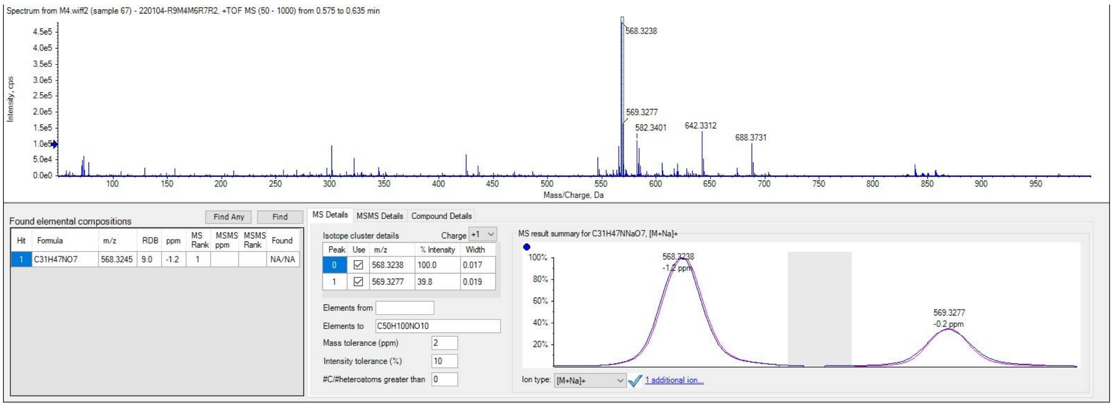

Figure S52. HRMS spectrum of scalimide G (**7**).

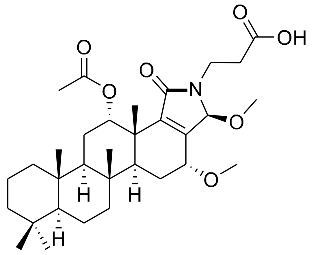

8

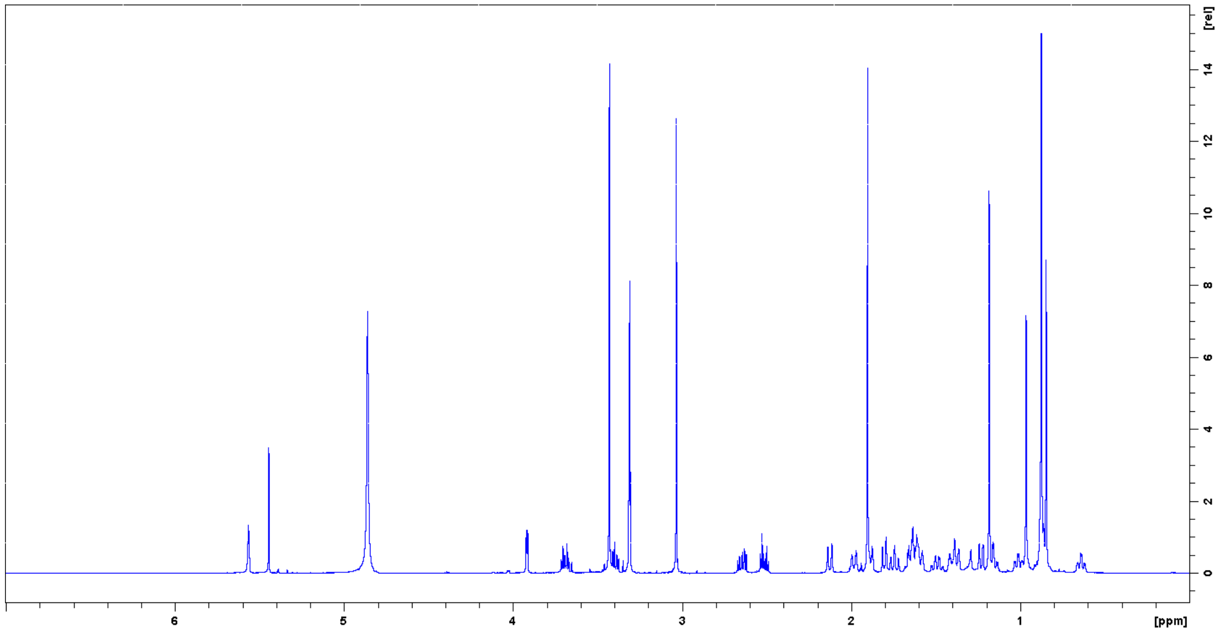

Figure S53. ^{1}H NMR spectrum of scalimide H (**8**) in CD_3OD (600 MHz).

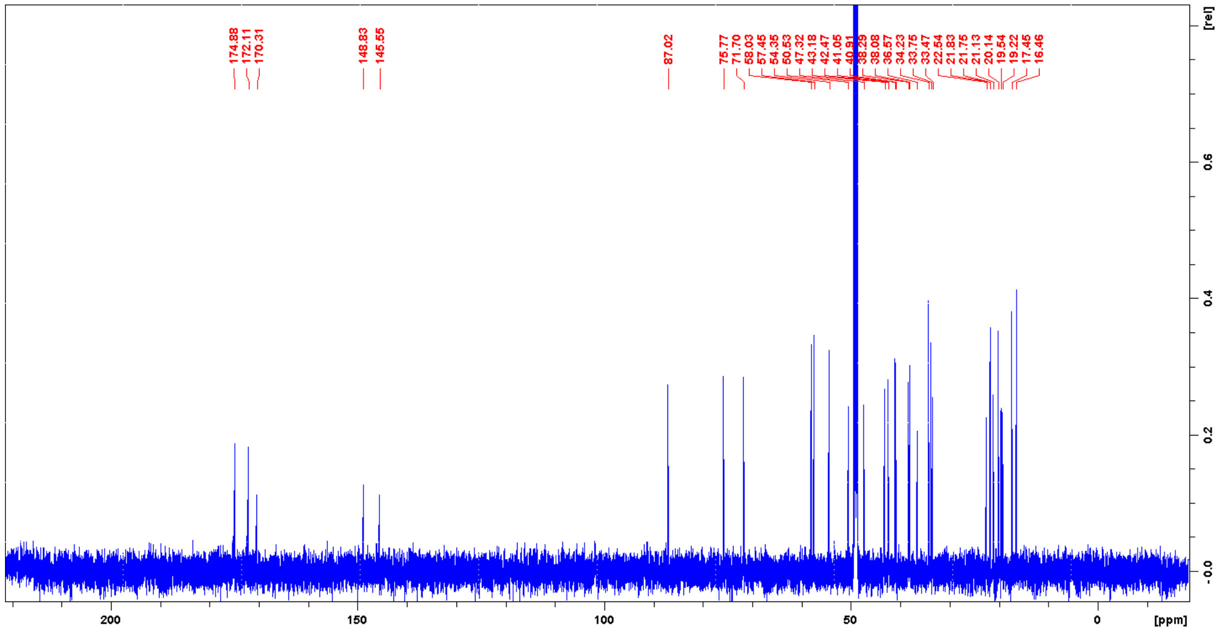

Figure S54. ^{13}C NMR spectrum of scalimide H (**8**) in CD_3OD (150 MHz).

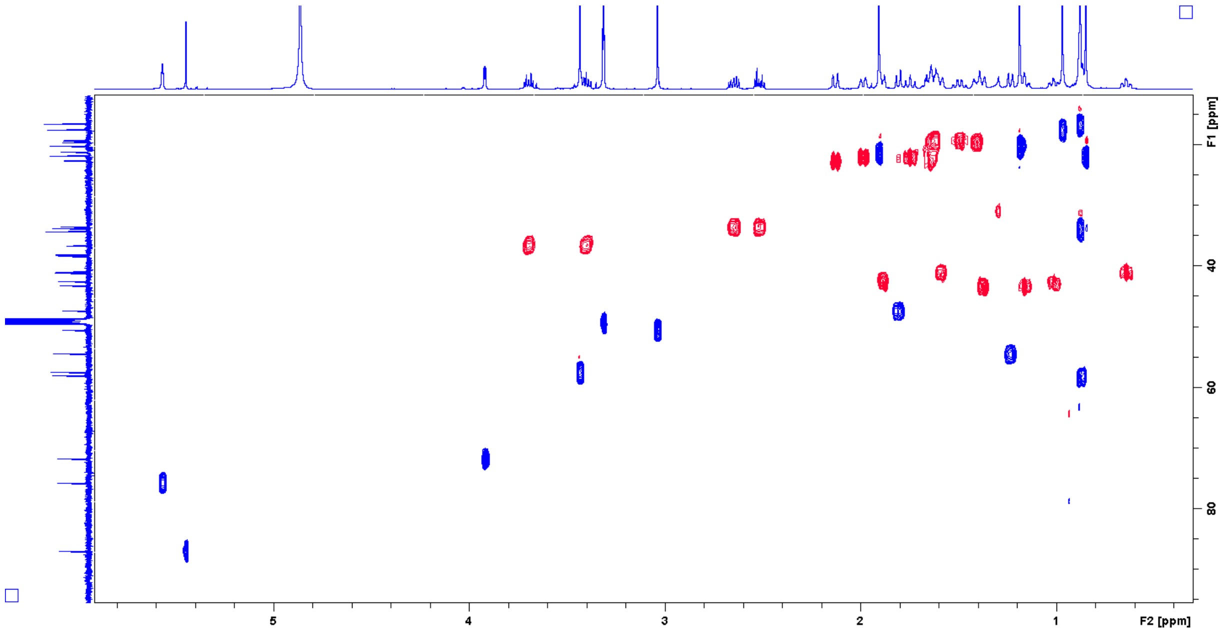

Figure S55. HSQC spectrum of scalimide H (**8**) in CD_3OD (600 MHz).

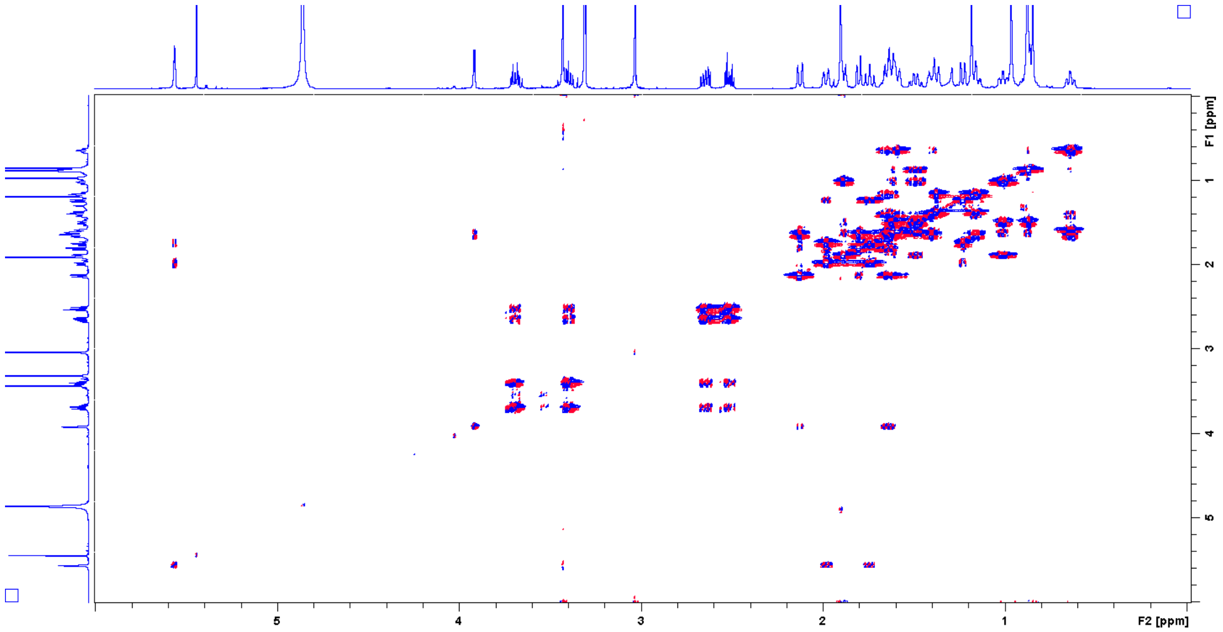

Figure S56. ^1H-^1H COSY spectrum of scalimide H (**8**) in CD_3OD (600 MHz).

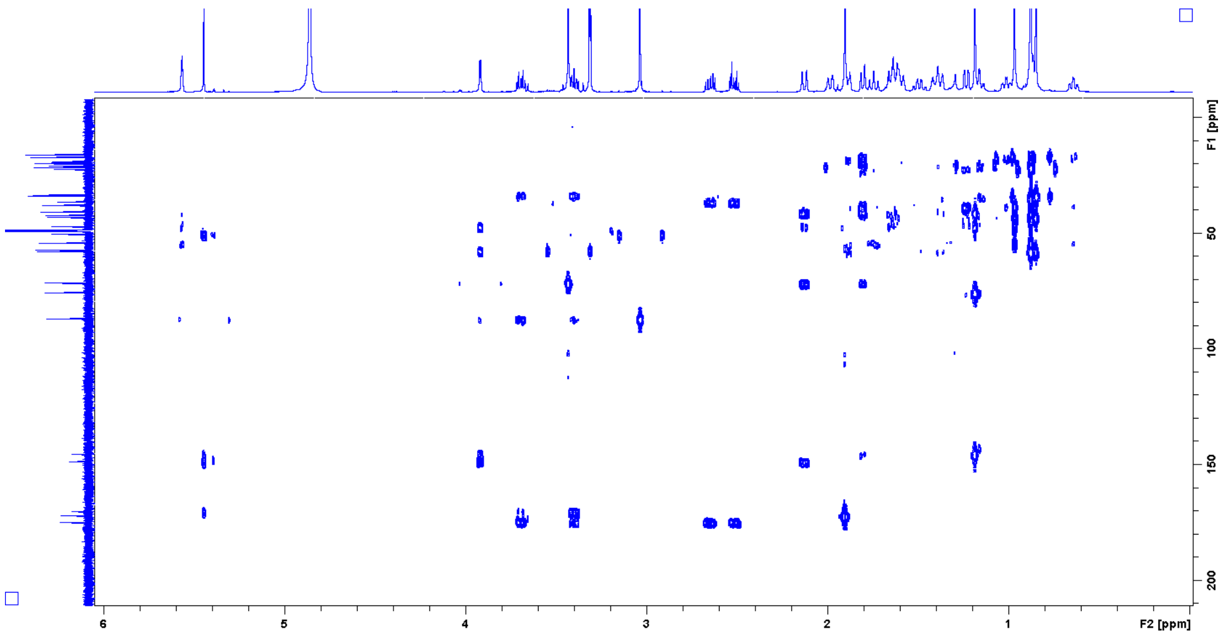

Figure S57. HMBC spectrum of scalimide H (**8**) in CD_3OD (600 MHz).

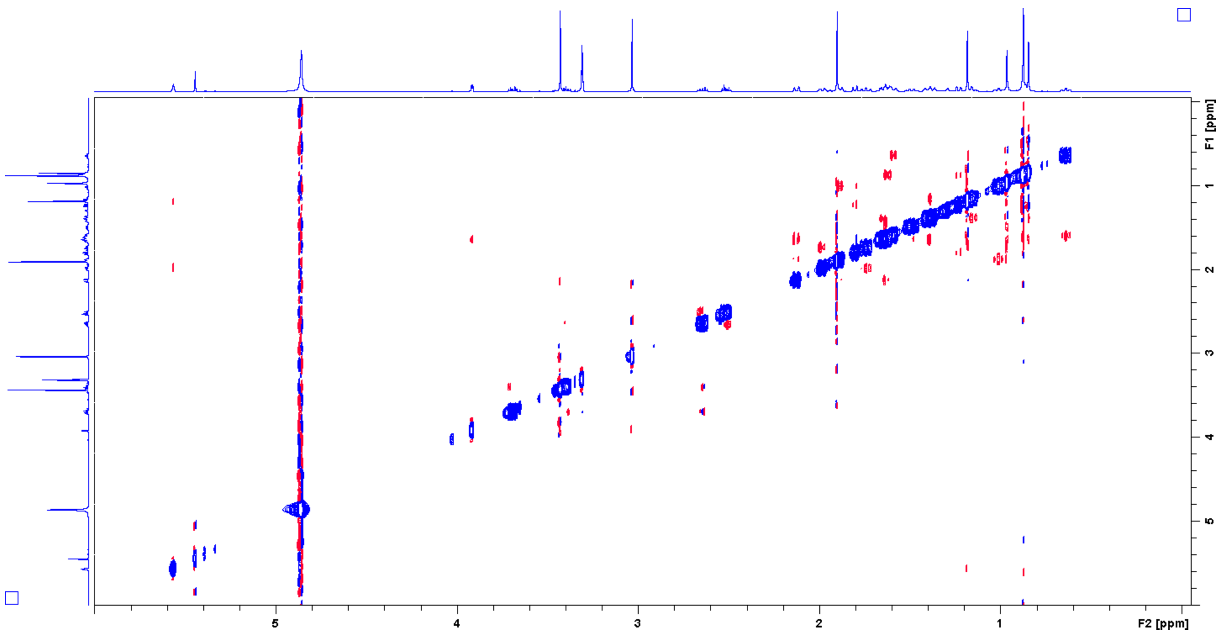

Figure S58. NOESY spectrum of scalimide H (**8**) in CD_3OD (600 MHz).

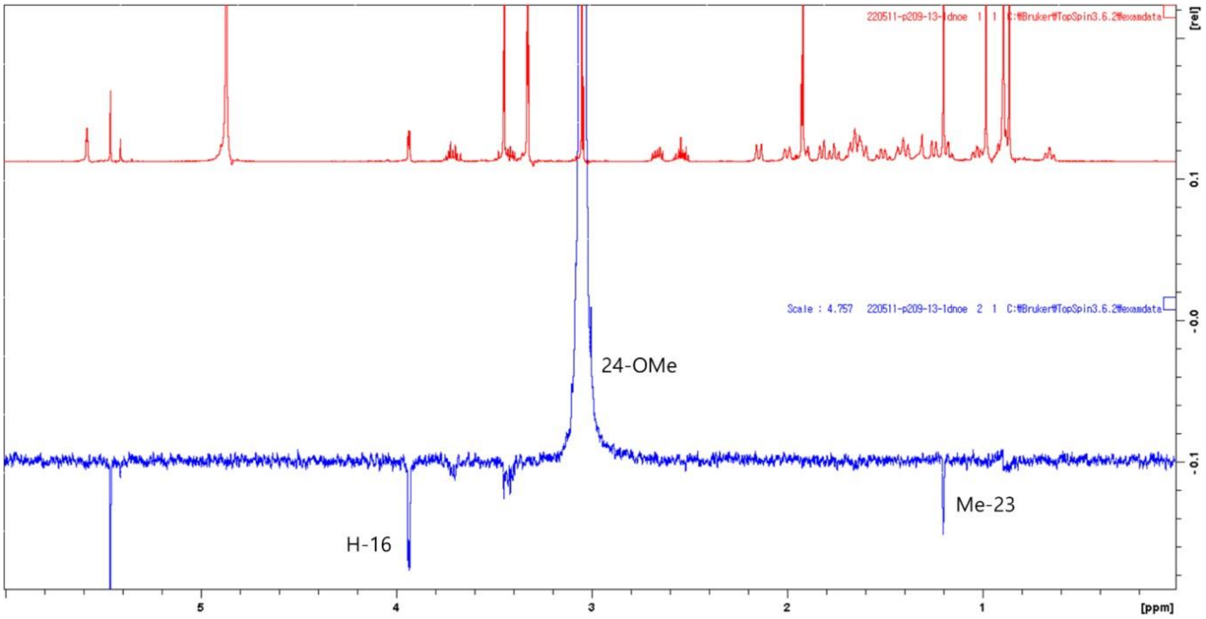

Figure S59. 1D NOE spectrum of scalimide H (**8**).

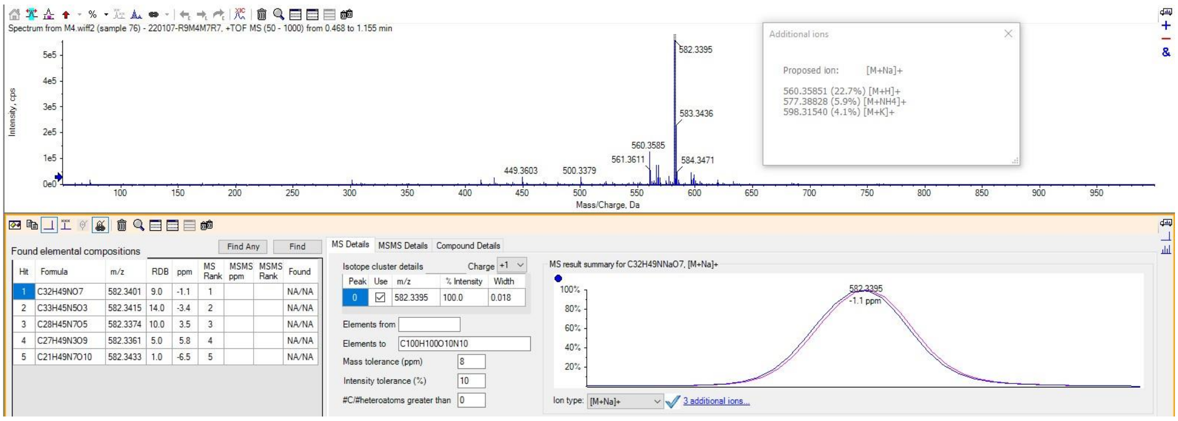

Figure S60. HRMS spectrum of scalimide H (**8**).

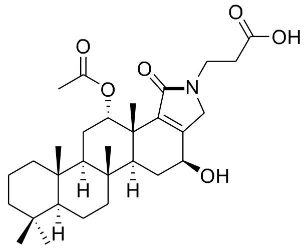

9

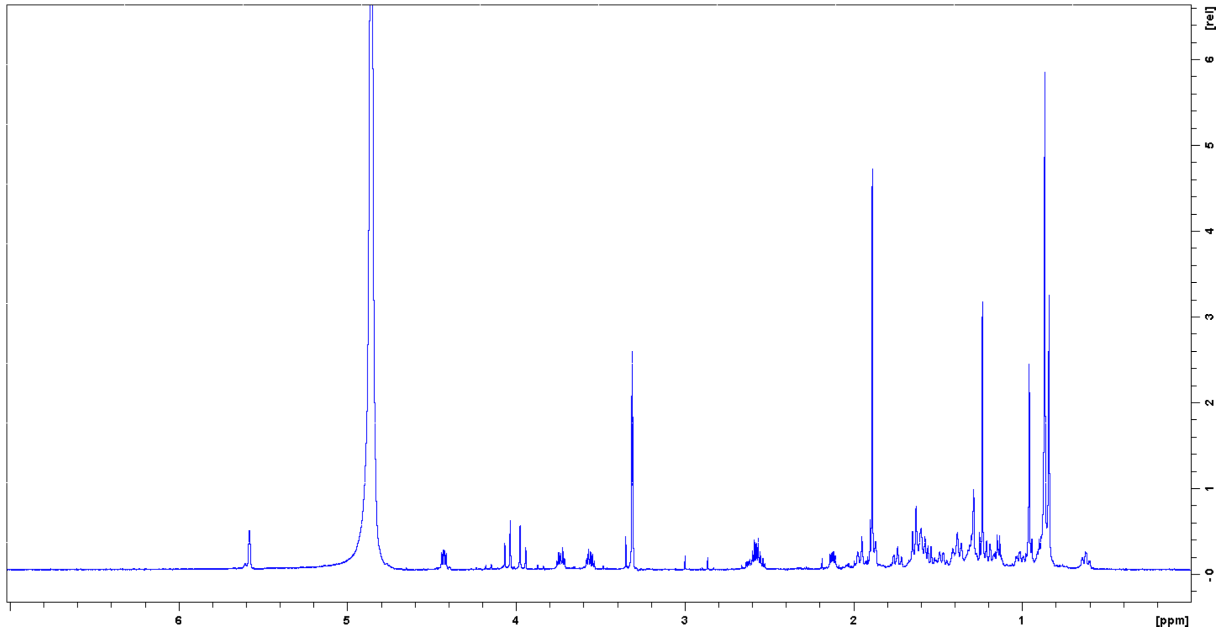

Figure S61. ^{1}H NMR spectrum of scalimide I (**9**) in CD_3OD (600 MHz).

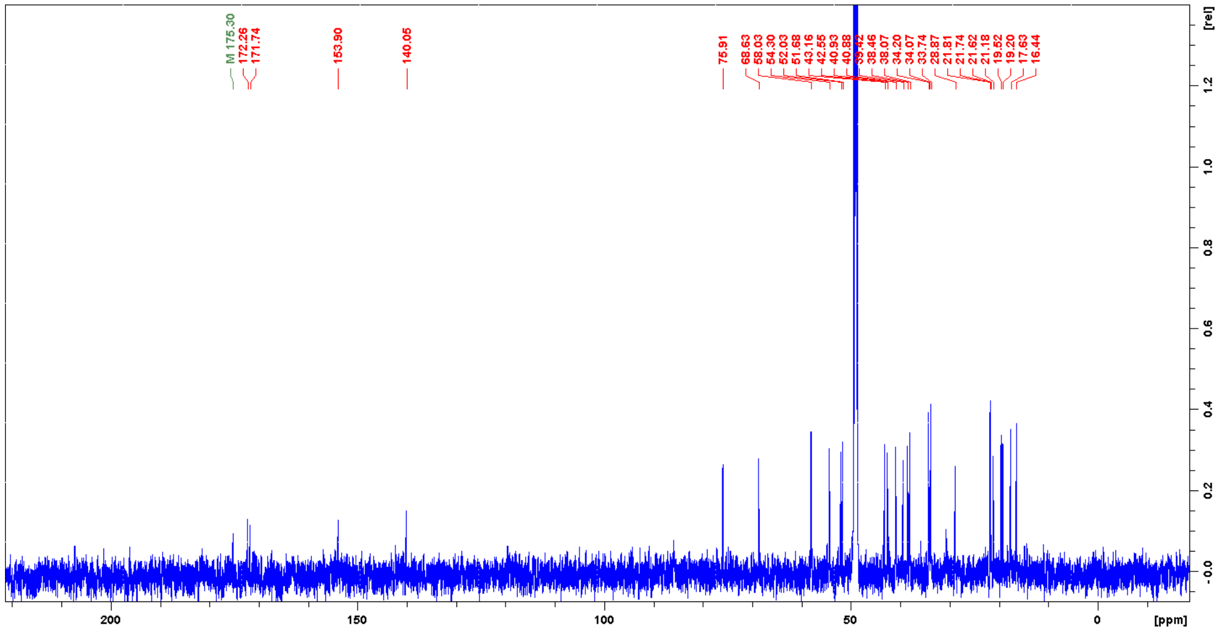

Figure S62. ^{13}C NMR spectrum of scalimide I (**9**) in CD_3OD (150 MHz).

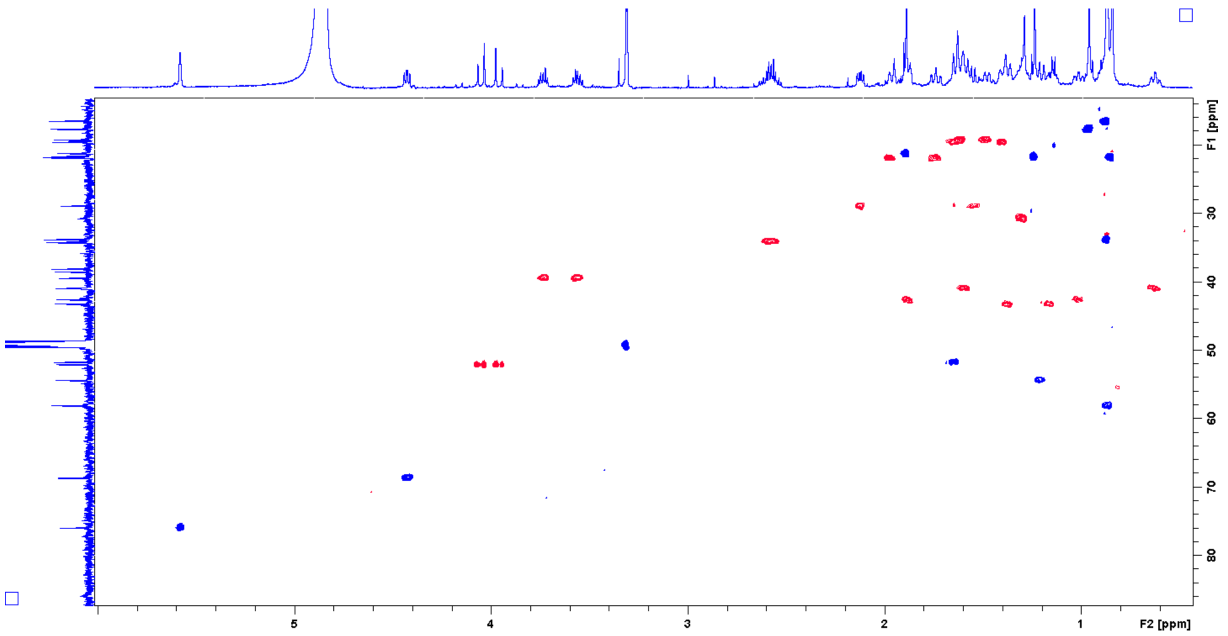

Figure S63. HSQC spectrum of scalimide I (**9**) in CD_3OD (600 MHz).

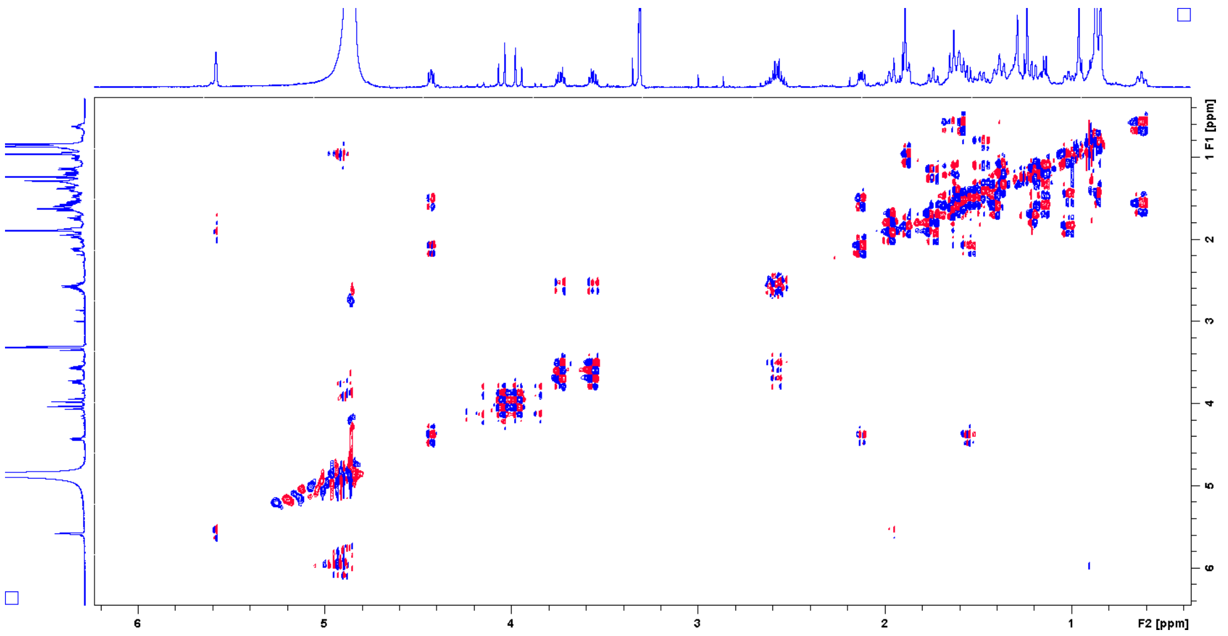

Figure S64. ^1H-^1H COSY spectrum of scalimide I (**9**) in CD_3OD (600 MHz).

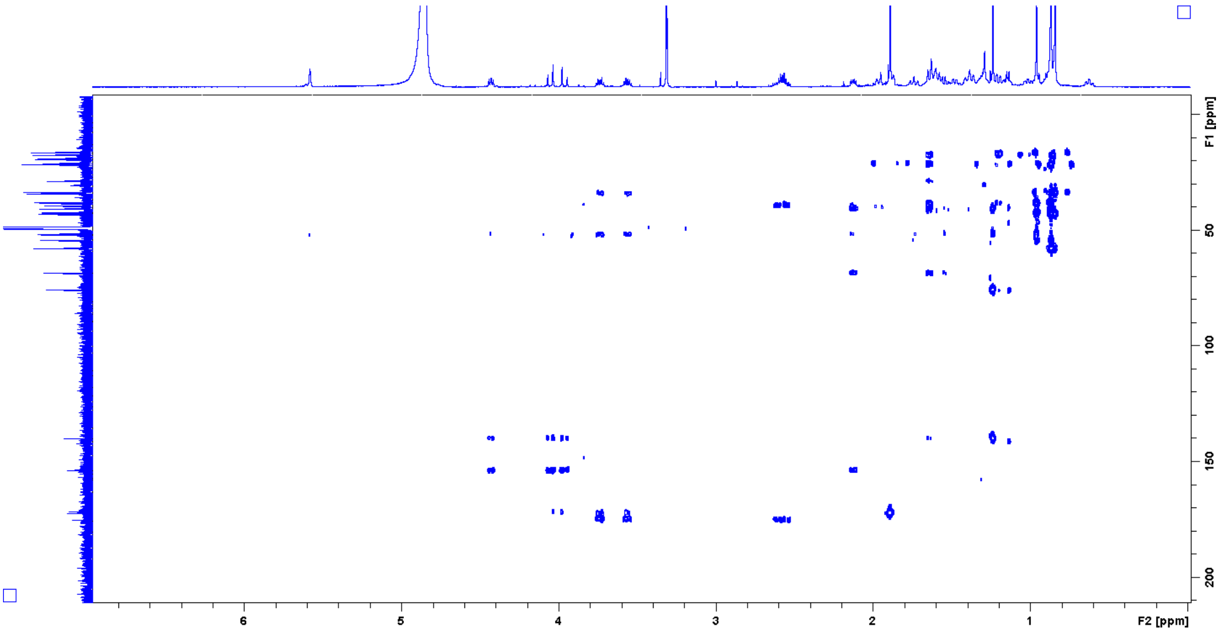

Figure S65. HMBC spectrum of scalimide I (**9**) in CD_3OD (600 MHz).

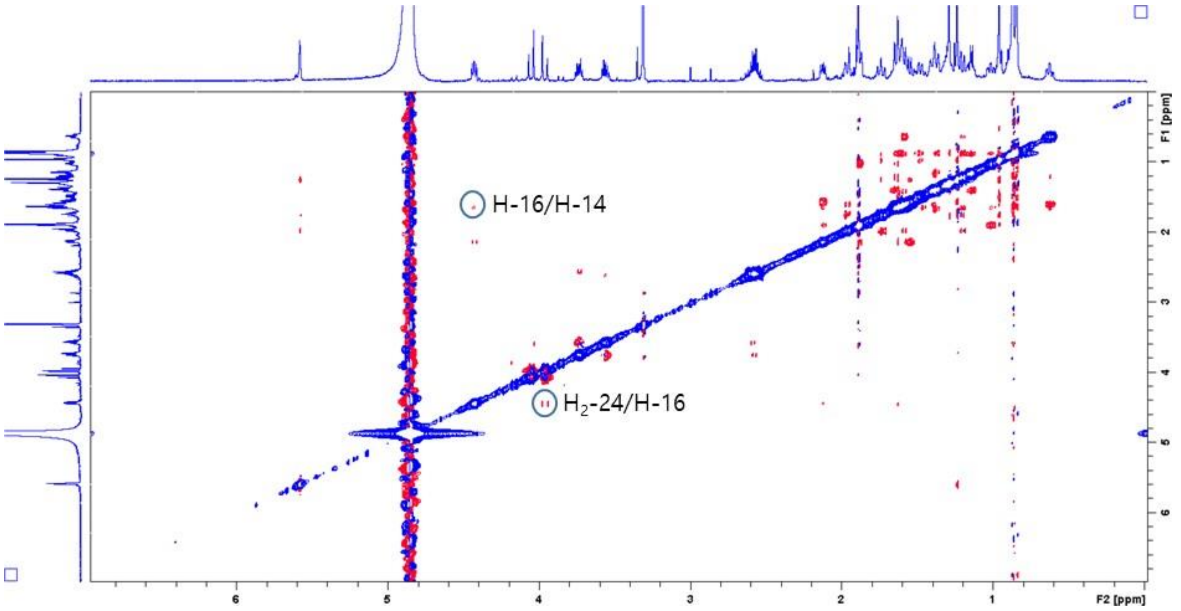

Figure S66. NOESY spectrum of scalimide I (**9**) in CD_3OD (600 MHz).

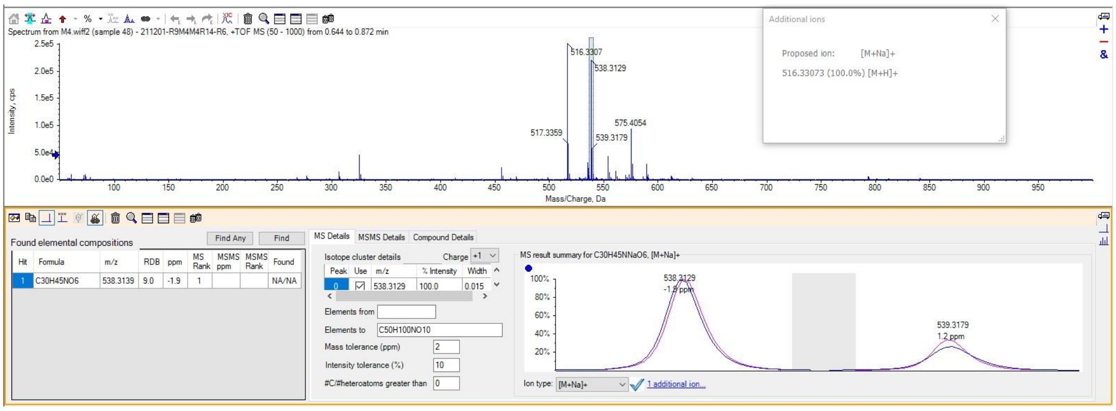

Figure S67. HRMS spectrum of scalimide I (**9**).

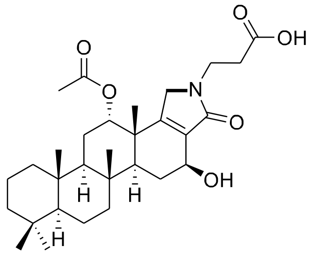

10

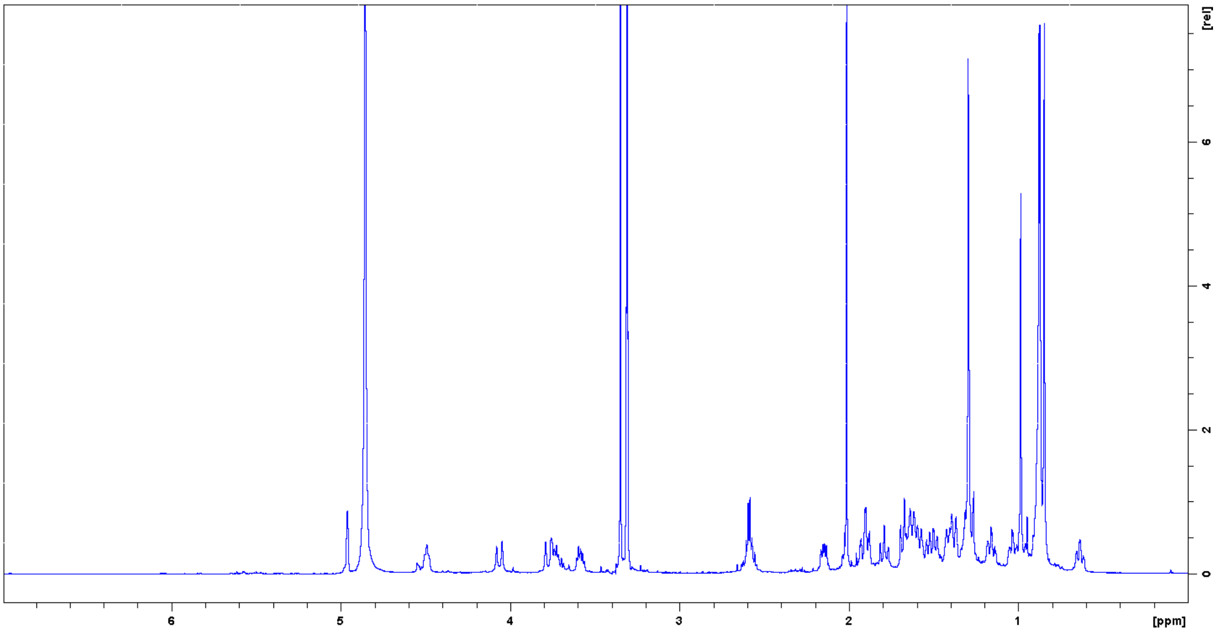

Figure S68. ^{1}H NMR spectrum of scalimide J (**10**) in CD_3OD (600 MHz).

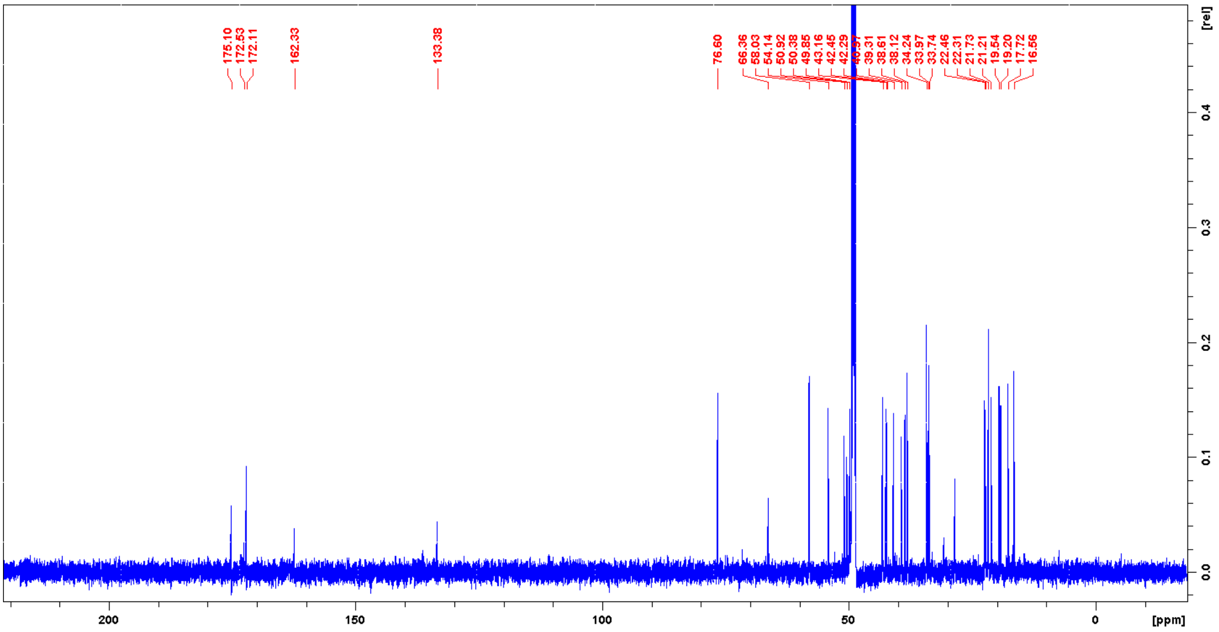

Figure S69. ^{13}C NMR spectrum of scalimide J (**10**) in CD_3OD (150 MHz).

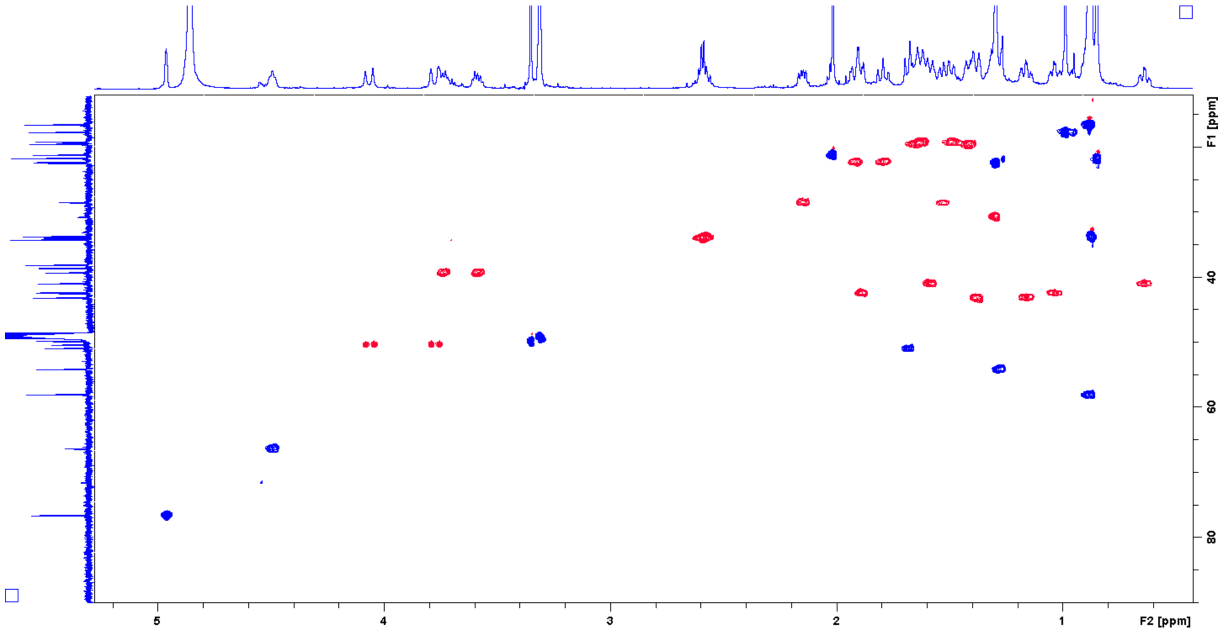

Figure S70. HSQC spectrum of scalimide J (**10**) in CD_3OD (600 MHz).

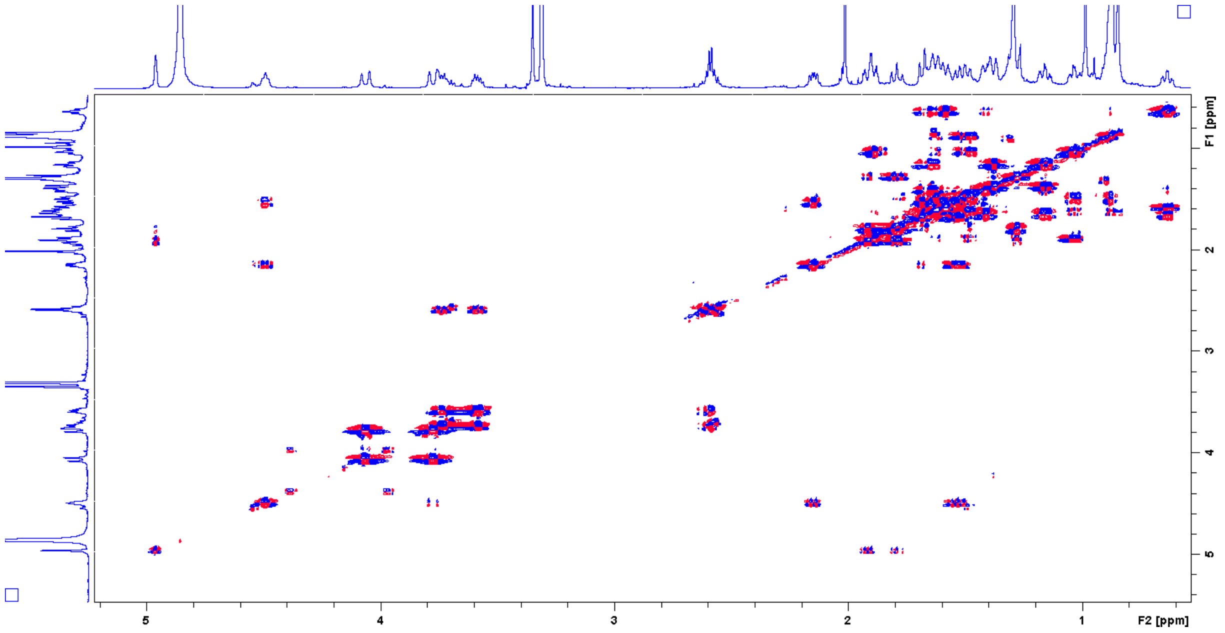

Figure S71. ^{1}H-^{1}H COSY spectrum of scalimide J (**10**) in CD_3OD (600 MHz).

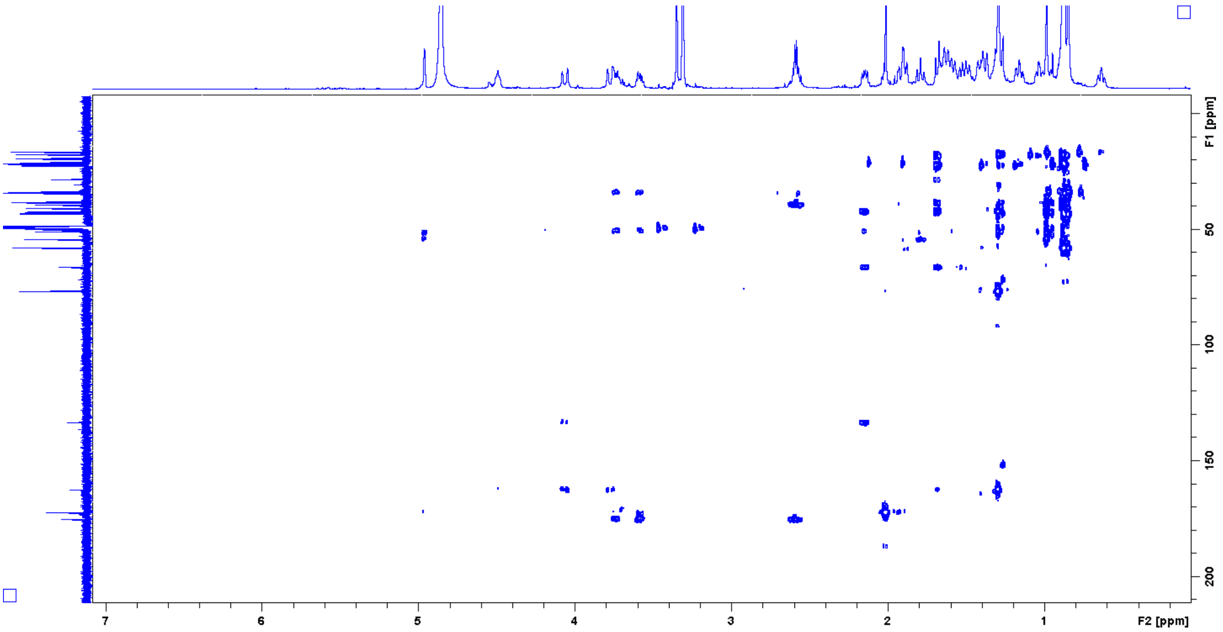

Figure S72. HMBC spectrum of scalimide J (**10**) in CD_3OD (600 MHz).

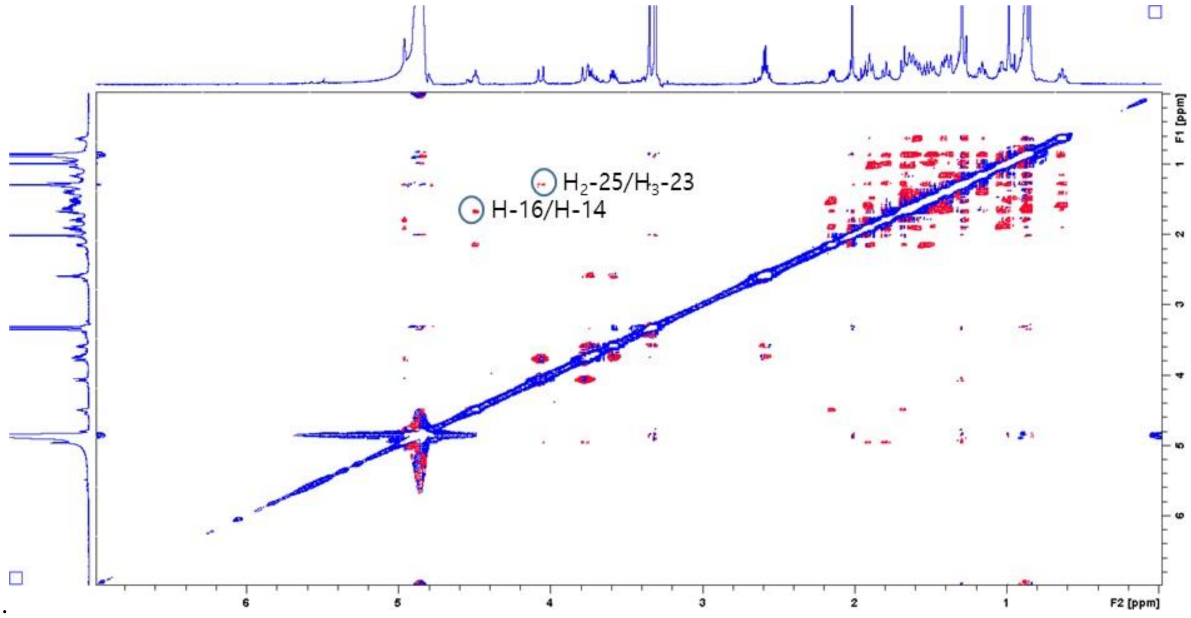

Figure S73. NOESY spectrum of scalimide J (**10**) in CD_3OD (600 MHz).

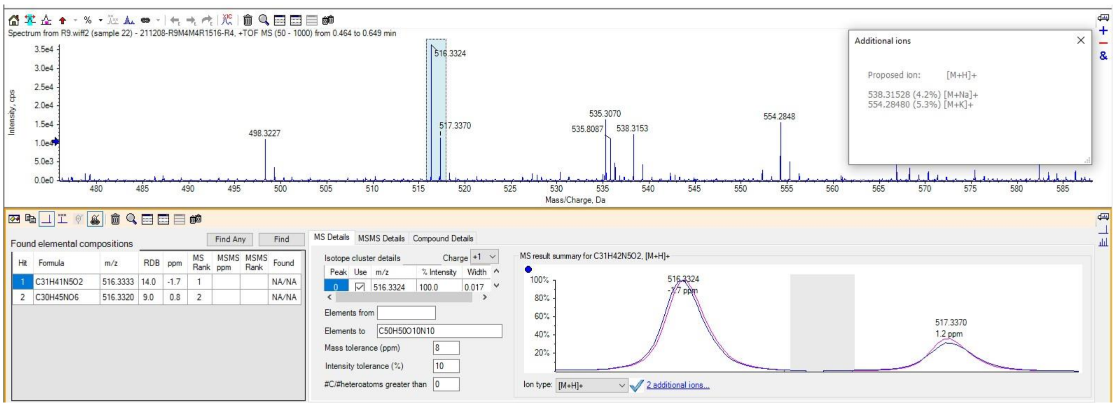

Figure S74. HRMS spectrum of scalimide J (**10**).

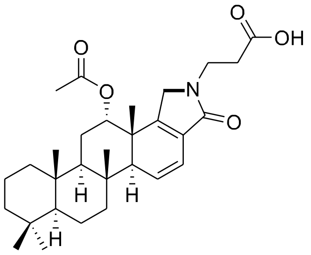

11

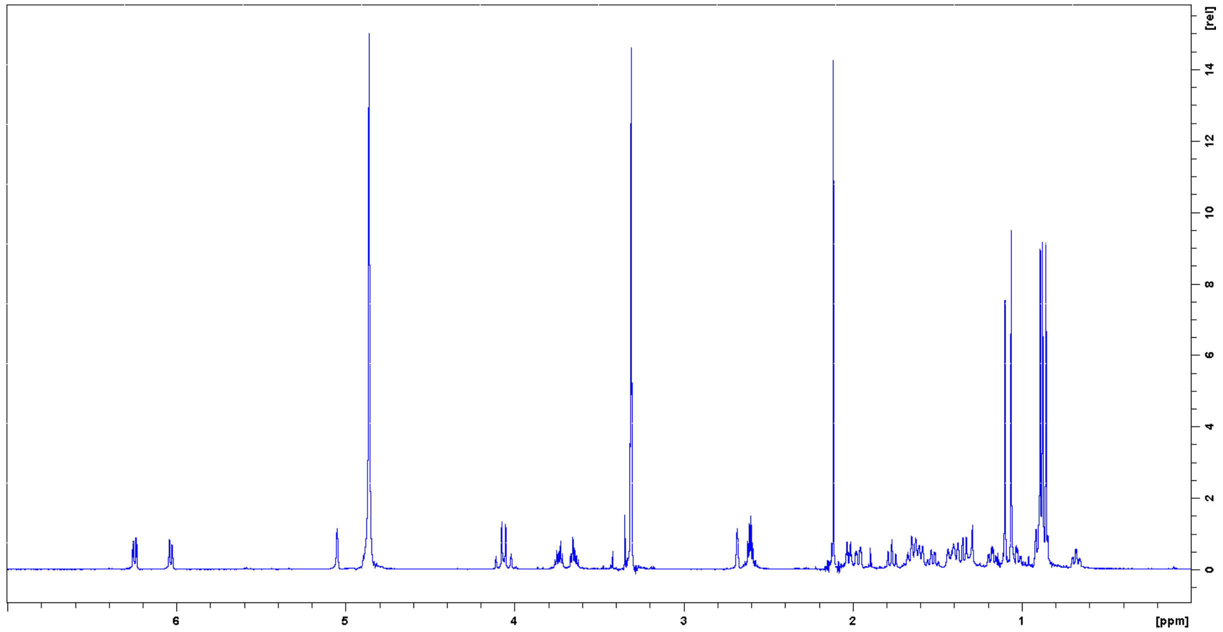

Figure S75. ^{1}H NMR spectrum of scalimide K (**11**) in CD_3OD (600 MHz).

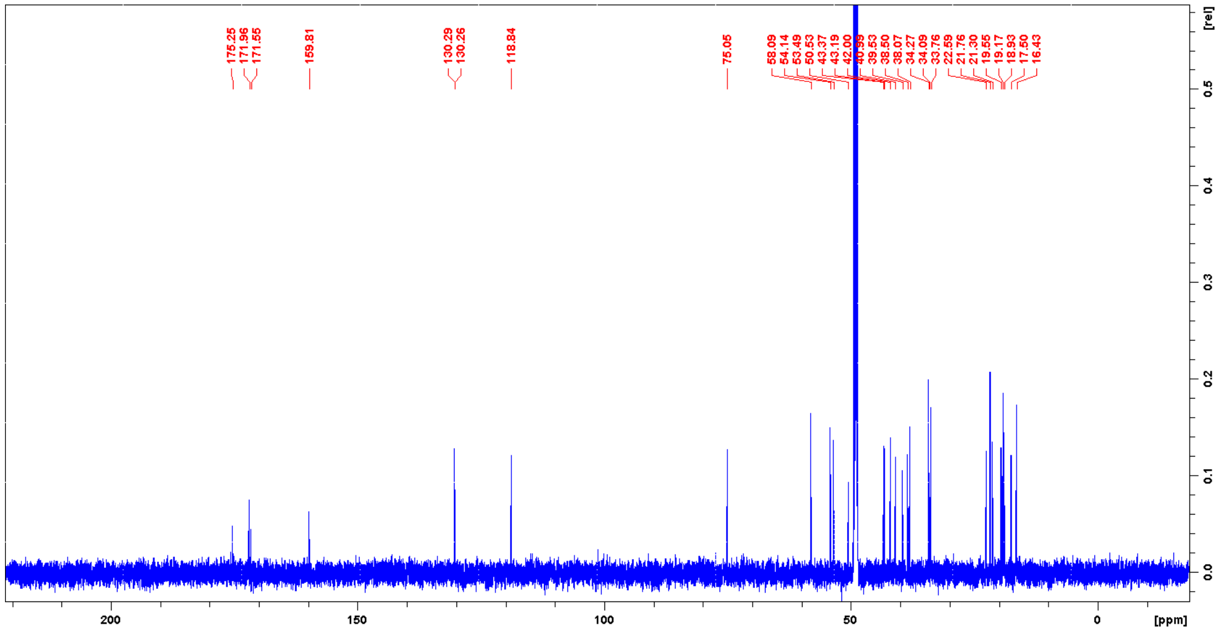

Figure S76. ^{13}C NMR spectrum of scalimide K (**11**) in CD_3OD (150 MHz).

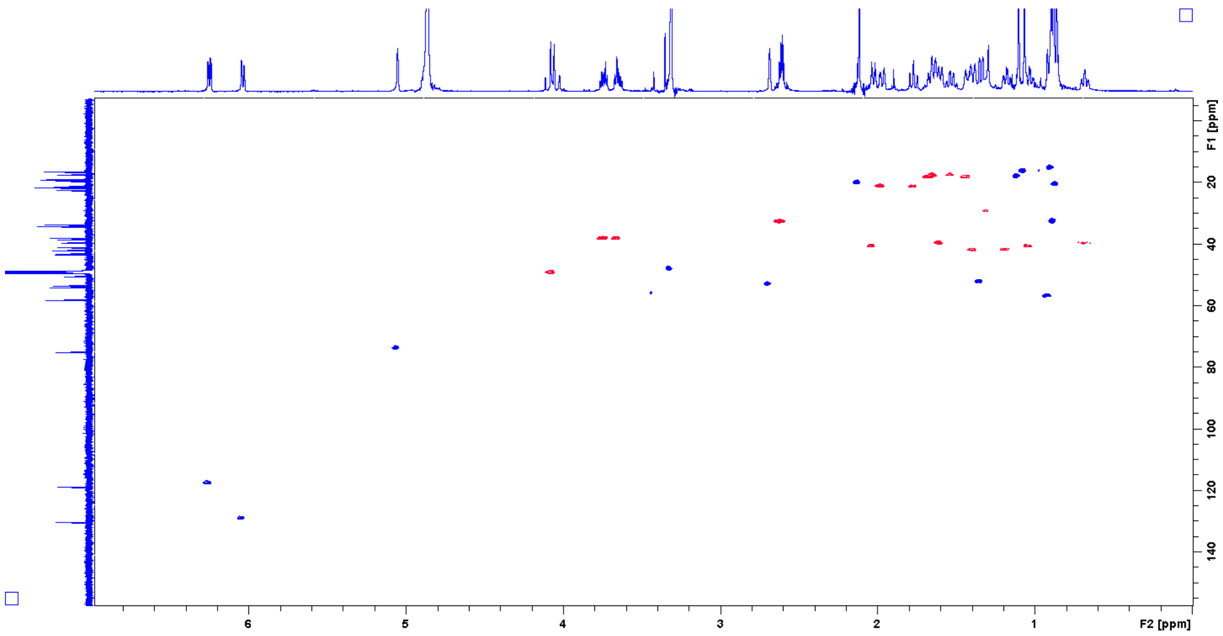

Figure S77. HSQC spectrum of scalimide K (**11**) in CD_3OD (600 MHz).

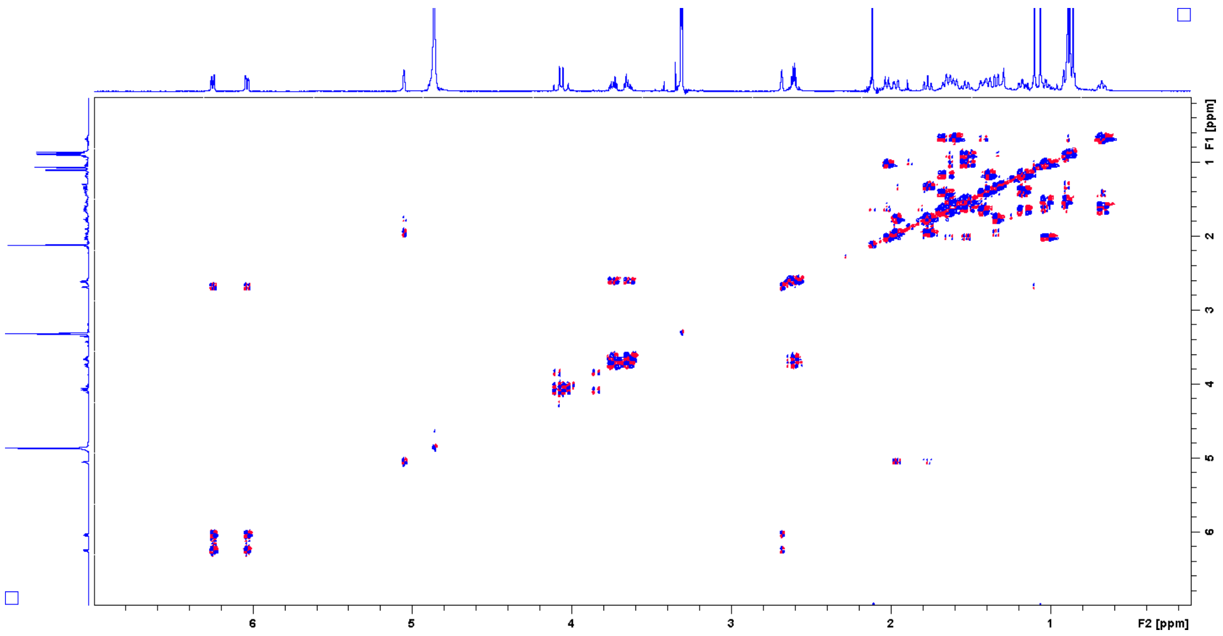

Figure S78. ^1H-^1H COSY spectrum of scalimide K (**11**) in CD_3OD (600 MHz).

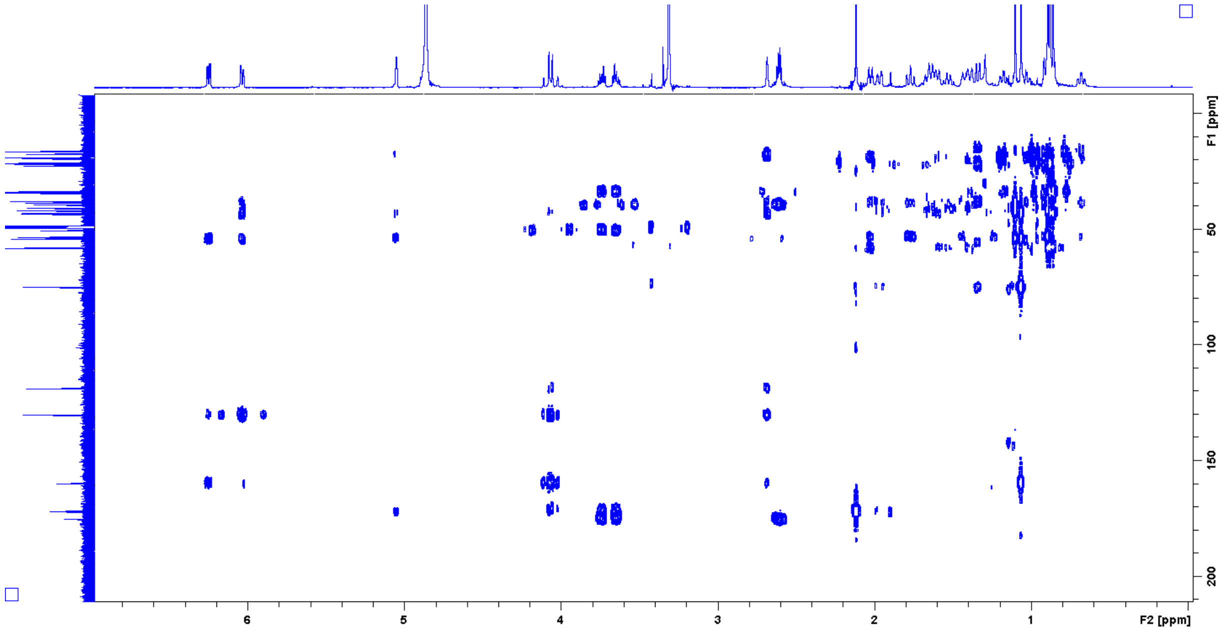

Figure S79. HMBC spectrum of scalimide K (**11**) in CD_3OD (600 MHz).

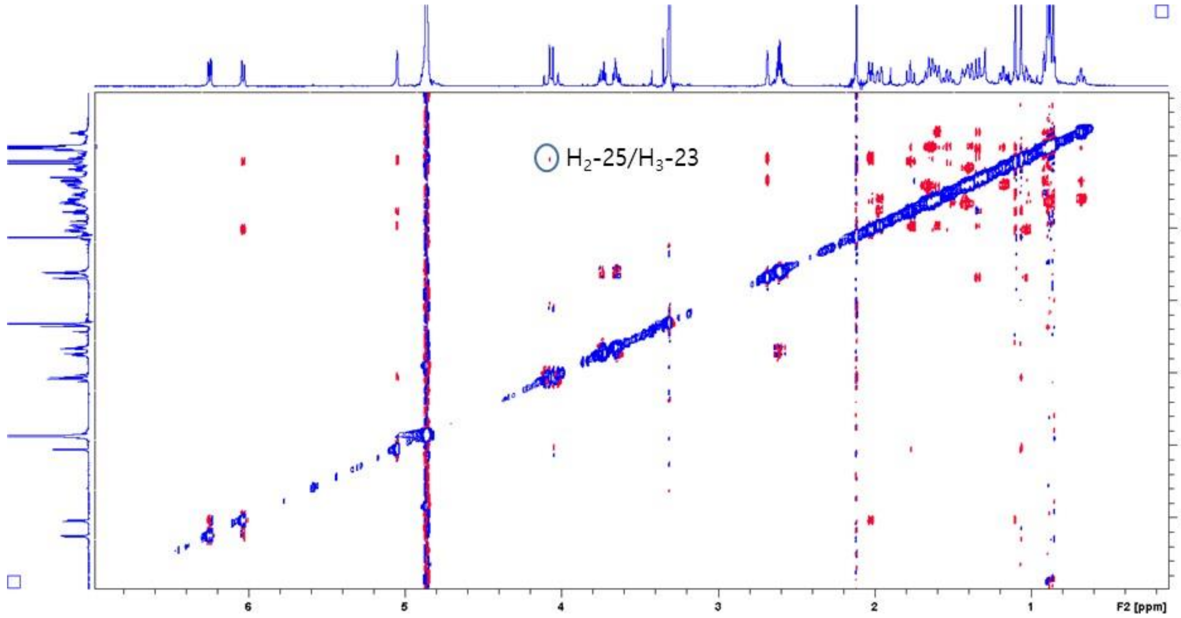

Figure S80. NOESY spectrum of scalimide K (**11**) in CD_3OD (600 MHz).

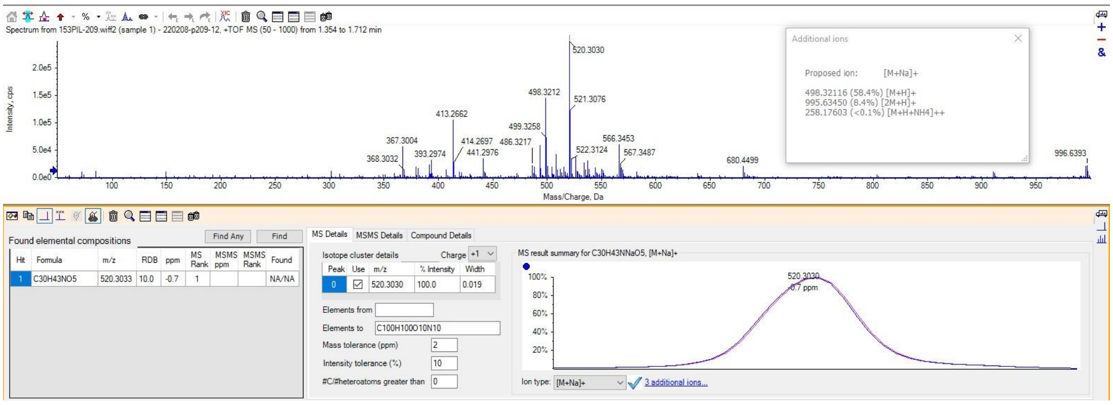

Figure S81. HRMS spectrum of scalimide K (**11**).

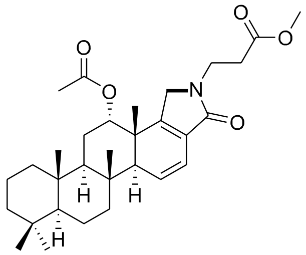

12

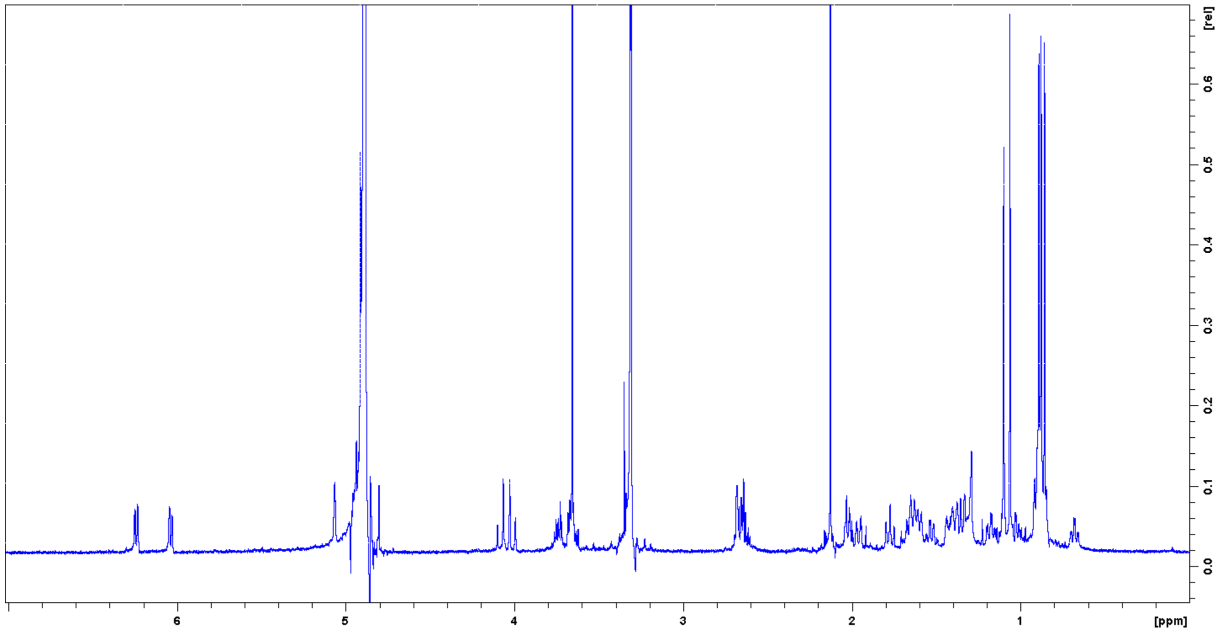

Figure S82. ^{1}H NMR spectrum of scalimide L (**12**) in CD_3OD (600 MHz).

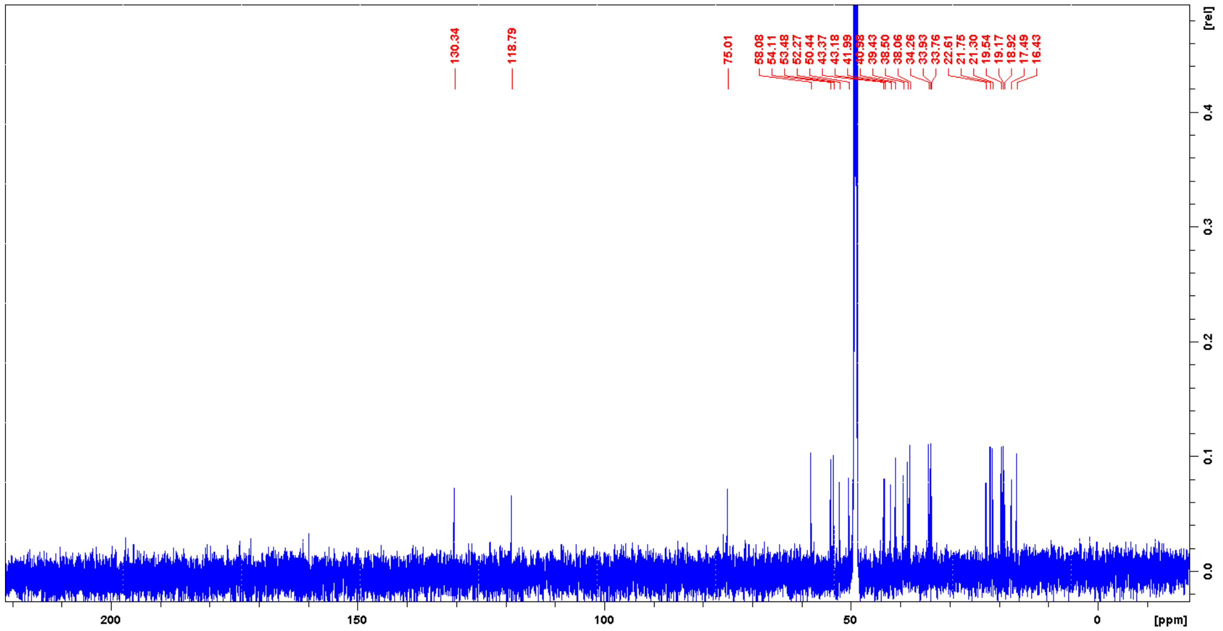

Figure S83. ^{13}C NMR spectrum of scalimide L (**12**) in CD_3OD (150 MHz).

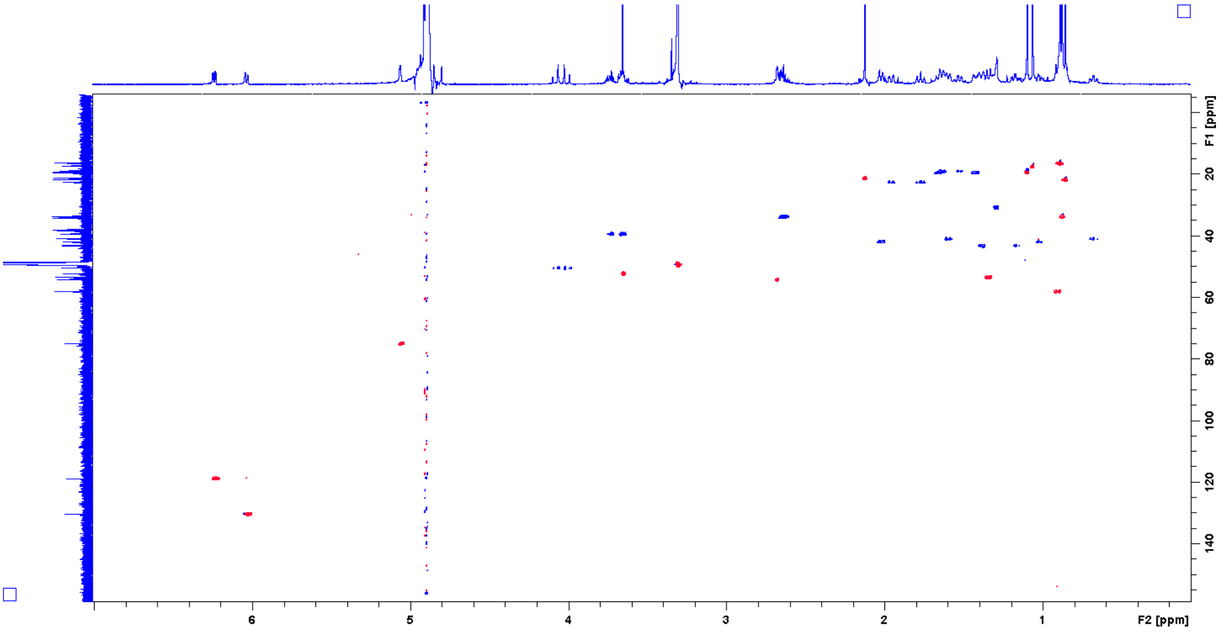

Figure S84. HSQC spectrum of scalimide L (**12**) in CD_3OD (600 MHz).

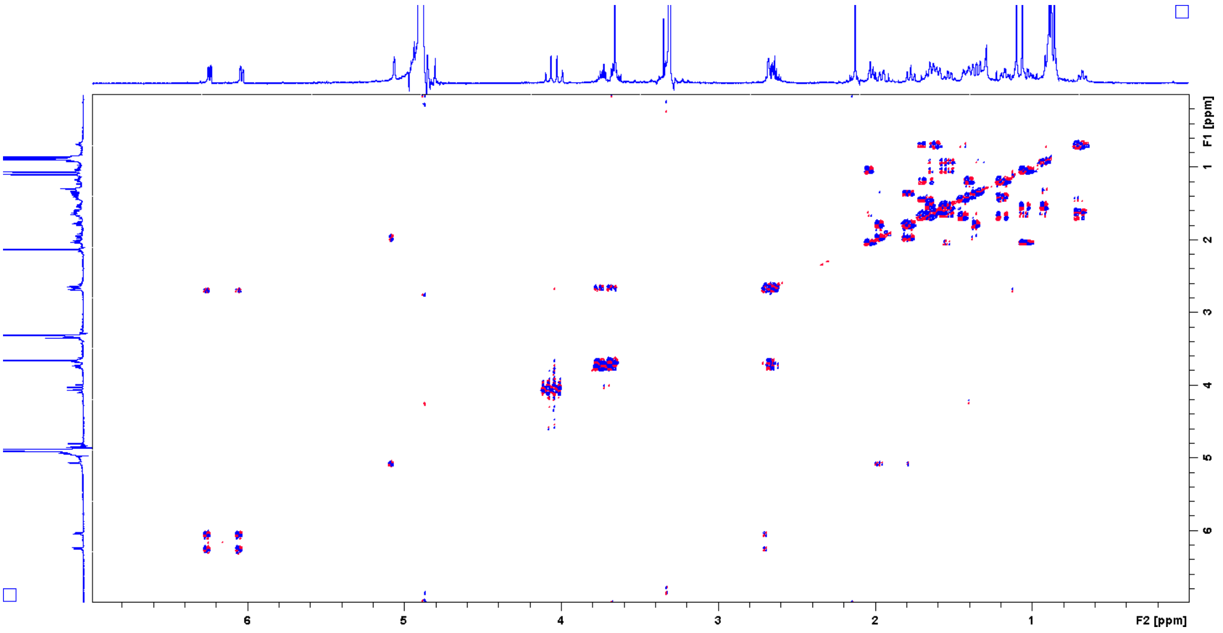

Figure S85. ^1H-^1H COSY spectrum of scalimide L (**12**) in CD_3OD (600 MHz).

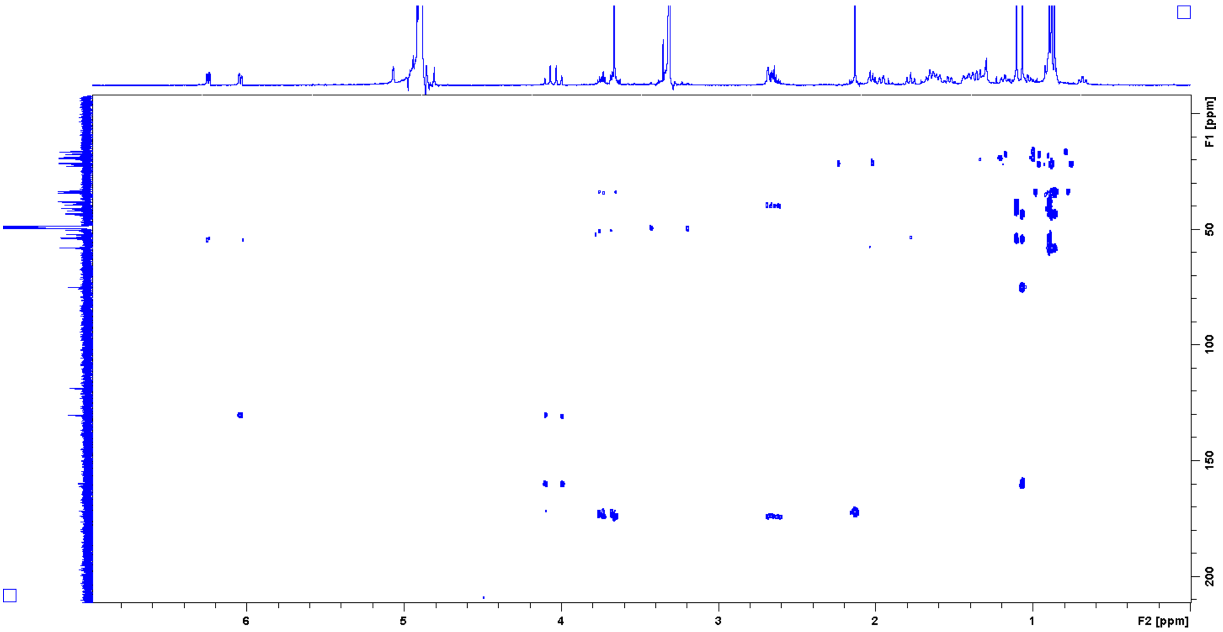

Figure S86. HMBC spectrum of scalimide L (**12**) in CD_3OD (600 MHz).

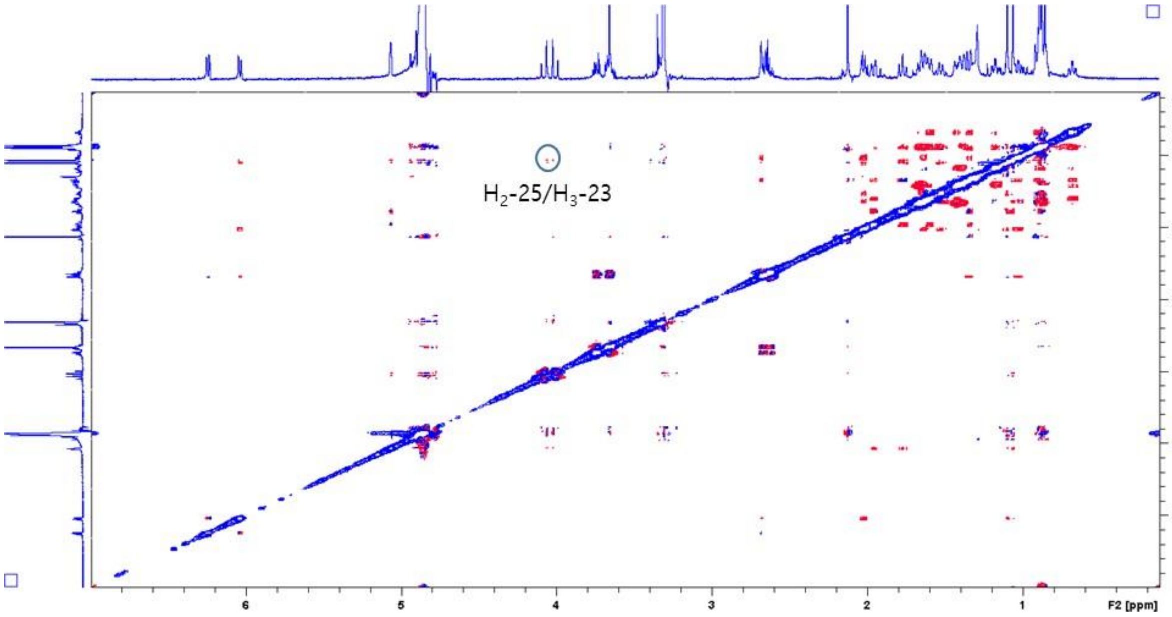

Figure S87. NOESY spectrum of scalimide L (**12**) in CD_3OD (600 MHz).

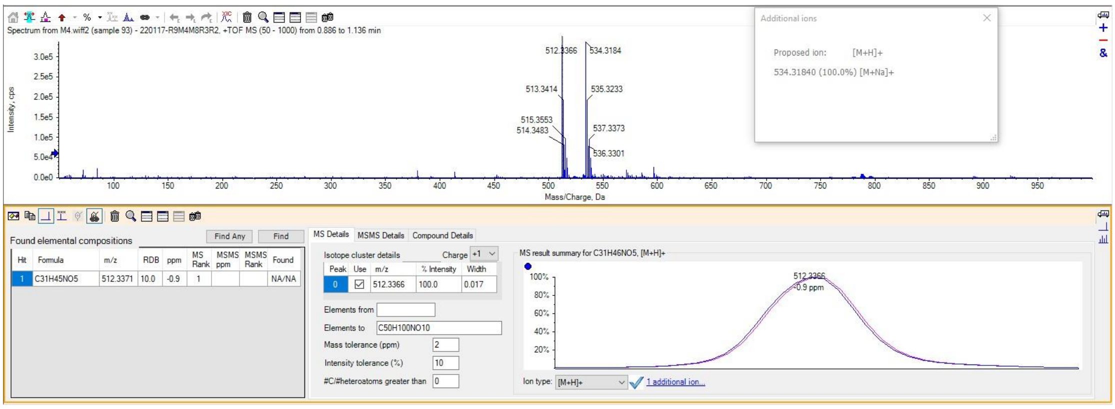

Figure S88. HRMS spectrum of scalimide L (**12**).
